# d–d Dative Bonding Between Iron and the Alkaline‐Earth Metals Calcium, Strontium, and Barium

**DOI:** 10.1002/anie.202005774

**Published:** 2020-07-02

**Authors:** Philipp Stegner, Christian Färber, Jan Oetzel, Ulrich Siemeling, Michael Wiesinger, Jens Langer, Sudip Pan, Nicole Holzmann, Gernot Frenking, Uta Albold, Biprajit Sarkar, Sjoerd Harder

**Affiliations:** ^1^ Chair of Inorganic and Organometallic Chemistry Universität Erlangen-Nürnberg Egerlandstrasse 1 91058 Erlangen Germany; ^2^ Institute of Chemistry University of Kassel Heinrich-Plett-Str. 40 34132 Kassel Germany; ^3^ Fachbereich Chemie Philipps-Universität Marburg Hans-Meerwein-Str. 4 35043 Marburg Germany; ^4^ Research Center for Computer-Aided Drug Discovery Shenzhen Institutes of Advanced Technology Chinese Academy of Sciences Shenzhen 518055 China; ^5^ Institute of Advanced Synthesis School of Chemistry and Molecular Engineering Jiangsu National Synergetic Innovation Center for Advanced Materials Nanjing Tech University Nanjing 211816 China; ^6^ Institut für Chemie und Biochemie Freie Universität Berlin Fabeckstraße 34–36 14195 Berlin Germany; ^7^ Chair of Inorganic Coordination Chemistry Institut für Anorganische Chemie Universität Stuttgart Pfaffenwaldring 55 70569 Stuttgart Germany

**Keywords:** alkaline-earth metals, ferrocene, metal–metal bonding, theoretical chemistry

## Abstract

Double deprotonation of the diamine 1,1′‐(*t*BuCH_2_NH)‐ferrocene (**1**‐H_2_) by alkaline‐earth (Ae) or Eu^II^ metal reagents gave the complexes **1**‐Ae (Ae=Mg, Ca, Sr, Ba) and **1**‐Eu. **1**‐Mg crystallized as a monomer while the heavier complexes crystallized as dimers. The Fe⋅⋅⋅Mg distance in **1**‐Mg is too long for a bonding interaction, but short Fe⋅⋅⋅Ae distances in **1**‐Ca, **1**‐Sr, and **1**‐Ba clearly support intramolecular Fe⋅⋅⋅Ae bonding. Further evidence for interactions is provided by a tilting of the Cp rings and the related ^1^H NMR chemical‐shift difference between the Cp α and β protons. While electrochemical studies are complicated by complex decomposition, UV/Vis spectral features of the complexes support Fe→Ae dative bonding. A comprehensive bonding analysis of all **1**‐Ae complexes shows that the heavier species **1**‐Ca, **1**‐Sr, and **1**‐Ba possess genuine Fe→Ae bonds which involve vacant d‐orbitals of the alkaline‐earth atoms and partially filled d‐orbitals on Fe. In **1**‐Mg, a weak Fe→Mg donation into vacant p‐orbitals of the Mg atom is observed.

## Graphical abstract

**To d or not to d**: Ferrocene complexes of Ca, Sr, and Ba show clear evidence for Fe⋅⋅⋅metal bonding while no such interaction is present in the Mg complex. Analysis with the QTAIM and EDA‐NOCV methods points to Fe→Ae bonds that involve vacant d‐orbitals of alkaline‐earth metals.

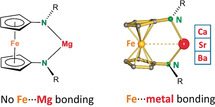

## Introduction

Rigid and redox‐active ferrocene is often used as a building block for chelating ligands.^1^ The bidentate ligand 1,1′‐bis(diphenylphosphino)ferrocene (dppf) has made history in catalysis.^2^ One of the intriguing properties of 1,1′‐bis(donor)ferrocene ligands is their ability to act as tridentate pincer ligands.^3^ First observations of this unusual bonding mode date back to the isolation of an apparent ligand‐deficient Pd complex (**I** 
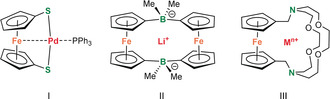
).^4^ The striking reluctancy of this complex to bind an additional donor was explained by Fe→Pd dative bonding which was confirmed by its crystal structure. This κ^3^‐coordination mode of ferrocene‐based ligands has been the subject of intensive studies. The majority of these studies concern late transition‐metal complexes (Ni, Pd, Pt, Fe, Ru, Mn) and only few early d‐block metal complexes (Ti) have been discussed.3a Lanthanide complexes represent an exception and have been separately reviewed.^5^ In contrast, complexes of the main‐group metals are hardly known and Fe⋅⋅⋅metal interactions are only rarely and, as expected for weak bonding, often carefully discussed.^6^, ^7^, ^8^, ^9^, ^10^, ^11^, ^12^, ^13^, ^14^, ^15^ This holds especially for Fe interactions with s‐block metals.^9^, ^10^, ^11^, ^12^, ^13^ The Wagner group has reported a borate‐bridged ferrocene (**II**), which is a highly efficient Li^+^ scavenger likely because of Fe⋅⋅⋅Li^+^ interactions.^9^ Plenio et al. published cryptand‐bridged ferrocene complexes (**III**)^11^ and observed that, although Na^+^ and Ca^2+^ have nearly equal ionic radii (1.02 Å vs. 1.00 Å),^16^ its sodium complex^13^ shows a clearly longer Fe⋅⋅⋅metal distance than its calcium complex (4.387(4) Å vs. 3.658(6) Å). It was concluded that *“this may or may not be viewed as an indication for Fe→Ca* ^*2+*^ *interaction”* but such bonding was not supported by UV studies.^11^

Our interest in Fe→Ae^2+^ bonding (Ae=alkaline‐earth) is motivated by the growing evidence that d‐orbitals on the heavier Ae metals Ca, Sr, and Ba may play a crucial role in bonding and molecule activation. Older theoretical work underlines the subtle effect of d‐orbital contributions.^17^, ^18^ Most recently, experimental evidence for Ae(CO)_8,_ 19 Ae(N_2_)_8_,^20^ and Ae(C_6_H_6_)_3_ 21 complexes was reported. Also, in these transition‐metal‐like complexes, bonding is explained using Ae‐metal d‐orbitals. Although the latter species are formally electron‐rich Ae^0^ complexes, computational analyses of the Ca^2+^⋅⋅⋅(C_6_H_6_) bond^22^ or a Ca^2+^ hydride cluster^23^ also suggest d‐orbital participation.

Herein, we provide experimental evidence for Fe⋅⋅⋅Ae bonding and illustrate that, while for Mg such bonding is essentially absent, heavier Ae metals display short Fe⋅⋅⋅Ae contacts. Extensive bonding analysis with QTAIM (quantum theory of atoms in molecules)^24^ and EDA‐NOCV (energy decomposition analysis with natural orbitals for chemical valence)^25^ provides evidence for significant d‐orbital contributions of the Ae^2+^ ions.

## Results and Discussion

**Scheme 1:**
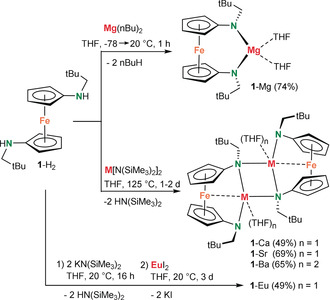
Syntheses of alkaline‐earth metal complexes **1**‐Ae (Ae=Mg, Ca, Sr, Ba) and the Eu^II^ complex **1**‐Eu with the chelating diamido ligand **1**.

The magnesium complex **1**‐Mg was prepared by double deprotonation of the diamine ligand **1**‐H_2_ 12 by Mg*n*Bu_2_ at low temperature (Scheme 1). The heavier homologues **1**‐Ae (Ae=Ca, Sr, Ba) were obtained by deprotonation of **1**‐H_2_ with the weaker bases Ae[N(SiMe_3_)_2_]_2_ under more stringent conditions. While the color of **1**‐Mg is a pale orange‐red, crystals of **1**‐Ae (Ae=Ca, Sr, Ba) are intensely bright red. Since lanthanide(II) complexes show structural features that are remarkably similar to complexes of equally sized Group 2 metal ions (for example, Yb^2+^/Ca^2+^ and Eu^2+^/Sr^2+^),^26^ we also prepared **1**‐Eu. While the complexes **1**‐Ae (Ae=Ca, Sr, Ba) are completely insoluble in aromatic solvents or in THF, the Eu^II^ complex is much better soluble and could therefore be prepared in 49 % yield using the salt‐metathesis route, that is, by reacting **1**‐K_2_ with EuI_2_ and extraction of **1**‐Eu from insoluble KI with THF.

**Figure 1:**
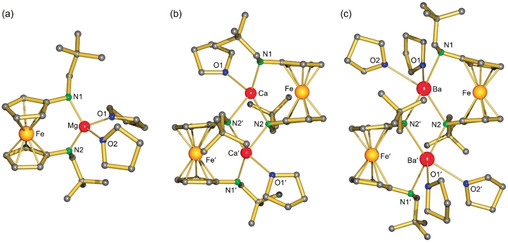
Representative crystal structures: a) **1**‐Mg, b) **1**‐Ca, c) **1**‐Ba.

The magnesium complex **1**‐Mg crystallized as a monomer with two THF ligands (Figure 1). Complexes with larger metals crystallized as centrosymmetric dimers in which the number of THF ligands increases with metal size (**1**‐Ca, **1**‐Sr, and **1**‐Ba). The M−N and M−O bonds are in the expected range (for selected geometric parameters, see Table S1 in the Supporting Information).

The M⋅⋅⋅Fe distances (Table 1) are a measure for potential M⋅⋅⋅Fe interactions. All complexes, apart from **1**‐Mg, show M⋅⋅⋅Fe distances that are similar to the sum of the covalent metal radii.^27^ Although this method comes with limitations, it is generally accepted for the assessment of metal–metal interactions.^28^ While Ba^2+^ is nearly twice the size of Mg^2+^ (Table 1), the Mg⋅⋅⋅Fe and Ba⋅⋅⋅Fe distances are similar. This is a clear indication for a prominent Ba⋅⋅⋅Fe interaction. Subtracting the ionic radii for M^2+^ from the M⋅⋅⋅Fe distance gave a similar value of circa 2.1 Å for all structures, except for **1**‐Mg where a value of circa 2.7 Å was found. It can therefore be concluded that in **1**‐Mg, there is insignificant Mg⋅⋅⋅Fe bonding. For the heavier congeners, the M⋅⋅⋅Fe distance increases linearly with M^2+^ ion size. As expected, the Eu⋅⋅⋅Fe and Sr⋅⋅⋅Fe distances in **1**‐Eu and **1**‐Sr are equal within standard deviations.

**Table 1:** Selected data for **1**‐H_2_ and metal complexes with the **1** ^2−^ ligand.

Complex	**1**‐H_2_	**1**‐Mg	**1**‐Ca	**1**‐Sr	**1**‐Ba	**1**‐Eu
*d*(Fe⋅⋅⋅M) [Å]	–	3.4255(6)	3.1129(6)	3.3204(5)	3.4537(4)	3.3229(5)
Σ(covalent radii) [Å]^[a]^	–	2.73	3.08	3.27	3.47	3.30
r(M^2+^) 6‐coordinate [Å]^[b]^	–	0.72	1.00	1.18	1.36	1.17
*d*(M⋅⋅⋅Fe)−*r*(M^2+^) [Å]	–	2.706	2.113	2.140	2.094	2.153
Cp/Cp tilt angle [°]	0.00(3)	2.99(7)	7.09(8)	6.97(8)	15.23(11)	7.24(12)
δH_α_/δH_β_ Δδ [ppm]^[c]^	3.82/3.89 0.07	3.86/3.96 0.10	3.83/3.98 0.15	3.73/4.11 0.38	3.60/4.15 0.55	–
Absorbance UV/Vis [nm]^[d]^ *ϵ* [L mol^−1^ cm^−1^]	450 198	455 275	506 602	479 484	474 651	447 679
1^st^ Ox. pot. vs. Fc/Fc^+^ [V]	−0.77	−1.57	−1.67	−1.75	−1.75	–
Fe⋅⋅⋅M DFT [Å]	–	3.259	3.061	3.198	3.442	–
*ρ*(r) bcp Fe⋅⋅⋅M [e Å^−3^]	–	–	0.115	0.101	0.121	–
∇^2^ *ρ*(r) bcp Fe⋅⋅⋅M [e Å^−5^]	–	–	0.964	0.819	0.602	–
*H*(r) bcp Fe⋅⋅⋅M [Hartree Å^−3^]	–	–	−0.005	−0.002	−0.015	–

[a] Covalent radii taken from ref. 27. [b] Ionic radii for 6‐coordinate M^2+^ ions taken from ref. 16. [c] Chemical‐shift differences measured in C_6_D_6_ (**1**‐H_2_), C_6_D_6_/[D_8_]THF (**1**‐Mg) or [D_5_]pyridine (**1**‐Ca, **1**‐Sr, **1**‐Ba). [d] Measured in pyridine solution. The signal for **1**‐Eu is superimposed with the very strong absorbance for Eu^II^ which also forms intensely red metallocenes.

**Scheme 2:**
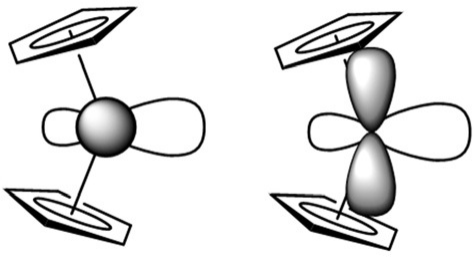
Bending of ferrocene causes deformation of the HOMO frontier orbitals.

Another telltale for Fe⋅⋅⋅metal interactions is the tilt angle between the two Cp least‐squares planes which are perfectly parallel in centrosymmetric **1**‐H_2_ (Figure S17). The interplanar Cp/Cp′ angles gradually increase with metal size: the tilt angle in **1**‐Mg is 2.99(7)°, while the angle in **1**‐Ba is 15.23(11)°. This distortion is accompanied by asymmetric Cp–Fe bonding. The Fe−C bonds in **1**‐Mg vary from 2.027(2)–2.135(2) Å (mean: 2.059 Å), while in **1**‐Ba, a broader range of 1.998(3)–2.300(3) Å (mean: 2.086 Å) is found. The longest Fe−C distance is observed for the substituted Cp carbon which also shows a close contact to Ba (3.093(3) Å). Forcing ferrocene to bend with a tilt angle of 15° is endothermic by circa 7.5 kcal mol^−1^.^29^ Bending causes the HOMO frontier orbitals, which are mainly of d‐character (Scheme 2), to bulge outwards. This causes their energies to rise considerably and improves the electron‐donating abilities of the Fe center. The energy needed for bending ferrocene is therefore compensated for by additional Fe→Ba donor bonding.

NMR data for the diamagnetic Fe^II^ complexes **1**‐Ae support increasing ferrocenyl bending with Ae metal size. The extent of Cp‐ring tilting is related to the ^1^H NMR chemical‐shift (δ) difference between the Cp α and β protons: large tilt angles cause a large Δδ.3a While resonances for H_α_ hardly change, those for H_β_ shift upfield with increased tilting.^28^ Monomeric **1**‐Mg dissolves well in C_6_D_6_/[D_8_]THF and the chemical‐shift difference between the Cp α and β protons is only 0.10 ppm (Table 1). Dimers **1**‐Ae (Ae=Ca, Sr, Ba) are completely insoluble in pure [D_8_]THF and NMR spectra could only be recorded in [D_5_]pyridine at 100 °C. It was found that Δδ increases gradually with metal size. The largest chemical‐shift difference of 0.55 ppm has been measured in **1**‐Ba, which shows the most extreme ferrocenyl bending.

Considering the aforementioned relatively short (Cp)C−Ba distance, additional Cp–Ba bonding seems plausible and would be in line with previous observations. The high‐valent Fe^IV^ dication [Cp*_2_Fe(CO)]^2+^ does not show Fe→π*(CO) backbonding, but a weakening of the CO bond should rather be explained by a Cp→π*(CO) interaction.^30^ Along similar lines, strong tilting in **1**‐Ba could be explained by Cp→Ba instead of Fe→Ba bonding. Comprehensive bonding analysis by DFT methods, however, does not show any indication for Cp→Ba bonding and justifies the herein proposed Fe→Ba donor bond (see below).

Fe−M Interactions can also be identified by UV/Vis spectroscopy.3a Complexes with a (ferrocene)Fe−M bonding mode generally show strong absorption of green light around 500 nm with a high extinction coefficient *ϵ* related to Fe→M charge transfer and are typically intensively red.^31^ The UV/Vis data for **1**‐H_2_ and all metal complexes dissolved in pyridine are summarized in Table 1. While **1**‐H_2_ and complex **1**‐Mg exhibit weak absorptions around 450 nm, complexes with short Fe⋅⋅⋅Ae distances show red‐shifted and stronger absorptions around 500 nm.

The extreme sensitivity of these compounds towards hydrolysis, combined with their very low solubility, pose a challenge for electrochemical measurements. Meticulous drying of the electrochemical cell with *t*BuLi in pentane as well as extensive drying of solvents and the electrolyte are strictly required. Measurements were performed inside a glove box in THF using 0.1 m [Bu_4_N^+^][PF_6_^−^] as electrolyte and the Fc/Fc^+^ couple as reference. The diamine **1**‐H_2_ and the **1**‐Ae complexes displayed a number of redox processes (Figure S30, Table S8). All compounds show either a quasi‐reversible or an irreversible first oxidation step at very negative potentials (Table 1) which is in line with their electron‐rich nature. Whereas **1**‐H_2_ displays its first oxidation step at −0.77 V, the first oxidation potential for **1**‐Mg is clearly lower (−1.57 V) and becomes more negative for complexes with the larger metals (−1.75 V). DFT calculations for **1**‐H_2_ and all **1**‐Ae complexes show very similar HOMOs (Figures S36–S40) that have substantial contributions from the ferrocenylene unit and the N donor atoms. Since removal of an electron from these systems occurs from a similar type of orbital, the increasingly negative oxidation potentials down the Ae metal group are in line with the increasing bond ionicity. Electrochemical measurements also revealed a far better redox stability for complexes with the heavier Ae metals (Figures S31–S35). The mononuclear nature of **1**‐Mg and the dinuclear nature of the other compounds make further discussion of all oxidation potentials difficult.

The complexes **1**‐Ae (Ae=Mg, Ca, Sr, Ba) have been studied using the BP86‐D3(BJ)/def2‐SVP method. The most important calculated bond lengths and angles are in good agreement with experimental values (Table S9). Deviations for the Fe⋅⋅⋅Ae distances (Table 1) may partly be caused by the influence of the intermolecular forces on these comparatively weak interactions. NBO calculations (Table 2) show that the Fe and Ae atoms carry positive charges, which are very high for the Ae atoms, while N and O have large negative charges.

**Table 2:** Atomic partial charges *q* in **1**‐Ae complexes (BP86‐D3(BJ)/def2‐SVP).

complex	*q*(Ae)	*q*(N1)^[a]^	*q*(N2)^[a]^	*q*(Fe)	*q*(O)
**1**‐Mg	1.77	−1.06	−1.00	0.55	−0.70
**1**‐Ca	1.74	−0.85	−0.98	0.54	−0.69
**1**‐Sr	1.74	−0.83	−0.97	0.55	−0.69
**1**‐Ba	1.73	−0.82	−0.93	0.56	−0.65 (−0.66)^[b]^

[a] See Figure 1 for atom numbering. [b] Slightly different values for the O atoms are found.

**Figure 2:**
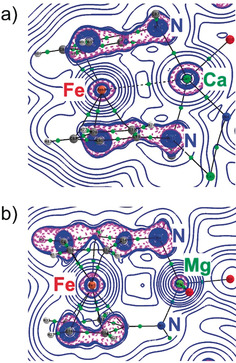
Contour plot of the Laplacian of the electron density, ∇^2^ *ρ*(**r**), in the Fe‐Ae‐N plane for a) **1**‐Ca and b) **1**‐Mg. The blue solid lines indicate regions of charge depletion (∇^2^ *ρ*(**r**)>0) and red dotted lines indicate regions of charge accumulation (∇^2^ *ρ*(**r**)<0). Small green circles represent bond critical points.

The electronic structures of the four complexes were analyzed with the QTAIM method.^24^ Figure S41 shows molecular graphs for all **1**‐Ae complexes containing bond critical points (bcp), ring critical points (rcp), and cage critical points (ccp). For the heavier metals (Ca, Sr, Ba), there is a bcp for the Fe⋅⋅⋅Ae interactions, while the Mg complex possesses a rcp in the Fe–Mg fragment but not a bcp. The shape of the Laplacian distribution ∇^2^ρ(**r**) for **1**‐Ca is shown in Figure 2 a. Corresponding Laplacian distributions for **1**‐Sr and **1**‐Ba look very similar (Figure S42). The QTAIM results clearly suggest covalent Fe⋅⋅⋅Ae bonding in **1**‐Ae (Ae=Ca, Sr, Ba) but not in **1**‐Mg (Figure 2 b).

Detailed insight into the nature of Fe⋅⋅⋅Ae bonding is provided by EDA‐NOCV^25^ calculations of **1**‐Ae. We calculated the adducts using a single Ae metal in different oxidation states (0, +1, +2) and the remaining fragment as interacting species in order to identify the best description of the electronic structures. This is indicated by the smallest energy change of the fragments during the formation of the chemical bond given by the Δ*E*_orb_ value.^32^ The most faithful representation comes from the Ae dications Ae^2+^ and the dianion as interacting species (Tables S10–S13). This agrees with the high positive charge of Ae atoms calculated by the NBO method (Table 2). The EDA calculations suggest that the polar bonds between Ae^2+^ and the remaining dianion have about two‐thirds electrostatic character while circa 30 % originates from orbital (covalent) interactions. The contribution of dispersion interactions is rather small, because one fragment is only a monoatomic metal. Further inspection of the covalent interactions using the EDA‐NOCV approach shows that the orbital term Δ*E*_orb_ possesses a large number of small pair interactions that provide the total covalent bonding (Table S14).

**Figure 3:**
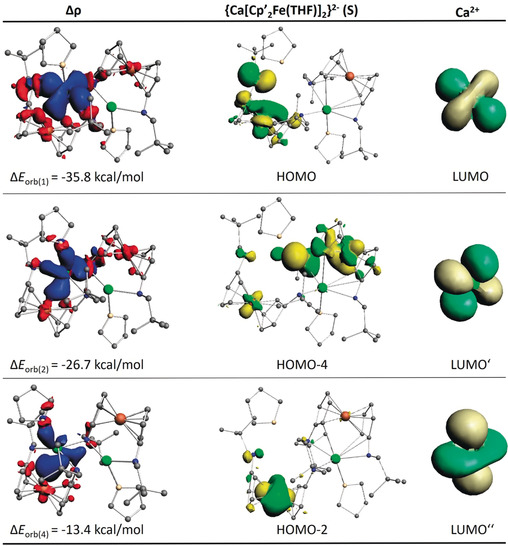
Shape of the deformation densities Δ*ρ*_(1),(2),(4)_ and the associated orbitals in **1**‐Ca of the pairwise orbital interactions Δ*E*_orb(1)_, Δ*E*_orb(2)_, and Δ*E*_orb(4)_.

Inspection of the orbitals that are involved in the pairwise interactions in complexes **1**‐Ae (Ae=Ca, Sr, Ba) reveals important information about the valence orbitals that are involved in the covalent bonds. Figure 3 shows the shape of the deformation densities Δ*ρ*_(1)_, Δ*ρ*_(2)_, and Δ*ρ*_(4)_ of **1**‐Ca and the associated most important orbitals of the interacting fragments, which come from the three relatively strong pairwise orbital interactions Δ*E*_orb(1)_, Δ*E*_orb(2)_, and Δ*E*_orb(4)_ of direct Fe⋅⋅⋅Ca interactions contributing 34 % to Δ*E*_orb_. The deformation densities of **1**‐Sr and **1**‐Ba are very similar to those of **1**‐Ca (Figures S43–S45). There are three contributions from Fe→Ca donation, all of which involve vacant d‐orbitals of Ca. The same result is obtained for **1**‐Sr and **1**‐Ba, but not for **1**‐Mg (Figure S46). There is only one comparatively weak pairwise orbital term Δ*E*_orb(5)_ coming from direct Fe⋅⋅⋅Mg interaction. Figure S46 shows the shape of the deformation density Δ*E*_orb(5)_ and the associated most important orbitals showing weak Fe→Mg donation into the vacant 3p‐orbital of Mg, which contributes only 6 % to the total covalent bonding of the Mg atom in **1**‐Mg. This explains the appearance of a bcp for the Fe⋅⋅⋅Ae interactions in the heavier Ae complexes while there is no Fe⋅⋅⋅Mg bcp.

The QTAIM parameters of *ρ*(**r**) at the bcp of the Fe⋅⋅⋅Ae interactions in **1**‐Ae (Ca, Sr, Ba) are listed in Table 1. The *ρ*(**r**) values are relatively small and the Laplacian of the electron density values, ∇^2^ *ρ*(**r**), are positive. More specific information comes from the energy value *H*(**r**) at the bcp. It has been shown before that negative values of *H*(**r**) indicate covalent bonding, which may sometimes exhibit positive Laplacian values, whereas positive or zero values of *H*(**r**) suggest electrostatic or van‐der‐Waals interactions.^33^ The QTAIM calculations thus agree with the EDA‐NOCV results that the Fe⋅⋅⋅Ae interactions in **1**‐Ae (Ca, Sr, Ba) are mainly electrostatic. The covalent part of the Fe⋅⋅⋅Ae interactions comes from Fe→Ae donation.

## Conclusion

Direct deprotonation of ligand **1**‐H_2_ led to ferrocene‐based chelate complexes **1**‐Ae (Ae=Mg, Ca, Sr, Ba), which crystallize as orange to intensely red products (the color deepens with metal size). Complex **1**‐Mg crystallized as a monomer and complexes with the larger metals as dimers in which empty coordination sites at the metals are filled by THF ligands. Complex **1**‐Mg shows a long Fe⋅⋅⋅Mg distance, but complexes with the larger Ae^2+^ cations measure short Fe⋅⋅⋅Ae distances that are similar to the sum of their covalent metal radii. These contacts increase linearly with the Ae^2+^ ionic radii and should be considered as bonding. Due to the similar ionic radii of Sr^2+^ and Eu^2+^, the Eu^II^ complex **1**‐Eu is isostructural with **1**‐Sr.

Further evidence for intramolecular Fe⋅⋅⋅Ae bonding is provided by a tilting of the Cp rings which increases with metal size. This causes the ferrocene HOMO frontier orbitals of mainly d‐character to bulge outwards, increasing the Fe donor capability. The energy needed for bending the ferrocene unit is compensated for by Fe→Ae bonding. Ring tilting is also evident from the ^1^H NMR chemical‐shift difference between the Cp α and β protons which increases with metal size.

While electrochemical studies are complicated by decomposition and formation of multimetallic dimers, the existence of an Fe→Ae bonding interaction in the intensely red complexes **1**‐Ca, **1**‐Sr and **1**‐Ba is supported by UV/Vis spectroscopy. An absorption of green light around 500 nm and very high extinction coefficients are typical for such dative bonding.

Most convincing evidence for Fe→Ae bonding was obtained by a comprehensive bonding analysis of **1**‐Ae complexes using the QTAIM and EDA‐NOCV methods. These studies are in favor of the conclusion that the heavier species **1**‐Ca, **1**‐Sr, and **1**‐Ba possess genuine Fe→Ae bonds which involve vacant d‐orbitals on the alkaline‐earth atoms and partially filled d‐orbitals on Fe. In contrast, there is only rather weak Fe→Mg donation into vacant Mg p‐orbitals in **1**‐Mg.

The crystal structures of **1**‐Ae (Ae=Mg, Ca, Sr, Ba) provide experimental evidence for the growing awareness that d‐orbitals on the heavier Ae metals Ca, Sr, and Ba can play an important role in bonding.

## Conflict of interest

The authors declare no conflict of interest.

## Supporting information

As a service to our authors and readers, this journal provides supporting information supplied by the authors. Such materials are peer reviewed and may be re‐organized for online delivery, but are not copy‐edited or typeset. Technical support issues arising from supporting information (other than missing files) should be addressed to the authors.

Supplementary
