# Supplementary material for: d–d Dative Bonding Between Iron and the Alkaline‐Earth Metals Calcium, Strontium, and Barium
Source: Angew Chem Int Ed Engl. 2020 Jul 2;59(34):14615–20. doi: 10.1002/anie.202005774 (PMC7496664; doi:10.1002/anie.202005774)
Supplement: Supplementary file 1 — Supplementary [file ANIE-59-14615-s001.pdf]

Angewandte Chemie
Eine Zeitschrift der Gesellschaft Deutscher Chemiker

Supporting Information

d–d Dative Bonding Between Iron and the Alkaline-Earth Metals Calcium, Strontium, and Barium

*Philipp Stegner, Christian Färber, Jan Oetzel, Ulrich Siemeling, Michael Wiesinger, Jens Langer, Sudip Pan, Nicole Holzmann, Gernot Frenking, Uta Albold, Biprajit Sarkar, and Sjoerd Harder**

anie_202005774_sm_miscellaneous_information.pdf

Supporting Information:

Contents

1. Supporting Experimental Data S1

1.1. General experimental procedures S1

1.2. Synthesis S1

1.3. NMR spectra of synthesized compounds S6

1.4 Single crystal X-ray diffraction S22

1.5 UV/Vis Spectra S36

1.6 Cyclic Voltammetry S41

2. Computational Details S45

3. References S83

1. Supporting Experimental Data

1.1. General Experimental Procedures

All experiments were conducted under an inert nitrogen atmosphere using standard Schlenk and glovebox techniques (MBraun, Labmaster SP). Toluene, *n*-hexane and benzene were degassed with nitrogen, dried over activated aluminium oxide (Solvent Purification System: Pure Solv 400−4−MD, Innovative Technology) and stored over 3Å molecular sieves under N_2. THF was refluxed over a sodium mirror, distilled under N_2 and stored over 3Å molecular sieves under N_2. Benzene-d_6 (99.6% D, Sigma Aldrich), THF-d_8 (99.6% D, Sigma Aldrich) and pyridine-d_5 (99.5% D, Sigma Aldrich) were dried over 3Å molecular sieves and stored under N_2. **1**-H_2 was synthesized starting from ferrocene-1,1-diamine according to the protocol of Siemeling *et al.*[S1] Ferrocene-1,1-diamine was synthesized starting from cyclopentadiene according to the protocol of Tamm *et al.*[S2] The dimeric amides $Ca[N(SiMe_3)_2]_2$[S3], $Sr[N(SiMe_3)_2]_2$[S3] and $Ba[N(SiMe_3)_2]_2$[S3] were synthesized according to literature procedures of Westerhausen. Mg(*n*Bu)$_2$ (1.0 M in heptane) was purchased by Sigma Aldrich and used without further purification. NMR spectra were recorded with a Bruker Avance III HD 400 MHz or a Bruker Avance III HD 600 MHz spectrometer. The spectra were referenced to the respective residual signals of the deuterated solvents. Elemental analysis was performed with an Hekatech Eurovector EA3000 analyzer.

1.2. Synthesis

Synthesis of 1-Mg:

1-H_2 (110 mg, 0.309 mmol) was dissolved in THF (3 mL) and cooled to -78°C. A solution of Mg(*n*Bu)$_2$ (0.280 mL, 1.0 M in heptane) was added drop wise via syringe and the reaction was slowly warmed to room temperature while the orange solution turned red. The red solution was stirred for 1 h at room temperature and filtered over celite. The solvent volume was reduced to approximately 0.5 mL under reduced pressure and *n*-hexane (2 mL) was added. A red-orange precipitate formed and THF (approximately 0.1 mL) was added until the precipitate almost completely redissolved. The slightly turbid

solution was filtered over celite and stored at -30°C for 2 days. Large red-orange plate shaped crystals formed and were isolated by decantation. The crystals were suitable for X-ray diffraction. Subsequent washing with *n*-hexane (2x 0.5 mL) and drying in vacuum gave 80 mg of red-orange crystals. The mother liquor was evaporated and the leftover solid was suspended in *n*-hexane (1 mL). THF (approximately 0.3 mL) was added until the precipitate almost completely redissolved. The suspension was filtered over celite. Prolonged storage at -30°C furnished a second batch of crystals. Decantation, washing with *n*-hexane (2x 0.5 mL) and drying in vacuum gave an additional 50 mg of red-orange crystals. Yield: 120 mg, 0.230 mmol 74 %. ^{1}H NMR (600 MHz, C_6D_6 + THF-d_8, 25°C): δ 3.96 (s, 2H, C*H*), 3.86 (s, 2H, C*H*), 3.56 (m, CH_2, β-THF), 3.04 (s, 2H, CH_2), 1.45 (m, CH_2, α-THF), 0.99 (s, 9H, CH_3) ppm. ^{13}C{^{1}H} APT NMR (151 MHz, C_6D_6 + THF-d_8, 25°C): δ 124.1 (*C*N), 67.9 (*C*H_2, β-THF), 67.1 (*C*H_2, β-THF-d_8), 66.2 (*C*H_2), 61.8 (*C*H), 60.4 (*C*H), 36.1 (*C*$(CH_3)_3$), 28.8 (*C*H_3), 25.8 (*C*H_2, α-THF) 24.8 (*C*H_2, α-THF-d_8), ppm. Anal. Calcd. for $C_{28}H_{46}FeMgN_2O_2$ (MW = 522.84 g/mol): C, 64.32; H, 8.87; N, 5.36. Found: C, 63.91; H, 8.62; N, 5.40.

Synthesis of 1-Ca:

A 10 mL glass ampoule was charged with a solution of $Ca[N(SiMe_3)_2]_2$ (55 mg, 0.152 mmol) in THF (4 mL) and a solution of 1-H_2 (50 mg, 0.140 mmol) in THF (4 mL) was added. Subsequently the ampoule was frozen in liquid nitrogen and vacuum sealed. After warming to room temperature the ampoule was placed in a pre-heated oil bath at 125°C for 2 days which led to conversion of the orange solution to a red solution. Subsequently the oil bath was slowly cooled to room temperature and large bright red crystals formed. To complete crystallization the ampoule was stored at room temperature for one week. The crystals were isolated by decantation, washed with THF (3x 0.5 mL) and dried under vacuum. 25 mg of bright red crystals suitable for X-ray diffraction were obtained. The mother liquor was dried under reduced pressure and the resulting powder was transferred in a 10 mL glass ampoule, suspended in THF (8 mL) and placed into a pre heated oil bath at 125°C until all solid had dissolved. Subsequently the oil bath was slowly cooled to room temperature and the ampoule was stored at room temperature for prolonged time (about

1 month) forming a second crop of crystals. Subsequently the crystals were isolated by decantation, washed with THF (3x 0.5 mL) and dried under vacuum. An additional 8 mg of bright red crystals were obtained. Yield: 32 mg, 0.069 mmol, 49 %. ^{1}H NMR (600 MHz, pyridine-d_5, 100°C): δ 3.98 (s, 2H, C*H*), 3.83 (s, 2H, C*H*), 3.66 (CH_2, β-THF), 3.14 (s, 2H, CH_2), 1.62 (CH_2, α–THF), 1.16 (s, 9H, CH_3) ppm. $^{13}C\{^{1}H\}$ APT NMR (151 MHz, Pyridine-d_5, 100°C): δ 112.0 (*C*N), 67.5 (*C*H_2, β-THF), 62.8 (*C*H), 59.7 (*C*H_2), 55.9 (*C*H), 31.0 (*C*(CH_3)$_3$), 27.4 (*C*H_3), 25.5 (*C*H_2, α-THF) ppm. Anal. Calcd. for $C_{48}H_{76}Ca_2Fe_2N_4O_2$ (MW = 933.00 g/mol): C, 61.79; H, 8.21; N, 6.01; Found: C, 61.57; H, 8.49; N, 6.09. Note: NMR spectra were recorded in pyridine at 100°C due to the poor solubility of the compound.

Synthesis of 1-Sr:

A 10 mL glass ampoule was charged with a solution of Sr[N($SiMe_3$)$_2$]$_2$ (100 mg, 0.245 mmol) in THF (4 mL) and a solution of 1-H_2 (70 mg, 0.196 mmol) in THF (4 mL) was added. Subsequently the ampoule was frozen in liquid nitrogen and vacuum sealed. After warming to room temperature the ampoule was placed in a pre-heated oil bath at 125°C for 1 day which led to formation of a red solution in which small red crystals started to form. Subsequently the oil bath was slowly cooled to room temperature and large bright red crystals formed. To complete crystallization the ampoule was stored at room temperature for one week. The crystals were isolated by decantation, washed with THF (3x 0.5 mL) and dried under vacuum. 60 mg of bright red crystals suitable for X-ray crystallography were obtained. The mother liquor was dried under reduced pressure and the resulting powder was transferred in a 10 mL glass ampoule, suspended in THF (8 mL) and placed into a pre heated oil bath at 125°C until all solid had dissolved. Subsequently the oil bath was slowly cooled to room temperature and the ampoule was stored at room temperature for prolonged time (about 2 weeks) forming a second crop of crystals. Subsequently the crystals were isolated by decantation, washed with THF (3x 0.5 mL) and dried under vacuum. An additional 10 mg of bright red crystals were obtained. Yield: 70 mg, 0.136 mmol, 69 %. ^{1}H NMR (600 MHz, Pyridine-d_5, 100°C): δ 4.11 (s, 2H, C*H*), 3.73 (s, 13H, CH_2, β-THF, + s, 2H, C*H*), 3.12 (s, 2H, CH_2), 1.74 (s, 13H, CH_2, α-THF), 1.18

(s, 9H, CH_3) ppm. $^{13}C\{^1H\}$ NMR (151 MHz, Pyridine-d_5, 100°C): δ 112.9 (*C*N), 68.3 (CH_2, β-THF), 63.7 (*C*H), 60.6 (CH_2), 56.8 (*C*H), 31.9 ($C(CH_3)_3$), 28.2 (CH_3), 26.3 (CH_2, α-THF) ppm. Anal. Calcd. for $C_{48}H_{76}Sr_2Fe_2N_4O_2$ (MW = 1028.10 g/mol): C, 56.08; H, 7.45; N, 5.45; Found: C, 55.63; H, 7.40; N, 5.79. Note: NMR spectra were recorded in pyridine at 100°C due to the poor solubility of the compound.

Synthesis of 1-Ba:

A 10 mL glass ampoule was charged with a solution of $Ba[N(SiMe_3)_2]_2(THF)_2$ (100 mg, 0.166 mmol) in THF (4 mL) and a solution of 1-H_2 (50 mg, 0.140 mmol) in THF (4 mL) was added. Subsequently the ampoule was frozen in liquid nitrogen and vacuum sealed. After warming to room temperature the ampoule was placed in a pre-heated oil bath at 125°C for 2 days which led to formation of a red solution in which small red crystals started to form. Subsequently the oil bath was slowly cooled to room temperature and large bright red crystals formed. To complete crystallization the ampoule was stored at room temperature for one week. The crystals were isolated by decantation, washed with THF (3x 0.5 mL) and dried under vacuum. 47 mg of bright red crystals suitable for X-ray crystallography were obtained. The mother liquor was dried under reduced pressure and the resulting powder was transferred in a 10 mL glass ampule, suspended in THF (8 mL) and placed into a pre heated oil bath at 125°C until all solid had dissolved. Subsequently the oil bath was slowly cooled to room temperature and the ampoule was stored at room temperature for prolonged time (about 2 months) forming a second crop of crystals. Subsequently the crystals were isolated by decantation, washed with THF (3x 0.5 mL) and dried under vacuum. An additional 11 mg of bright red crystals were obtained. Yield: 58 mg, 0.091 mmol, 65 %. ^1H NMR (600 MHz, pyridine-d_5, 100°C): δ 4.15 (s, 2H, C*H*), 3.75 (s, CH_2, β-THF), 3.60 (br s, 2H, C*H*), 2.99 (s, 2H, CH_2), 1.76 (s, CH_2, α-THF), 1.19 (s, 9H, CH_3) ppm. $^{13}C\{^1H\}$ NMR (151 MHz, pyridine-d_5, 100°C): δ 113.1 (*C*N), 68.3 (CH_2, β-THF), 67.5 (CH_2, β-THF-d_8), 65.8 (CH_2), 63.5 (CH_2) 51.2 (*C*H), 34.7 ($C(CH_3)_3$), 30.0 (CH_3), 26.3 (CH_2, α-THF) 25.3 (CH_2, α-THF-d_8) ppm. Anal. Calcd. for $C_{48}H_{76}Ba_2Fe_2N_4O_2$ (MW = 1271.72 g/mol): C, 52.89; H, 7.29; N, 4.41; Found: C, 52.58; H, 7.19, N, 4.46. Note: NMR spectra were recorded in pyridine at 100°C due to the poor solubility of the

compound. THF-d_8 was observed in the ^{13}C-NMR, which was found to be an impurity in the batch of pyridine-d_5 used for the ^{13}C-NMR sample.

Synthesis of 1-Eu:

1-H_2 (50 mg, 0.140 mmol) and $KN(SiMe_3)_2$ (56 mg, 0.281 mmol) were dissolved in THF (2 mL) and stirred over night at room temperature. The solvent was removed under reduced pressure and the resulting residue was dried until a free flowing red powder was obtained. Subsequently the crude 1-K_2 was redissolved in THF (10 mL), EuI_2 (68.2 mg, 0.168 mmol) was added and the reaction was stirred for 3 days. The precipitated KI was removed by centrifugation and filtration. The Eu complex is clearly better soluble than the corresponding Sr complex. Dark red crystals suitable for X-ray diffraction were grown from the resulting clear solution by slowly evaporating of the solvent. The crystals were isolated by decantation, washed with *n*-hexane (3x 0.5 mL) and dried in vacuum yielding 40 mg of red-orange crystals. Yield: 40 mg; 0.069 mmol 49 %. Anal. Calcd. for $C_{48}H_{76}Eu_2Fe_2N_4O_2$ (MW = 1156.78 g/mol): C, 49.84; H, 6.62; N, 4.84; Found: C, 49.54; H, 7.08; N, 4.52; Note: NMR spectra could not be recorded due to the paramagnetism of this compound.

1.3. NMR spectra of synthesized compounds

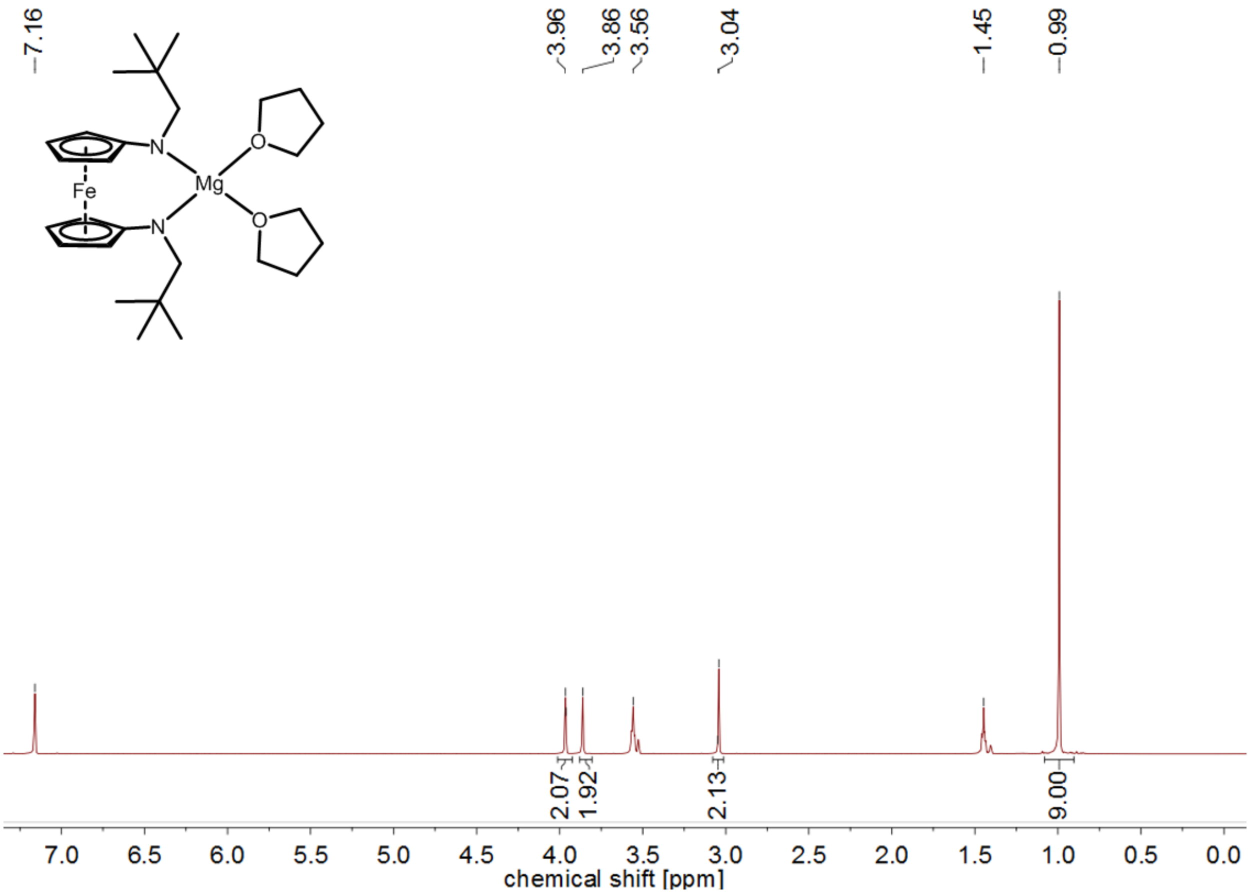

Figure S1: ^{1}H NMR spectrum of 1-Mg in C_6D_6 + THF-d_8 at 25°C.

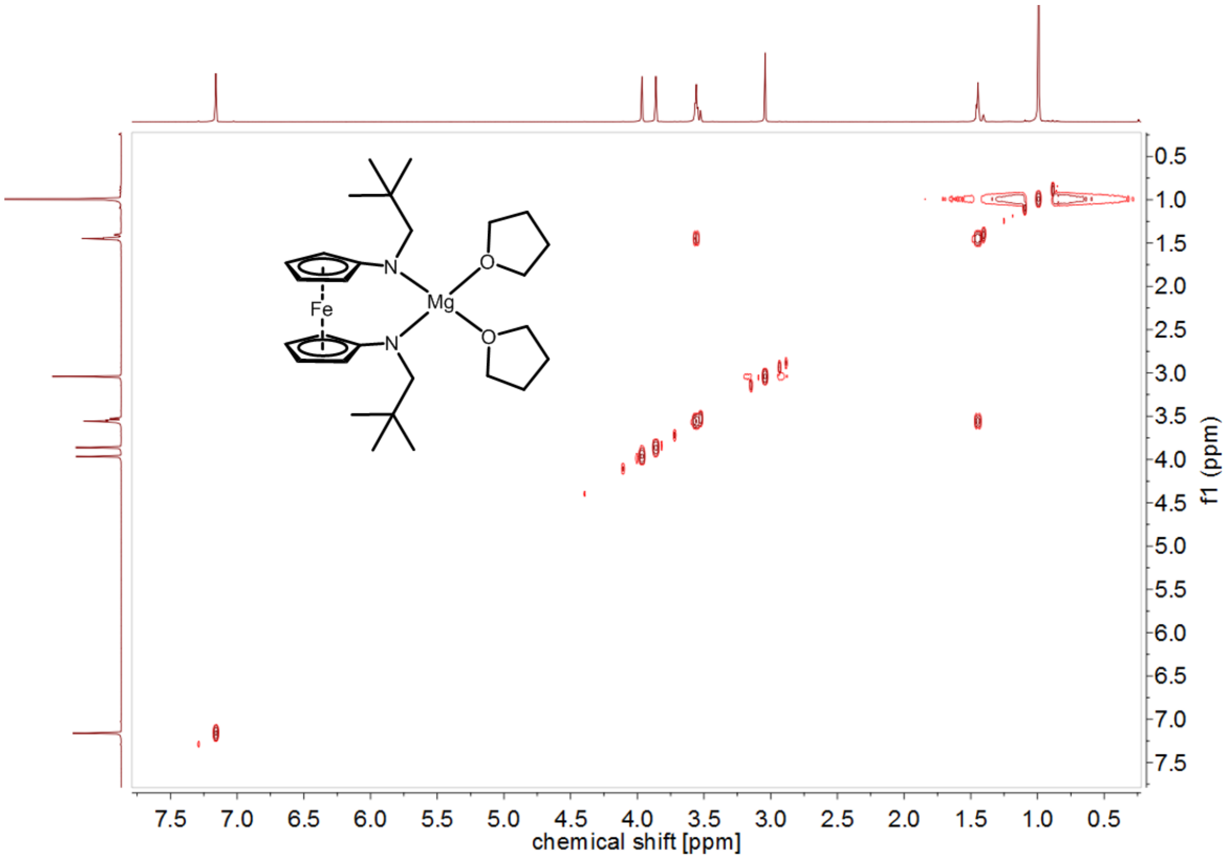

Figure S2: ^{1}H-^{1}H COSY spectrum of 1-Mg in C_6D_6 + THF-d_8 at 25°C.

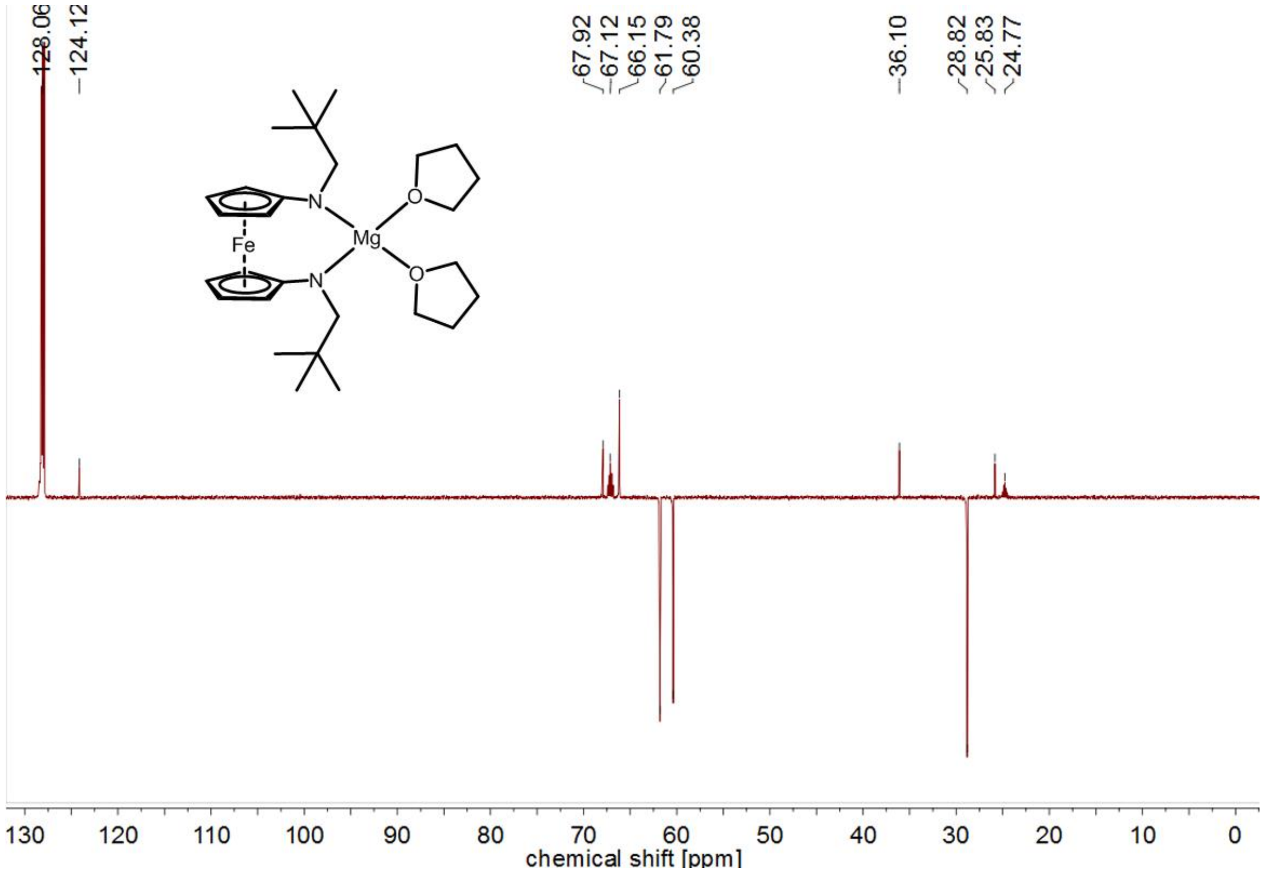

Figure S3: ^{13}C APT NMR spectrum of 1-Mg in C_6D_6 + THF-d_8 at 25°C.

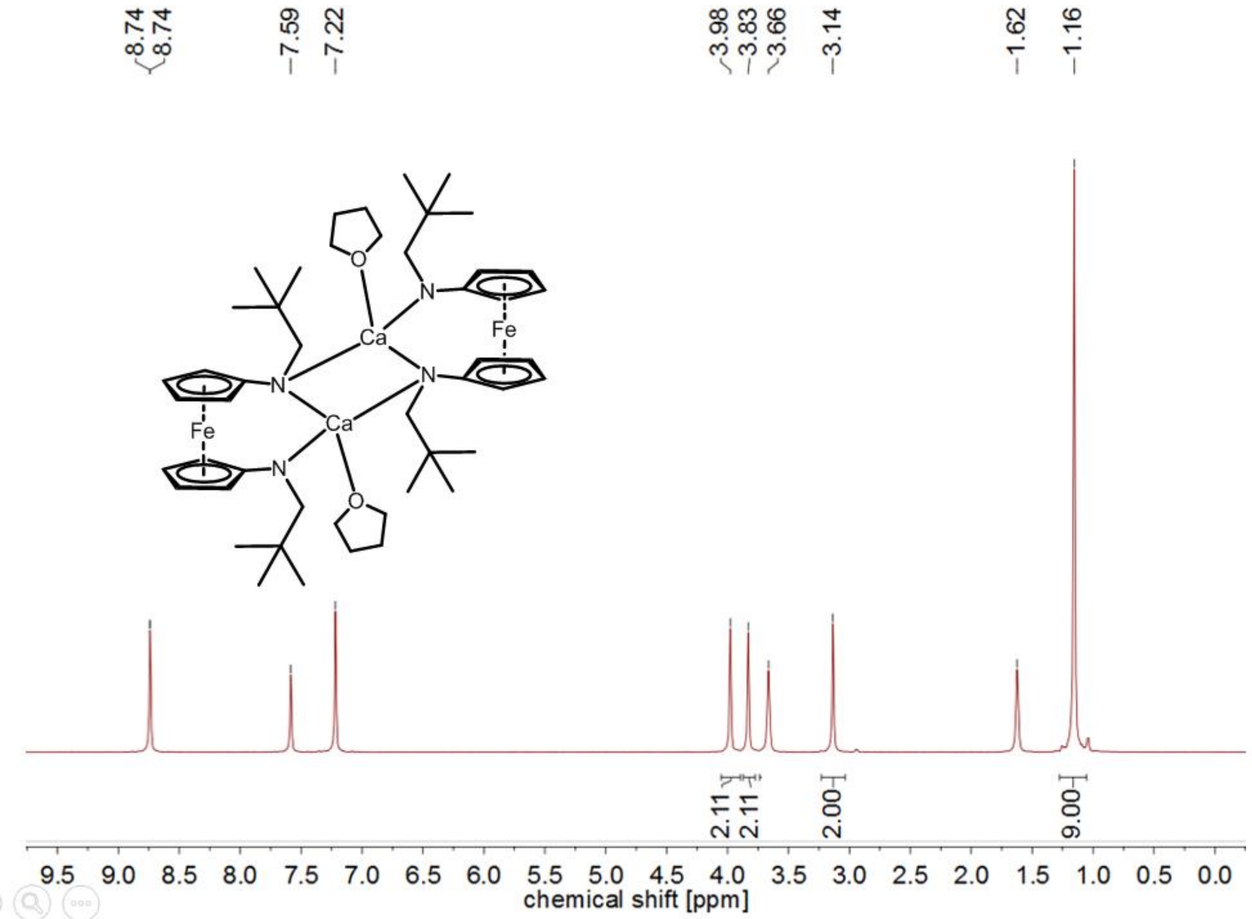

Figure S4: ^{1}H NMR spectrum of 1-Ca in pyridine-d_5 at 100°C.

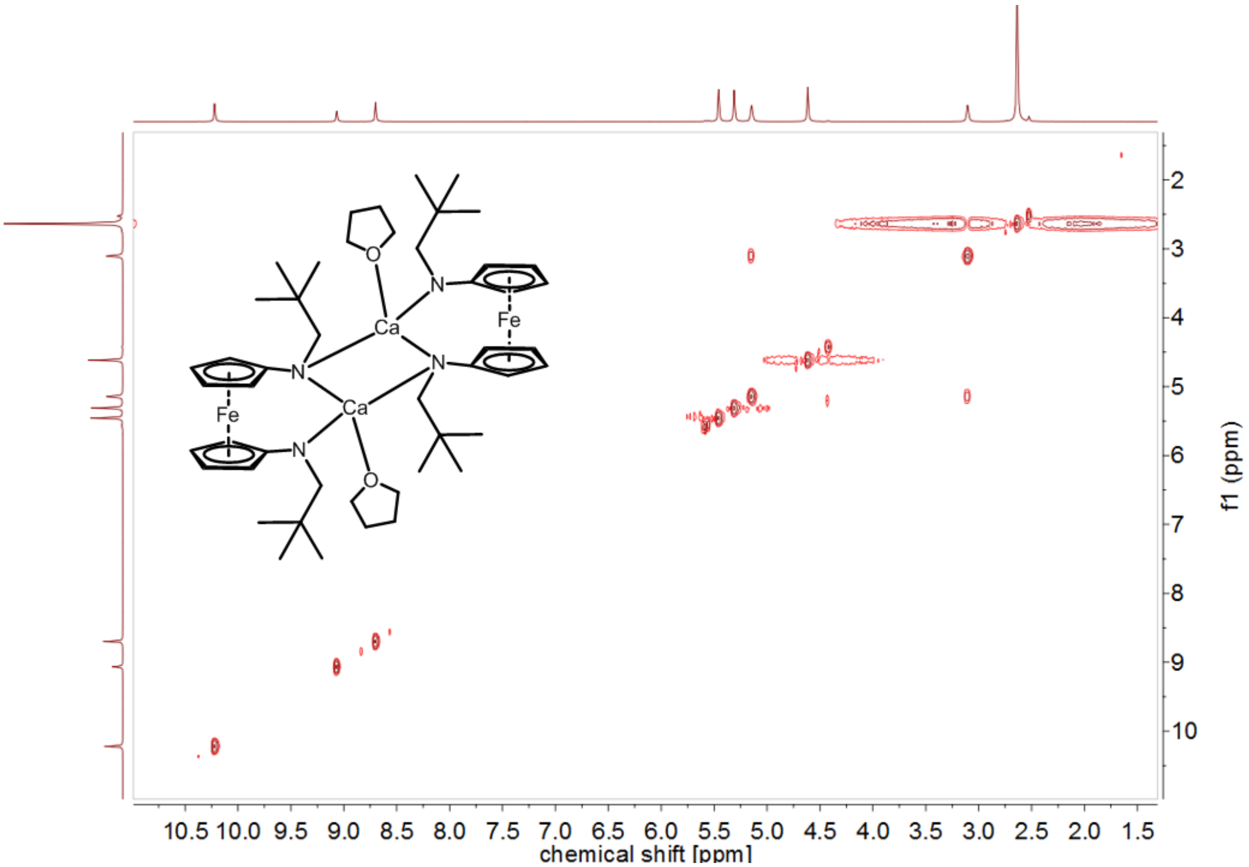

Figure S5: ^1H-^1H COSY spectrum of 1-Ca in pyridine-d_5 at 100°C.

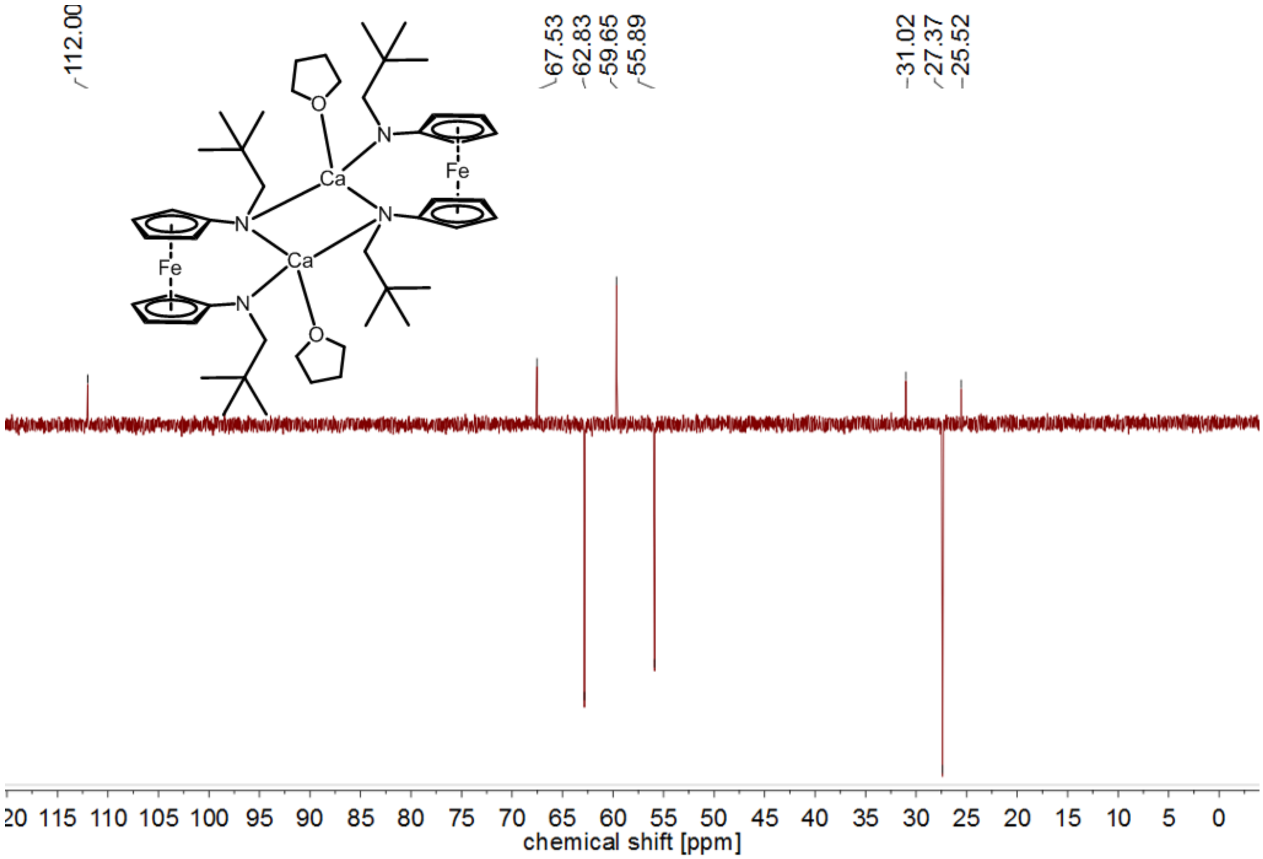

Figure S6: ^{13}C APT NMR spectrum of 1-Ca in pyridine-d_5 at 100°C.

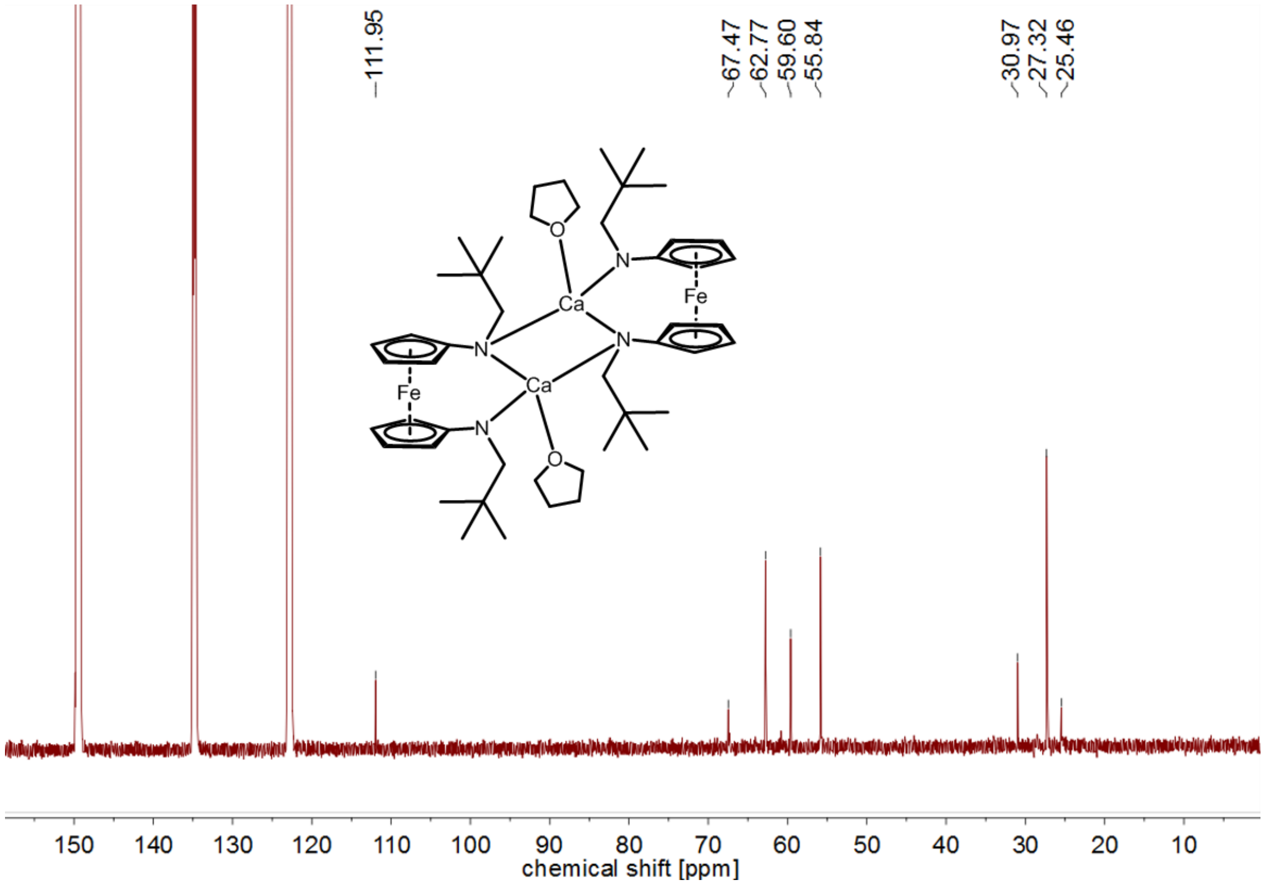

Figure S7: ^{13}C NMR spectrum of 1-Ca in pyridine-d_5 at 100°C.

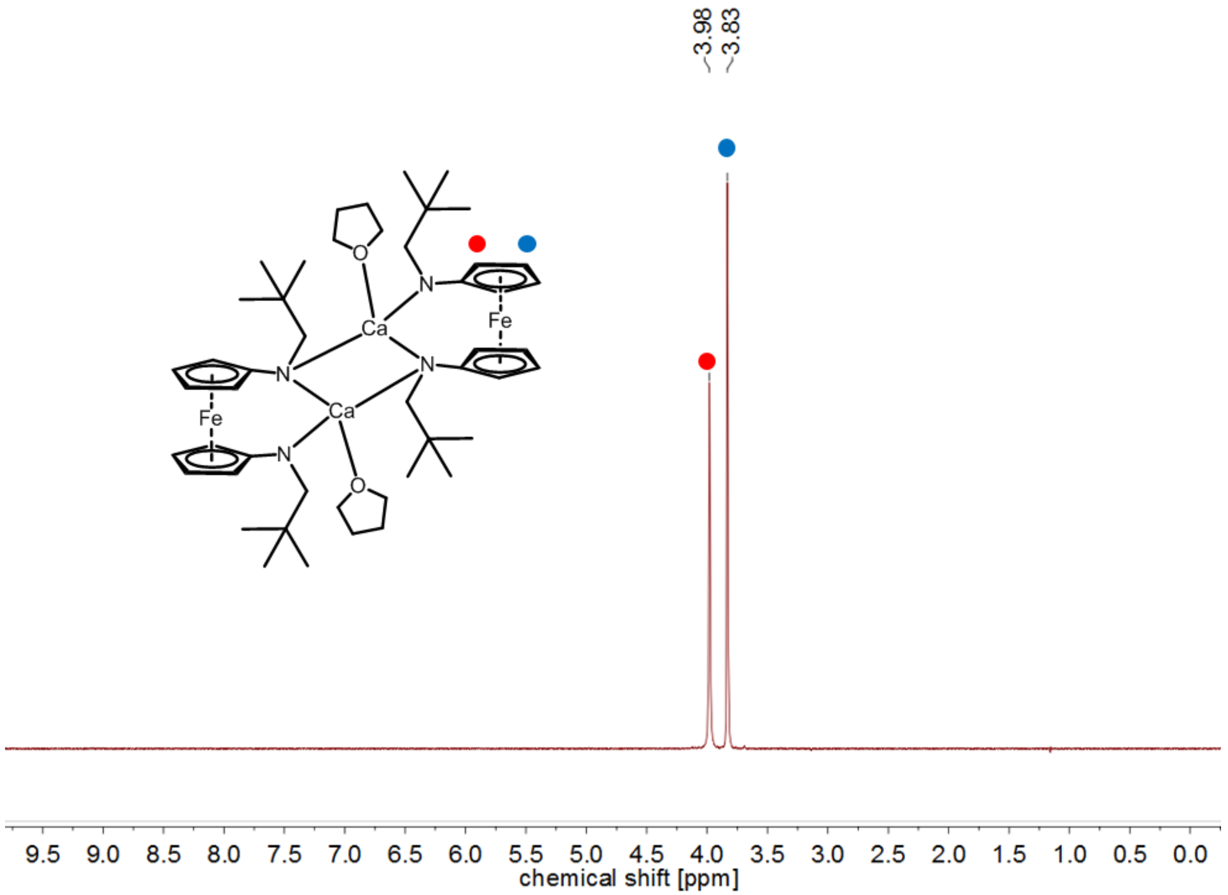

Figure S8: Selective TOCSY NMR spectrum of 1-Ca isolating the cyclopentadiene spin system in pyridine-d_5 at 60°C.

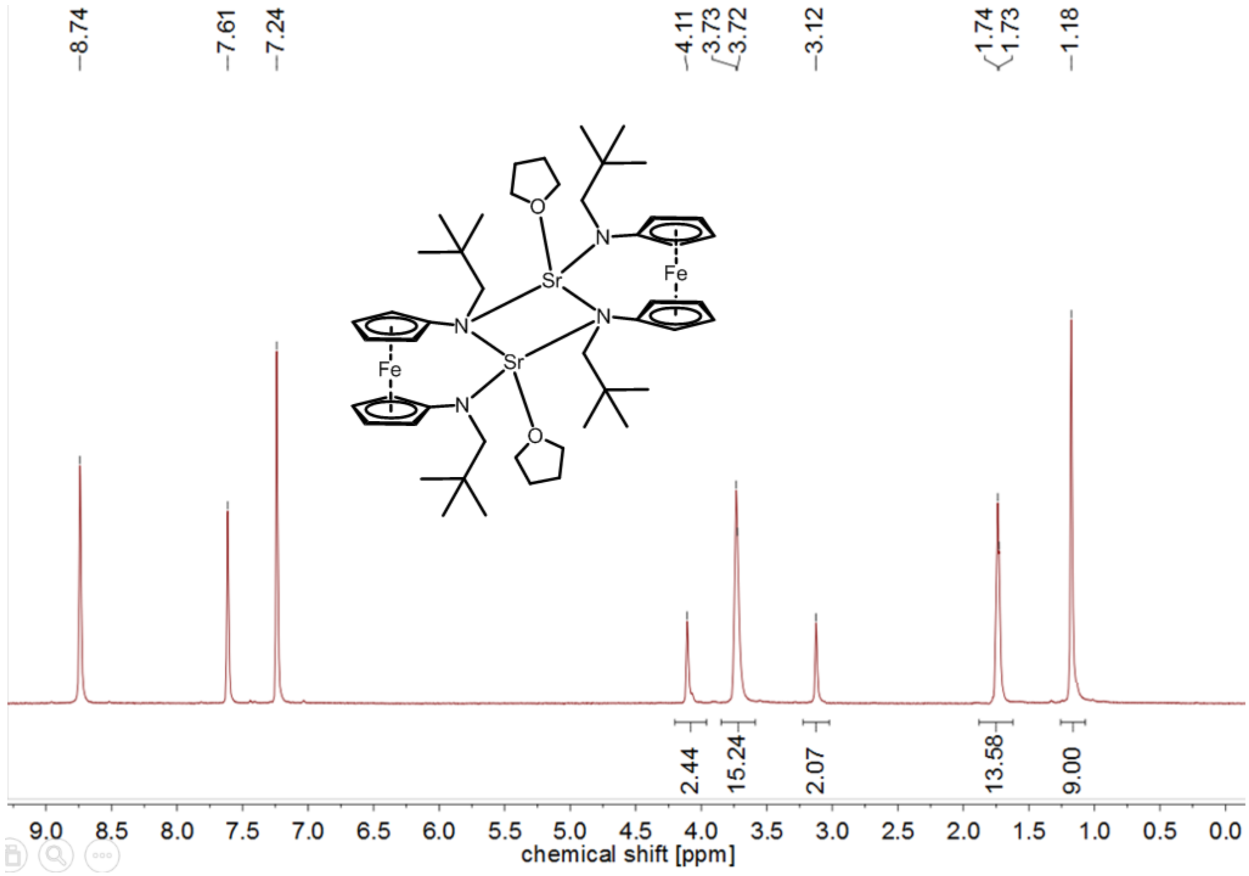

Figure S9: ^{1}H NMR spectrum of 1-Sr in pyridine-d_5 at 100°C.

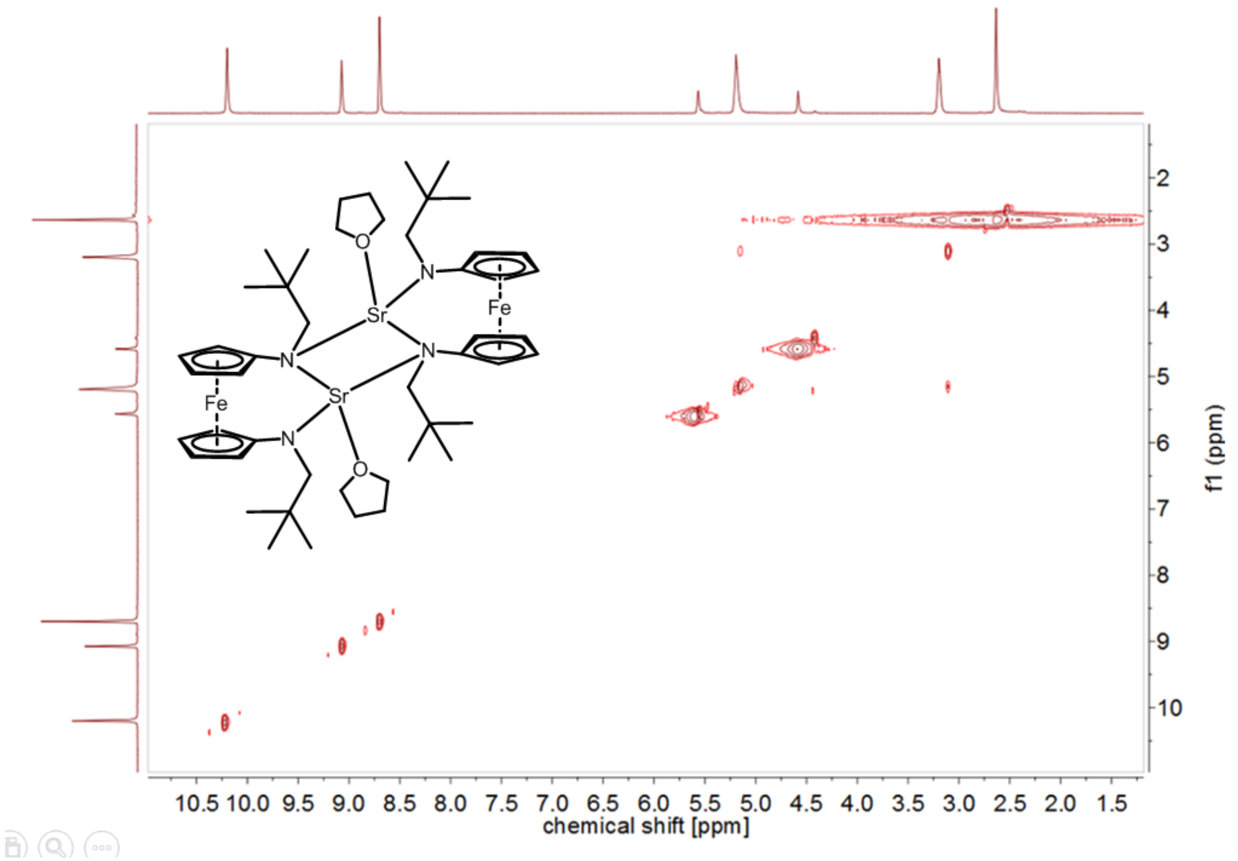

Figure S10: ^{1}H-^{1}H COSY spectrum of 1-Sr in pyridine-d_5 at 100°C.

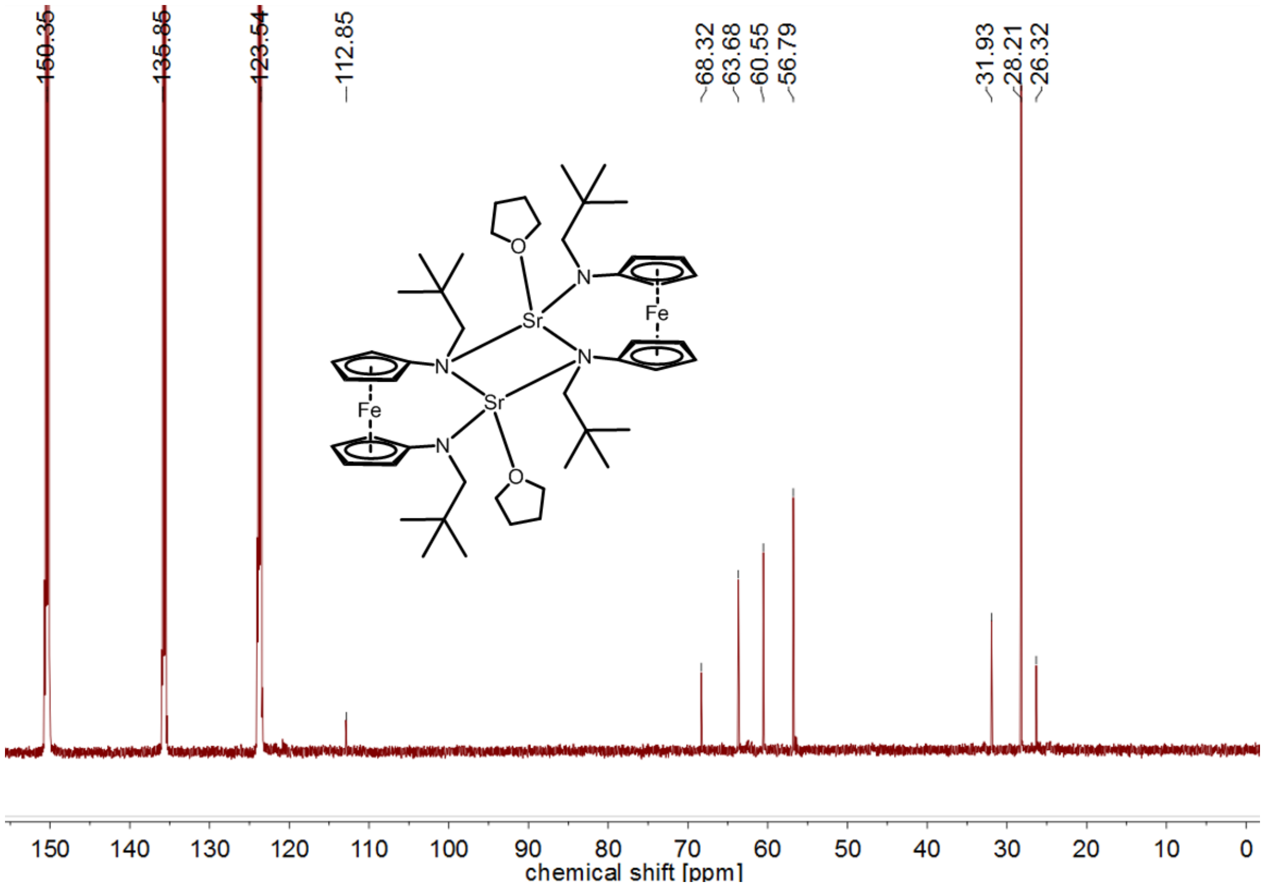

Figure S11: ^{13}C NMR spectrum of 1-Sr in pyridine-d_5 at 100°C.

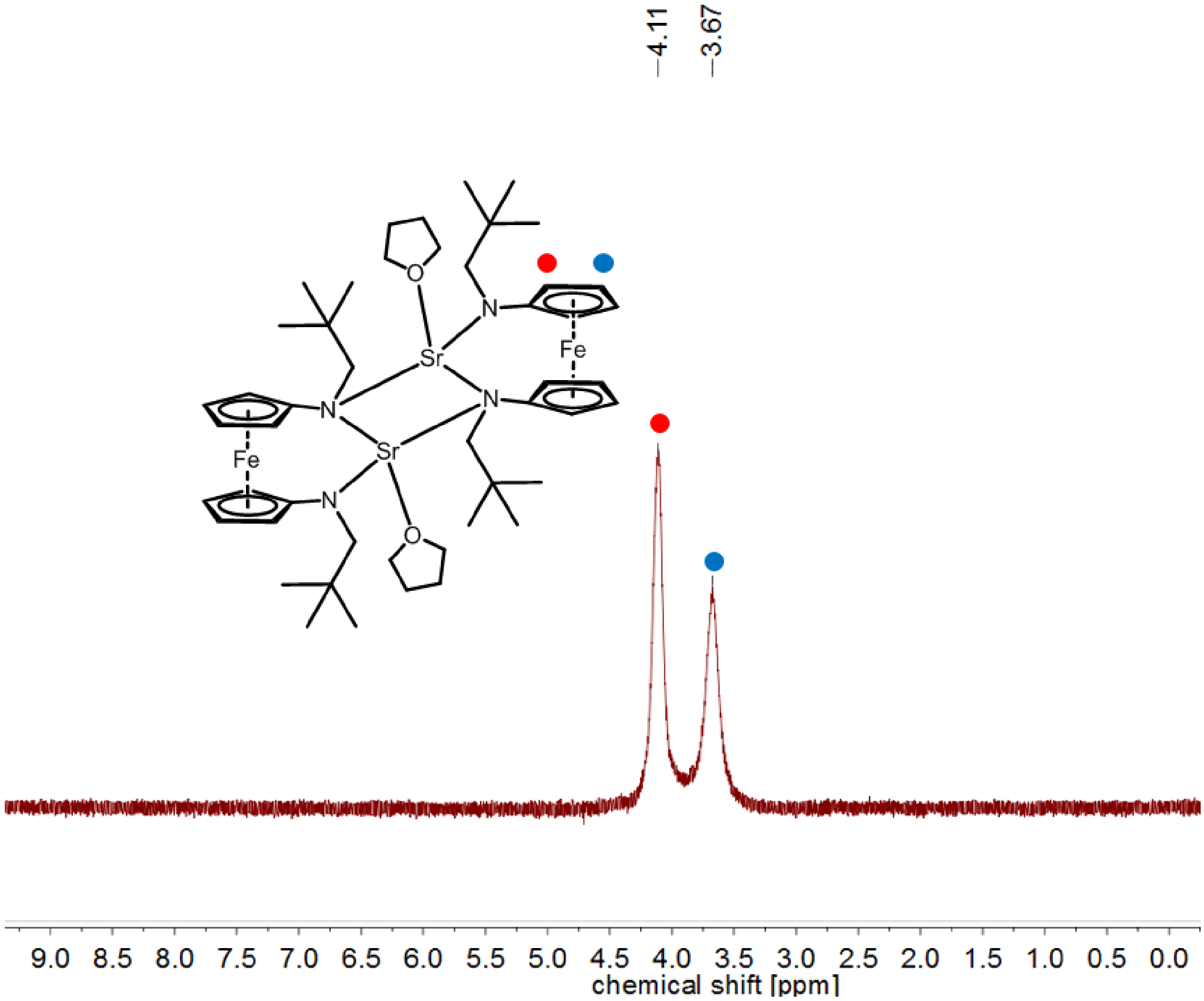

Figure S12: Selective TOCSY NMR spectrum of 1-Sr isolating the cyclopentadiene spin system in pyridine-d_5 at 80°C.

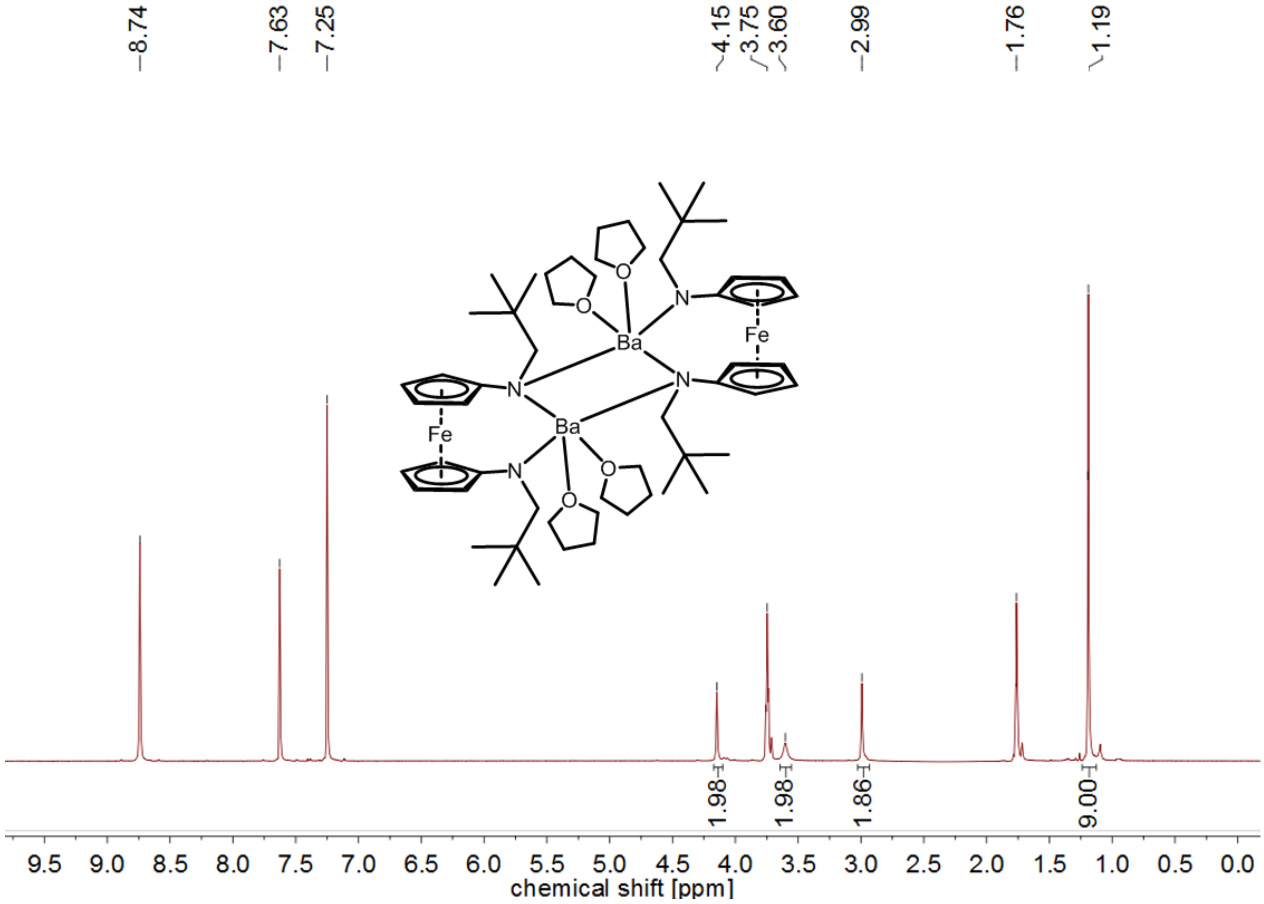

Figure S13: ^{1}H NMR spectrum of 1-Ba in pyridine-d_5 at 100°C.

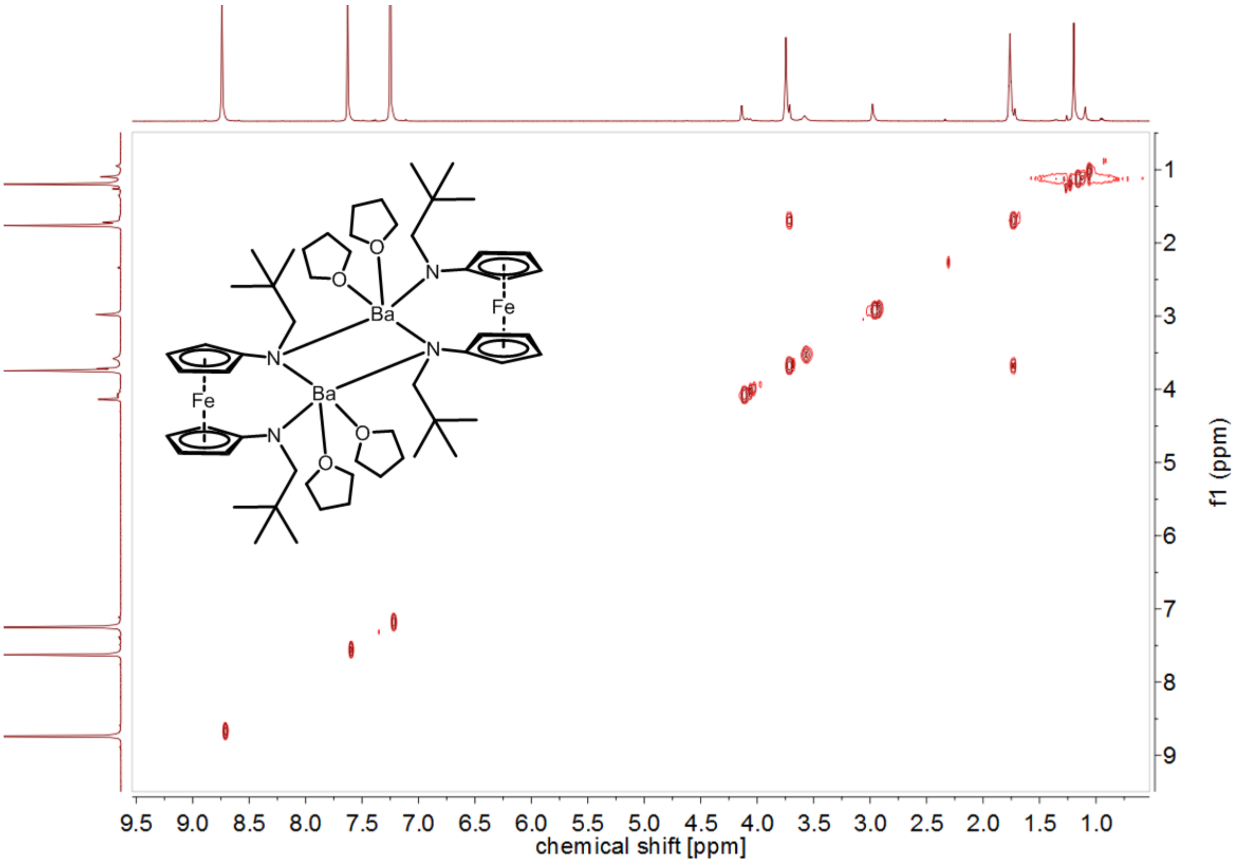

Figure S14: ^1H-^1H COSY spectrum of 1-Ba in pyridine-d_5 at 100°C.

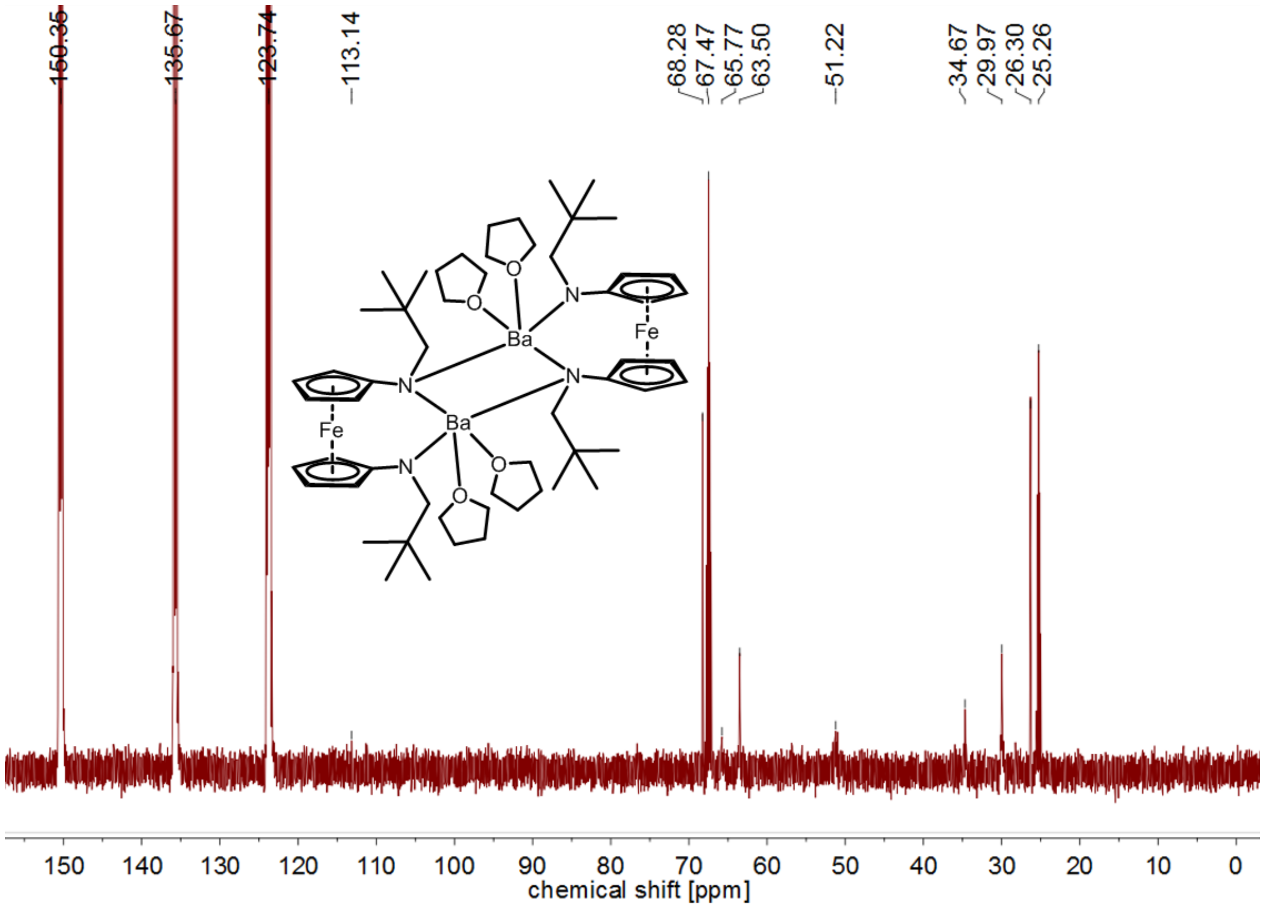

Figure S15: ^{13}C NMR spectrum of 1-Ba in pyridine-d_5 at 100°C.

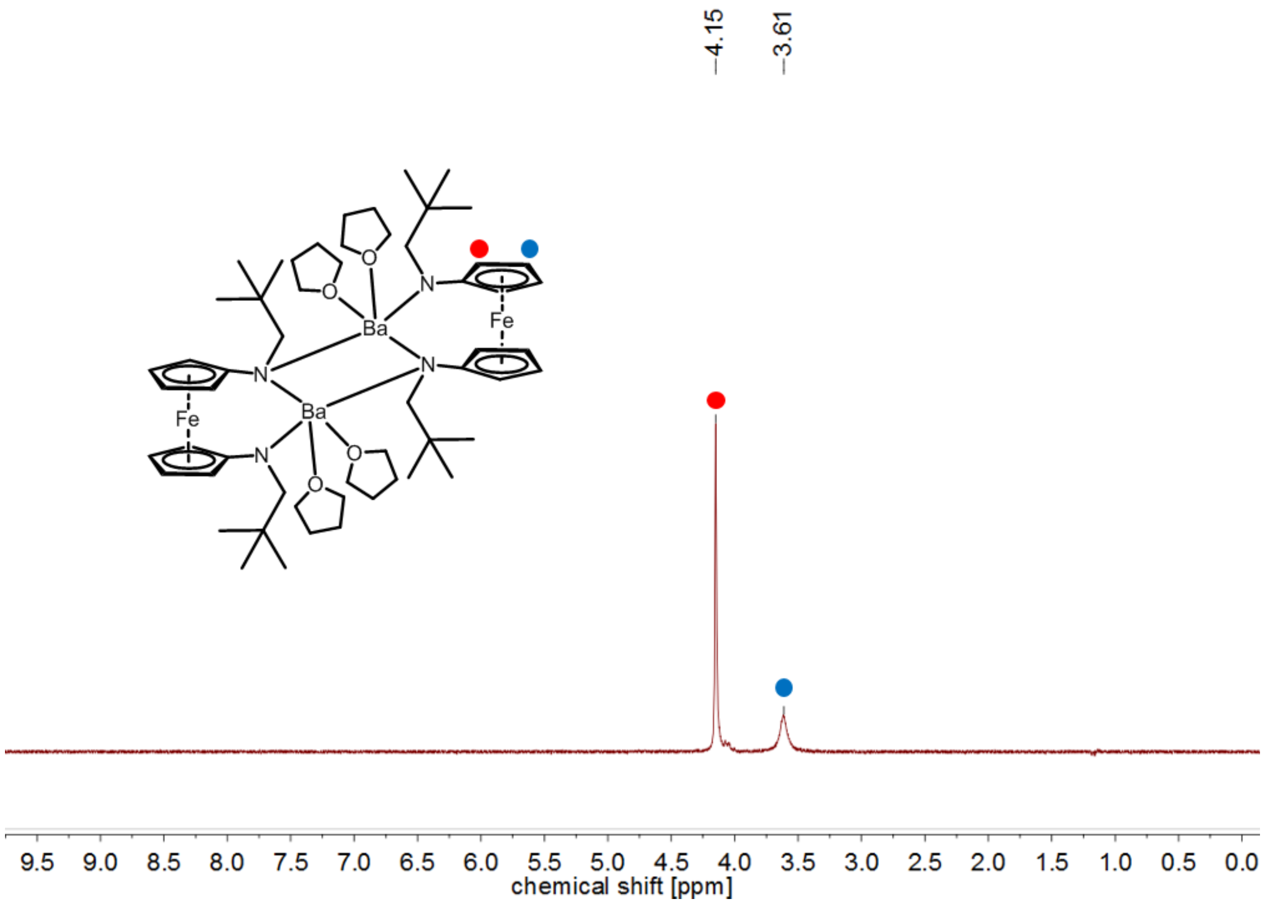

Figure S16: Selective TOCSY NMR spectrum of 1-Ba isolating the cyclopentadiene spin system in pyridine-d_5 at 100°C.

1.4 Single Crystal X-Ray Diffraction

General experimental information

Crystals were embedded in inert perfluoropolyalkyl ether (viscosity 1800 cSt; ABCR GmbH) and mounted using a Hampton Research CryoLoop. The crystal under investigation was then flash cooled to 100 K in a nitrogen gas stream and kept at this temperature during the experiment. The crystal structures were measured on a SuperNova Dual source diffractometer (Cu at home/near) with Atlas S2 detector using either a CuK*α* microfocus source (1-Mg, 1-Ca, 1-Sr) or a MoK*α* microfocus source (1-H_2, 1-Eu, 1-Ba). The measured data was processed with the CrysAlisPro software package.[S4] Using Olex2,[S5]the structure was solved with the ShelXT[S6]structure solution program using Intrinsic Phasing and refined with the ShelXL[S7]refinement package using Least Squares minimization. All non-hydrogen atoms were refined anisotropically. All hydrogen atoms (if not otherwise stated below) were placed in ideal positions and refined as riding atoms with relative isotropic displacement parameters.

Crystals of compound 1-H_2 were found to be twinned (racemic twinning) and in addition, the compound is heavily disordered. The fractional contributions of the two twin domains were refined to 0.51(2) and 0.49(2). The disorder was modeled with the help of similarity restraints (SADI) and rigid bond restraints (RIGU).[S8]. The relative occupancies of the two alternative orientations of the molecule were refined to 0.521(5) and 0.479(5). The positions of the amine hydrogen atoms were observed from difference Fourier maps and refined with restraints (SADI).

In case of 1-Ca, the hydrogen atoms H16A and H16B of the CH_2 group adjacent to N2 were observed from difference Fourier maps and refined. The same applies to 1-Sr.

The THF ligands of compound 1-Ba are disordered. The disorder was modeled with the help of similarity restraints (SIMU, SADI) and rigid bond restraints (RIGU).[S8] The relative occupancies of the two alternative orientations were refined to 0.870(4)/0.130(4) (THF1) and 0.612(14)/0.388(14) (THF2), respectively.

Crystallographic Data has been deposited with the Cambridge Crystallographic Data Centre as supplementary publication no. CCDC 1996024 for 1-H_2, CCDC 1996025 for 1-Mg, CCDC 1996026 for 1-Ca, CCDC 1996027 for 1-Sr, CCDC 1996028 for 1-Eu and CCDC 1996029 for 1-Ba. This data can be obtained free of charge from the Cambridge Crystallographic Data Centre via www.ccdc.cam.ac.uk/data_request/cif.

Table S1: Crystal data and structure refinement for 1-H_2.

**Identification code**	hasj190902a
**Empirical formula**	$C_{20}H_{32}FeN_2$
**Formula weight**	356.32
**Temperature/K**	100.0(2)
**Crystal system**	orthorhombic
**Space group**	$C222_1$
**a/Å**	6.96288(18)
**b/Å**	9.9016(2)
**c/Å**	27.3847(7)
**α/°**	90
**β/°**	90
**γ/°**	90
**Volume/Å^3**	1888.00(8)
**Z**	4
**ρ_{calc}g/cm^3**	1.254
**μ/mm^{-1}**	0.801
**F(000)**	768.0
**Crystal size/mm^3**	0.487 × 0.182 × 0.07
**Radiation**	Mo Kα (λ = 0.71073)
**2Θ range for data collection/°**	7.154 to 59.524
**Index ranges**	-9 ≤ h ≤ 9, -13 ≤ k ≤ 13, -38 ≤ l ≤ 37
**Reflections collected**	24928
**Independent reflections**	2528 [R_{int} = 0.0353, R_{sigma} = 0.0199]
**Data/restraints/parameters**	2528/83/218
**Goodness-of-fit on F^2**	1.133
**Final R indexes [I>=2σ (I)]**	R_1 = 0.0228, wR_2 = 0.0525
**Final R indexes [all data]**	R_1 = 0.0245, wR_2 = 0.0533
**Largest diff. peak/hole / e Å^{-3}**	0.28/-0.22

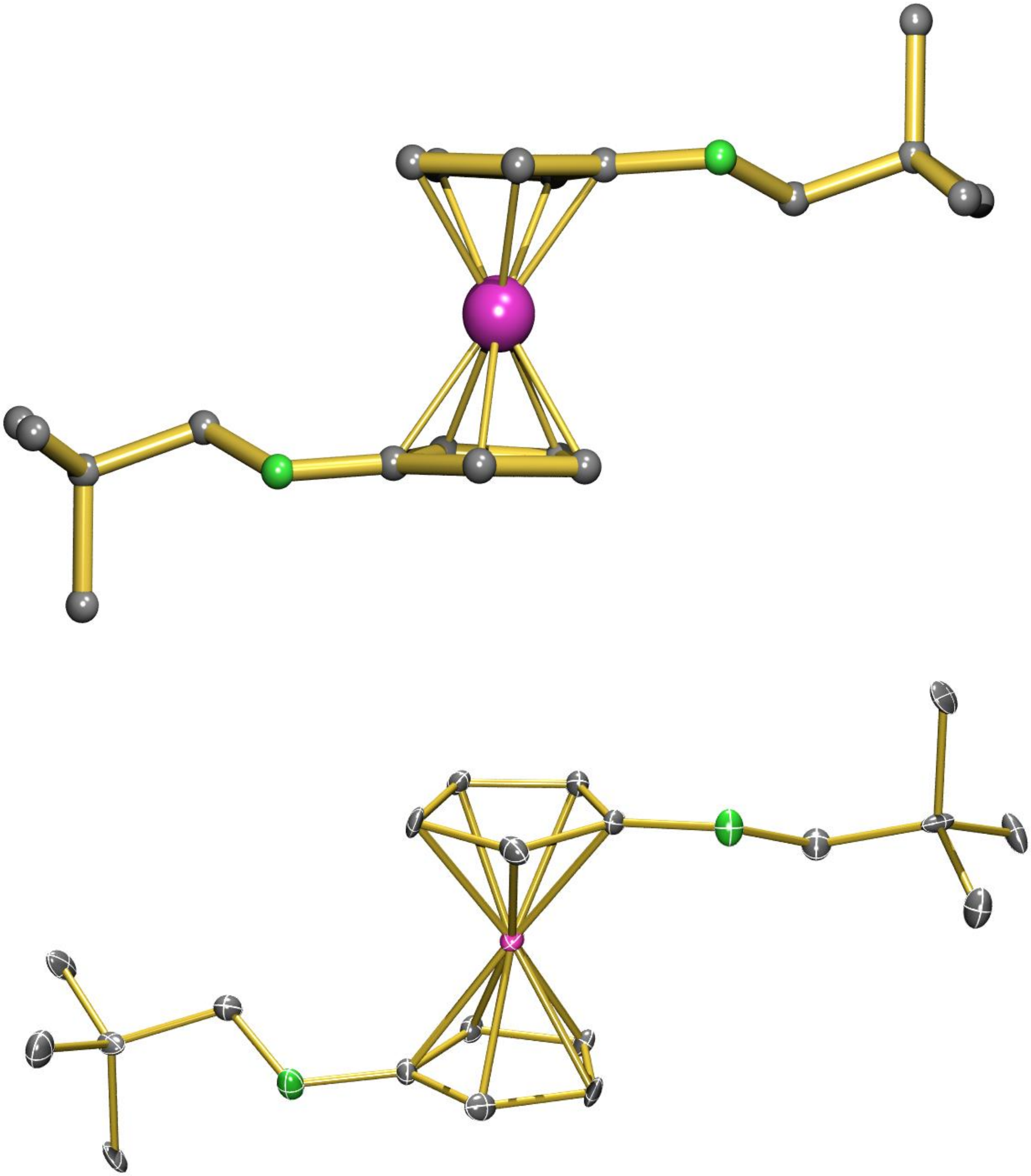

Figure S17: Crystal structure of 1-H_2 (top: ball-and-stick representation; bottom: ORTEP plot). Hydrogen atoms and disorder are omitted for clarity.

Table S2: Crystal data and structure refinement for 1-Mg.

**Identification code**	hasj181122a
**Empirical formula**	$C_{28}H_{46}FeMgN_2O_2$
**Formula weight**	522.83
**Temperature/K**	100.00(10)
**Crystal system**	monoclinic
**Space group**	$P2_1/n$
**a/Å**	11.31510(10)
**b/Å**	17.8811(2)
**c/Å**	13.8000(2)
**α/°**	90
**β/°**	98.2170(10)
**γ/°**	90
**Volume/Å^3**	2763.44(6)
**Z**	4
**ρ_{calc}g/cm^3**	1.257
**μ/mm^{-1}**	4.793
**F(000)**	1128.0
**Crystal size/mm^3**	0.1907 × 0.1246 × 0.0998
**Radiation**	CuKα (λ = 1.54184)
**2Θ range for data collection/°**	8.146 to 145.264
**Index ranges**	$-13 \le h \le 13$, $-21 \le k \le 21$, $-17 \le l \le 16$
**Reflections collected**	21206
**Independent reflections**	5375 [R_{int} = 0.0362, R_{sigma} = 0.0325]
**Data/restraints/parameters**	5375/0/314
**Goodness-of-fit on F^2**	1.040
**Final R indexes [I>=2σ (I)]**	R_1 = 0.0300, wR_2 = 0.0696
**Final R indexes [all data]**	R_1 = 0.0354, wR_2 = 0.0714
**Largest diff. peak/hole / e Å^{-3}**	0.31/-0.2

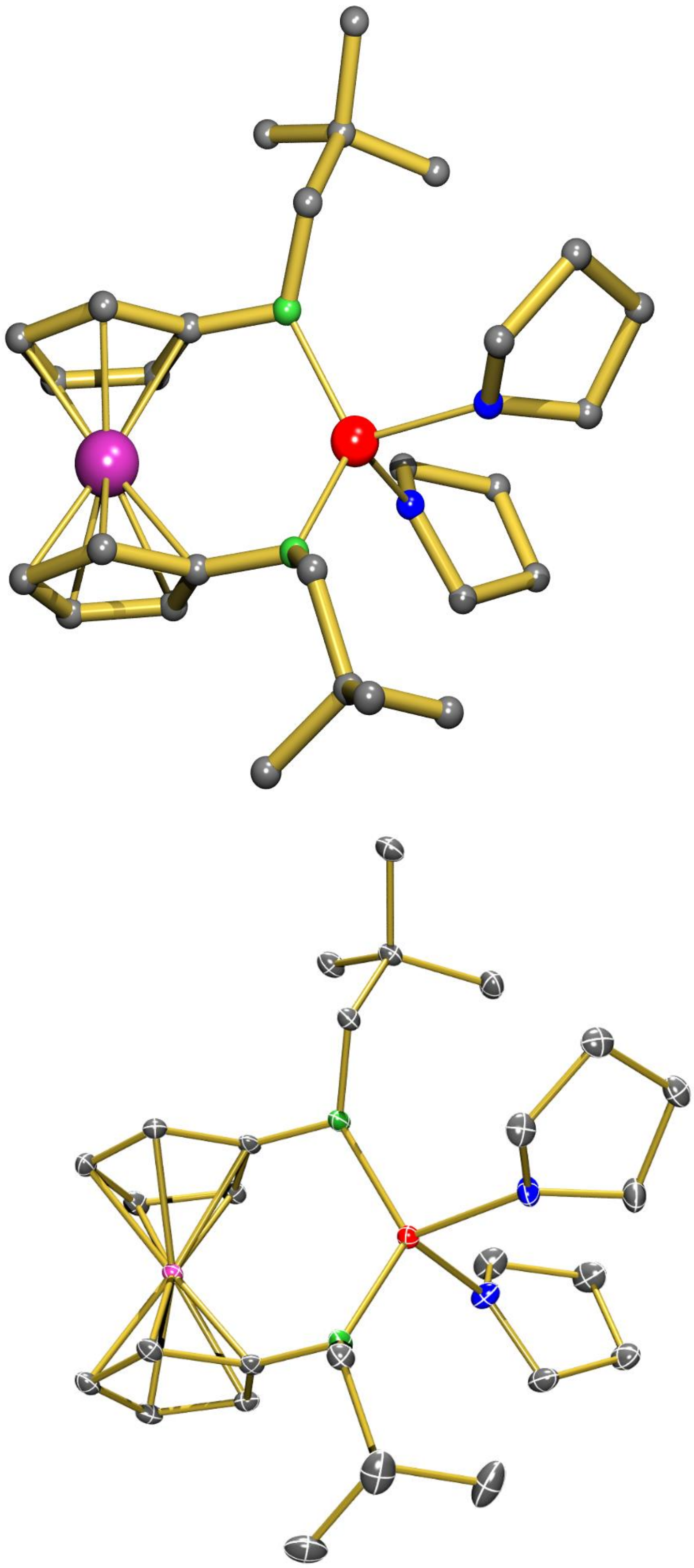

Figure S18: Crystal structure of 1-Mg (top: ball-and-stick representation; bottom: ORTEP plot). Hydrogen atoms are omitted for clarity.

Table S3: Crystal data and structure refinement for 1-Ca.

**Identification code**	hasj190219a
**Empirical formula**	$C_{48}H_{76}Ca_2Fe_2N_4O_2$
**Formula weight**	932.98
**Temperature/K**	100.01(10)
**Crystal system**	triclinic
**Space group**	P-1
**a/Å**	10.6520(8)
**b/Å**	11.7910(9)
**c/Å**	11.9476(7)
**α/°**	61.304(7)
**β/°**	65.091(6)
**γ/°**	69.034(7)
**Volume/Å^3**	1170.80(17)
**Z**	1
**ρ_{calc}g/cm^3**	1.323
**μ/mm^{-1}**	7.197
**F(000)**	500.0
**Crystal size/mm^3**	0.1677 × 0.0806 × 0.0376
**Radiation**	CuKα (λ = 1.54184)
**2Θ range for data collection/°**	8.718 to 145.16
**Index ranges**	-12 ≤ h ≤ 10, -14 ≤ k ≤ 14, -14 ≤ l ≤ 14
**Reflections collected**	13216
**Independent reflections**	4512 [R_{int} = 0.0366, R_{sigma} = 0.0395]
**Data/restraints/parameters**	4512/0/276
**Goodness-of-fit on F^2**	1.028
**Final R indexes [I>=2σ (I)]**	R_1 = 0.0351, wR_2 = 0.0854
**Final R indexes [all data]**	R_1 = 0.0399, wR_2 = 0.0886
**Largest diff. peak/hole / e Å^{-3}**	0.39/-0.40

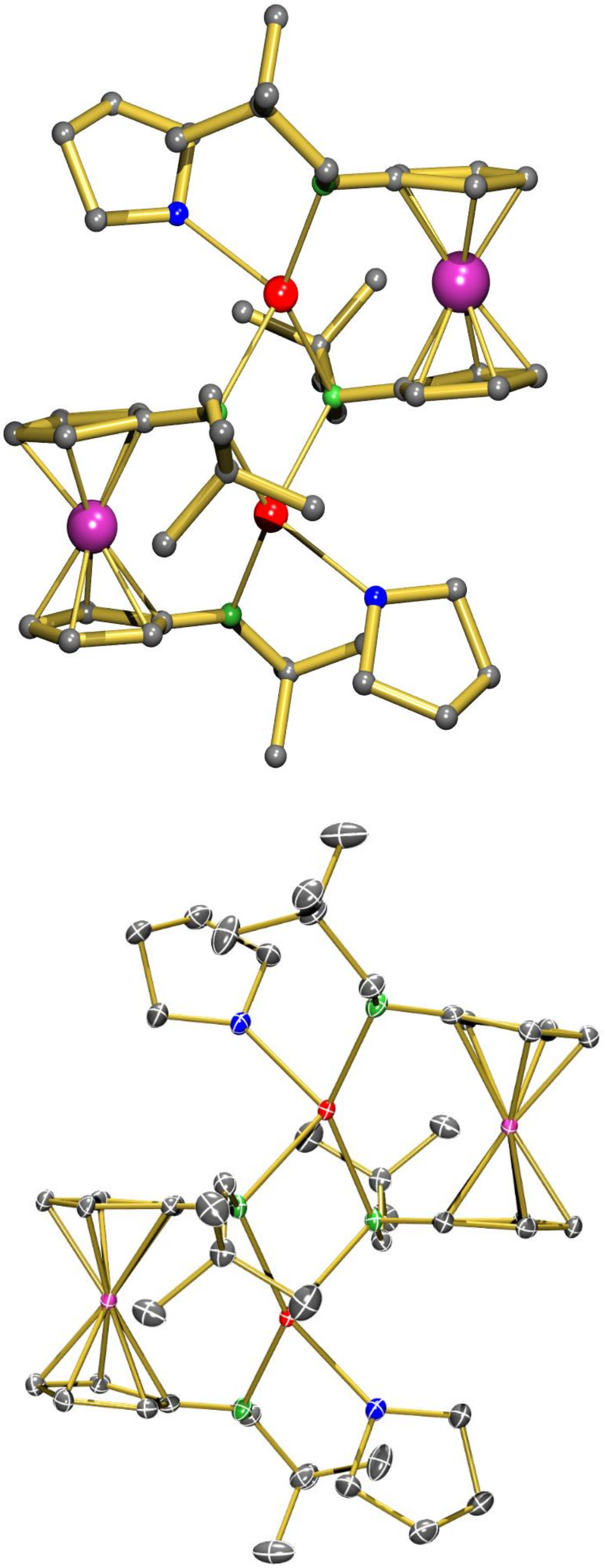

Figure S19: Crystal structure of 1-Ca (top: ball-and-stick representation; bottom: ORTEP plot). Hydrogen atoms are omitted for clarity.

Table S4: Crystal data and structure refinement for 1-Sr.

**Identification code**	hasj181105a
**Empirical formula**	$C_{24}H_{38}FeN_2OSr$
**Formula weight**	514.03
**Temperature/K**	100.01(10)
**Crystal system**	monoclinic
**Space group**	$P2_1/n$
**a/Å**	10.3443(2)
**b/Å**	24.0480(3)
**c/Å**	10.4400(2)
**α/°**	90
**β/°**	112.803(2)
**γ/°**	90
**Volume/Å^3**	2394.07(8)
**Z**	4
**ρ_{calc}g/cm^3**	1.426
**μ/mm^{-1}**	7.908
**F(000)**	1072.0
**Crystal size/mm^3**	0.2605 × 0.2017 × 0.0771
**Radiation**	CuKα (λ = 1.54184)
**2Θ range for data collection/°**	7.352 to 146.778
**Index ranges**	-12 ≤ h ≤ 12, -29 ≤ k ≤ 29, -12 ≤ l ≤ 12
**Reflections collected**	22575
**Independent reflections**	4715 [R_{int} = 0.0402, R_{sigma} = 0.0271]
**Data/restraints/parameters**	4715/0/277
**Goodness-of-fit on F^2**	1.044
**Final R indexes [I>=2σ (I)]**	R_1 = 0.0288, wR_2 = 0.0680
**Final R indexes [all data]**	R_1 = 0.0320, wR_2 = 0.0697
**Largest diff. peak/hole / e Å^{-3}**	0.47/-0.58

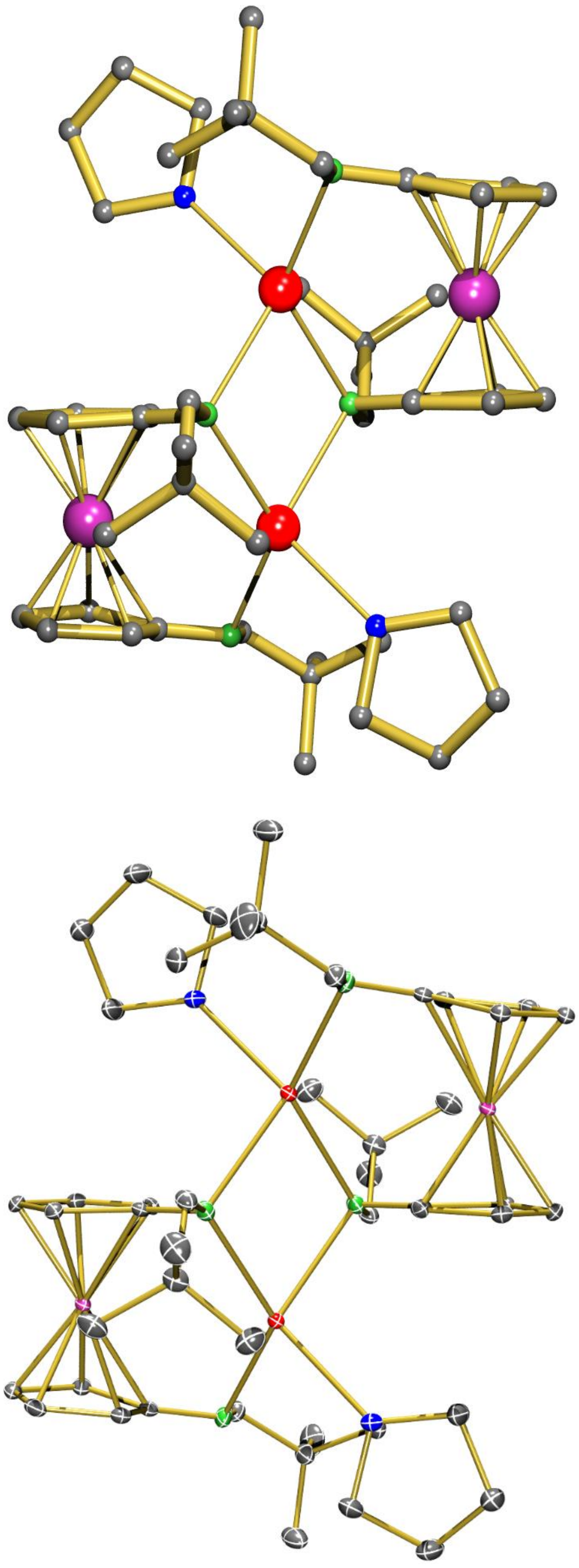

Figure S20: Crystal structure of 1-Sr (top: ball-and-stick representation; bottom: ORTEP plot). Hydrogen atoms are omitted for clarity.

Table S5: Crystal data and structure refinement for 1-Eu.

**Identification code**	hasj190104a
**Empirical formula**	$C_{48}H_{76}Eu_2Fe_2N_4O_2$
**Formula weight**	1156.74
**Temperature/K**	100.01(10)
**Crystal system**	monoclinic
**Space group**	$P2_1/n$
**a/Å**	10.3288(4)
**b/Å**	24.0285(6)
**c/Å**	10.4407(3)
**α/°**	90
**β/°**	112.444(4)
**γ/°**	90
**Volume/Å^3**	2394.95(14)
**Z**	2
**ρ_{calc}g/cm^3**	1.604
**μ/mm^{-1}**	3.217
**F(000)**	1172.0
**Crystal size/mm^3**	0.2952 × 0.2308 × 0.1781
**Radiation**	MoKα (λ = 0.71073)
**2Θ range for data collection/°**	5.812 to 59.052
**Index ranges**	-13 ≤ h ≤ 14, -30 ≤ k ≤ 32, -14 ≤ l ≤ 13
**Reflections collected**	21567
**Independent reflections**	5969 [R_{int} = 0.0334, R_{sigma} = 0.0368]
**Data/restraints/parameters**	5969/0/268
**Goodness-of-fit on F^2**	1.187
**Final R indexes [I>=2σ (I)]**	R_1 = 0.0326, wR_2 = 0.0568
**Final R indexes [all data]**	R_1 = 0.0383, wR_2 = 0.0584
**Largest diff. peak/hole / e Å^{-3}**	1.14/-0.74

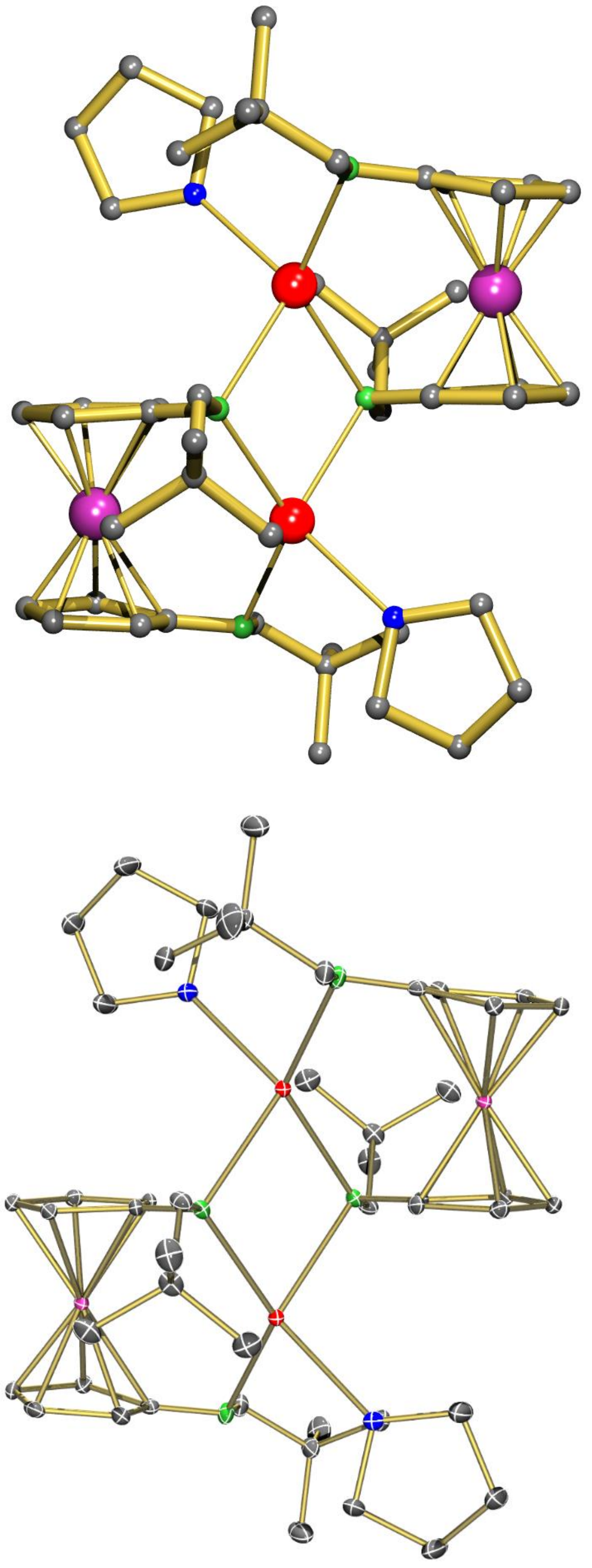

Figure S21: Crystal structure of 1-Eu (top: ball-and-stick representation; bottom: ORTEP plot). Hydrogen atoms are omitted for clarity.

Table S6: Crystal data and structure refinement for 1-Ba.

**Identification code**	hasj180926b
**Empirical formula**	$C_{56}H_{90}Ba_2Fe_2N_4O_4$
**Formula weight**	1269.69
**Temperature/K**	100.01(10)
**Crystal system**	monoclinic
**Space group**	$P2_1/c$
**a/Å**	10.2766(2)
**b/Å**	15.8347(3)
**c/Å**	17.8173(4)
**α/°**	90
**β/°**	101.849(2)
**γ/°**	90
**Volume/Å^3**	2837.57(10)
**Z**	2
**ρ_{calc}g/cm^3**	1.486
**μ/mm^{-1}**	1.916
**F(000)**	1300.0
**Crystal size/mm^3**	0.1108 × 0.086 × 0.0424
**Radiation**	MoKα (λ = 0.71073)
**2Θ range for data collection/°**	6.09 to 59.416
**Index ranges**	-13 ≤ h ≤ 14, -21 ≤ k ≤ 21, -23 ≤ l ≤ 24
**Reflections collected**	30009
**Independent reflections**	7098 [R_{int} = 0.0393, R_{sigma} = 0.0401]
**Data/restraints/parameters**	7098/60/354
**Goodness-of-fit on F^2**	1.037
**Final R indexes [I>=2σ (I)]**	R_1 = 0.0329, wR_2 = 0.0587
**Final R indexes [all data]**	R_1 = 0.0474, wR_2 = 0.0632
**Largest diff. peak/hole / e Å^{-3}**	1.51/-0.45

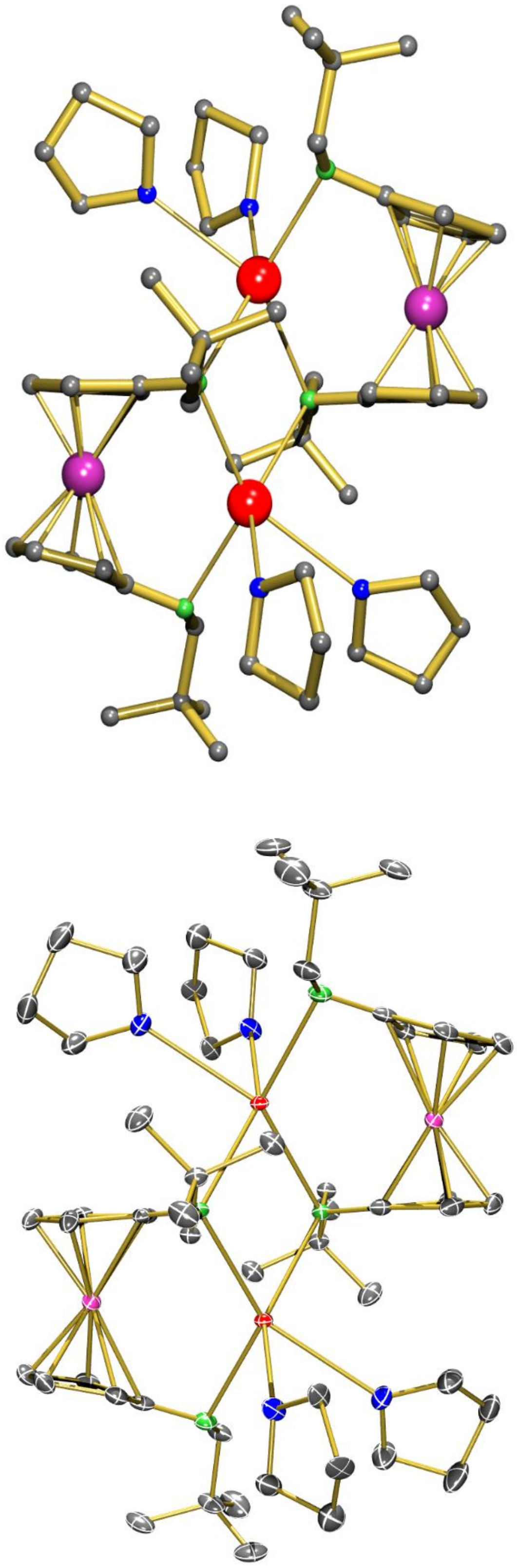

Figure S22: Crystal structure of 1-Ba (top: ball-and-stick representation; bottom: ORTEP plot). Hydrogen atoms and disorder are omitted for clarity.

1.5 UV/Vis Spectra

General Information

All UV/Vis spectra were recorded under rigorous exclusion of moisture and oxygen in screw sealed, gas tight, glass cuvettes, using an Agilent Technologies Cary 60 UV-vis spectrometer. All samples were prepared in a glovebox. The cuvettes were treated with *t*-BuLi (1.7M in pentane) and washed with *n*-pentane prior to usage. All compounds were measured in freshly distilled and thoroughly dried pyridine. (Sigma Aldrich, ACS reagent grade, distillation over freshly ground CaH_2, additional drying over activated MS 3Å). All complexes are intensely colored. Due to the very high air and moisture sensitivity of the compounds and unavoidable traces of water in the pyridine (< 10 ppm) a dilution to the level necessary for applying Lambert-Beer-Law (absorbance below 1.0 a.u. with respect to the first peak in the UV-region) could not be achieved without considerable complex hydrolysis. The UV/vis spectra were recorded at the concentrations of given in Table S7. Since we could not dilute these solutions any further due to hydrolysis, only a rough estimation of the extinction coefficients is given. The characteristic absorption for complexes with a κ^3-*bis*(donor)ferrocenyl-metal bonding mode[S9] is located around 500 nm. All spectra were recorded at room temperature and in all cases a background spectrum was measured first and subtracted from the spectrum of the compound of interest. The measured extinction coefficients are summarized in table S7.

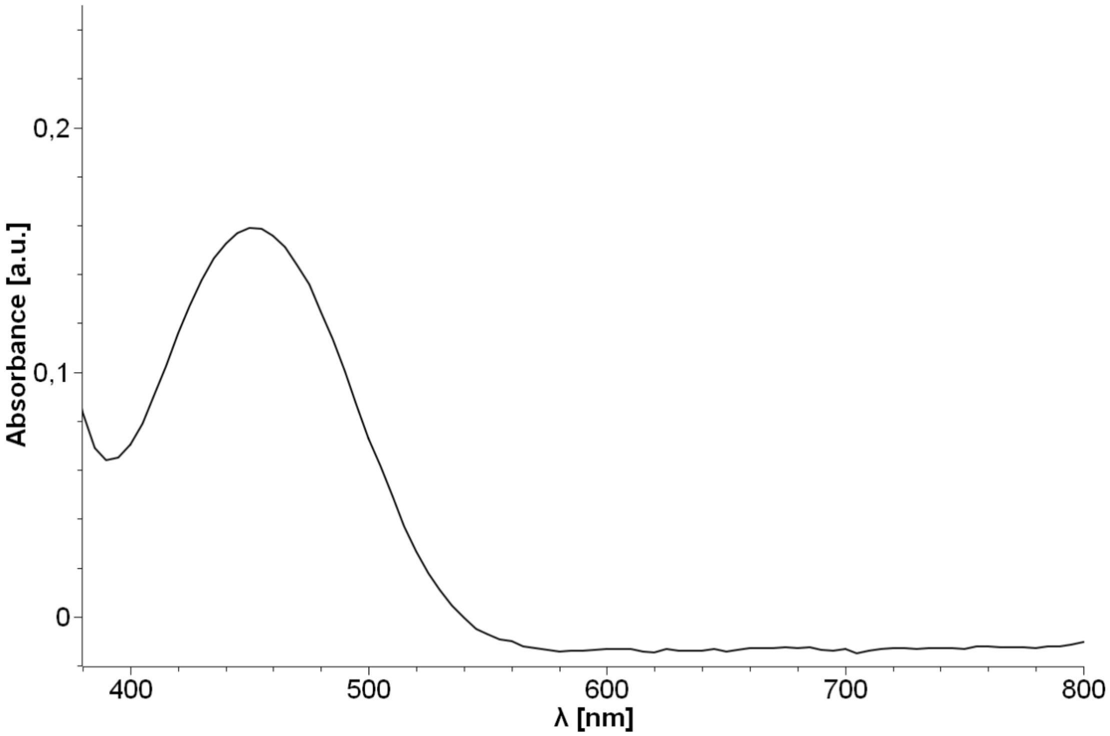

Figure S23: UV/Vis of 1-H_2.

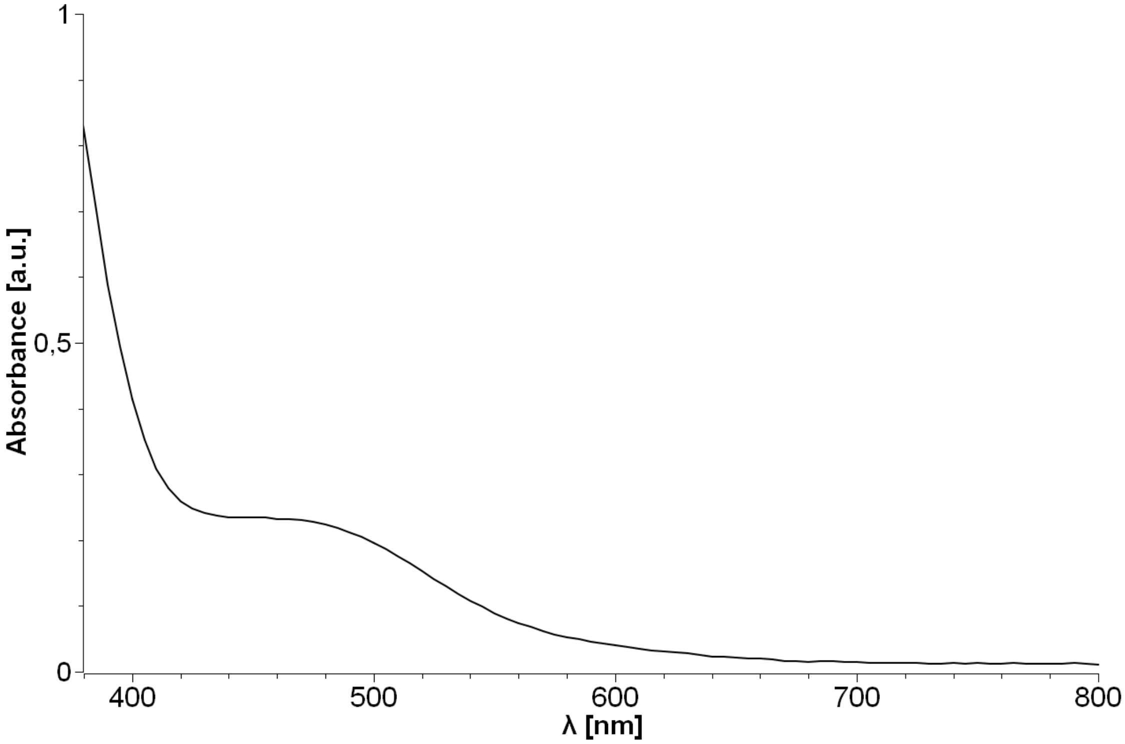

Figure S24: UV/Vis of 1-Mg.

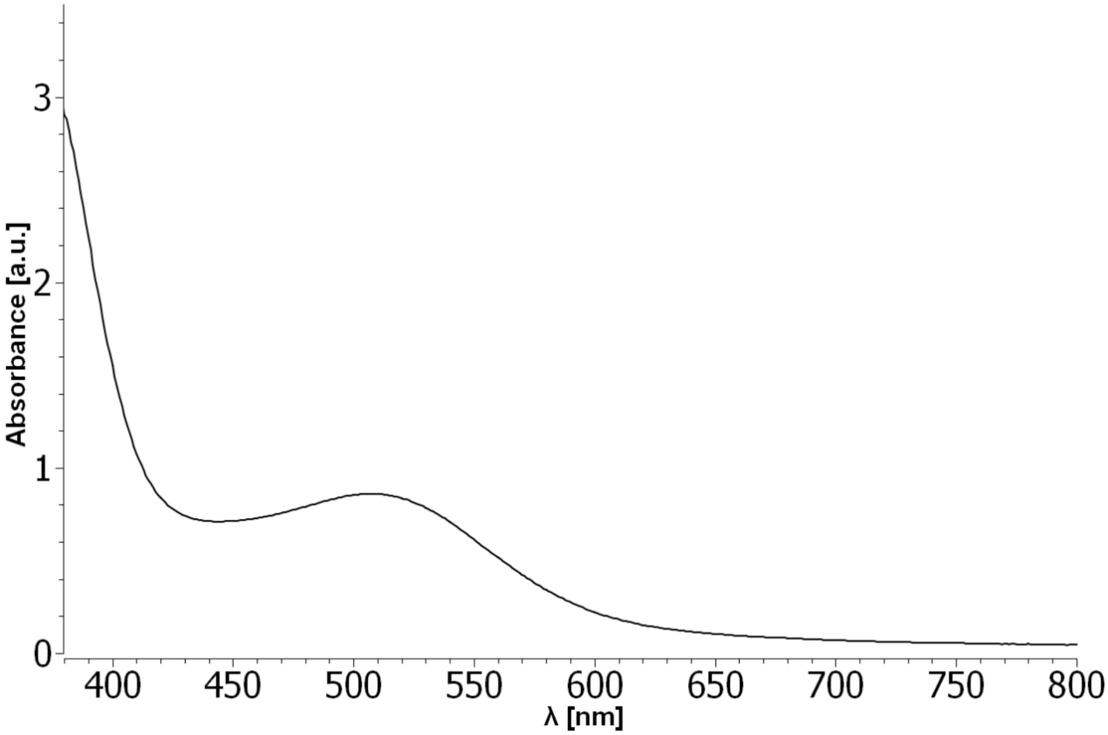

Figure S25: UV/Vis of 1-Ca.

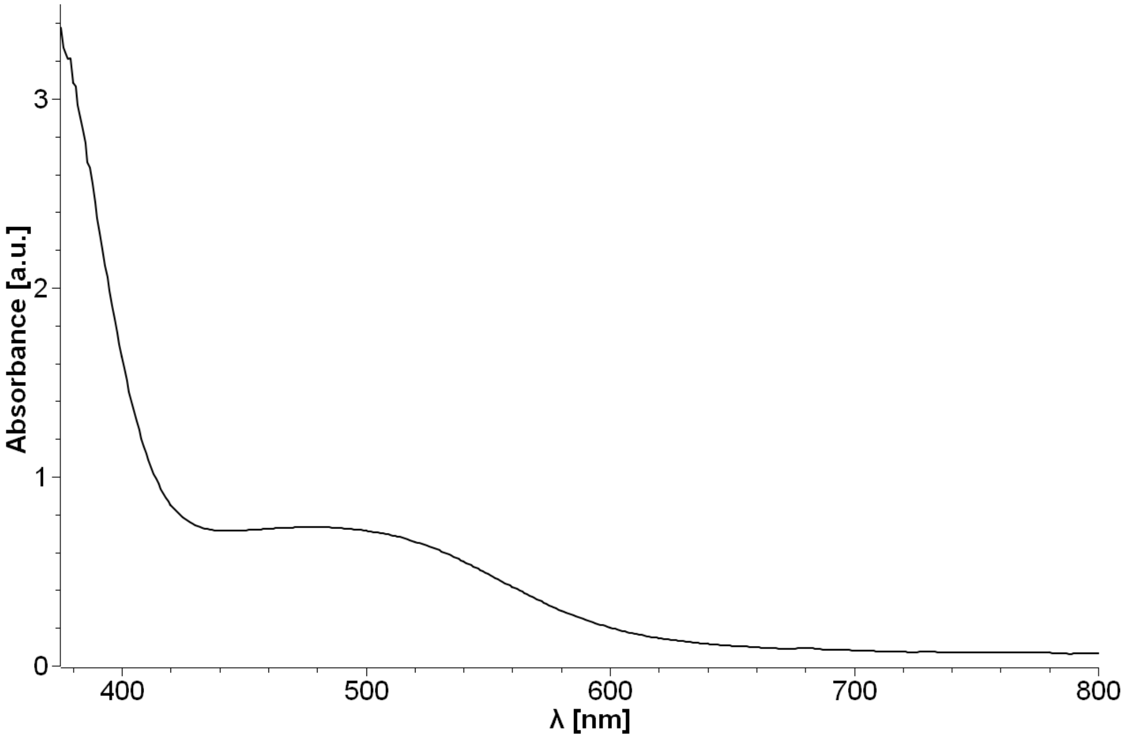

Figure S26: UV/Vis of 1-Sr.

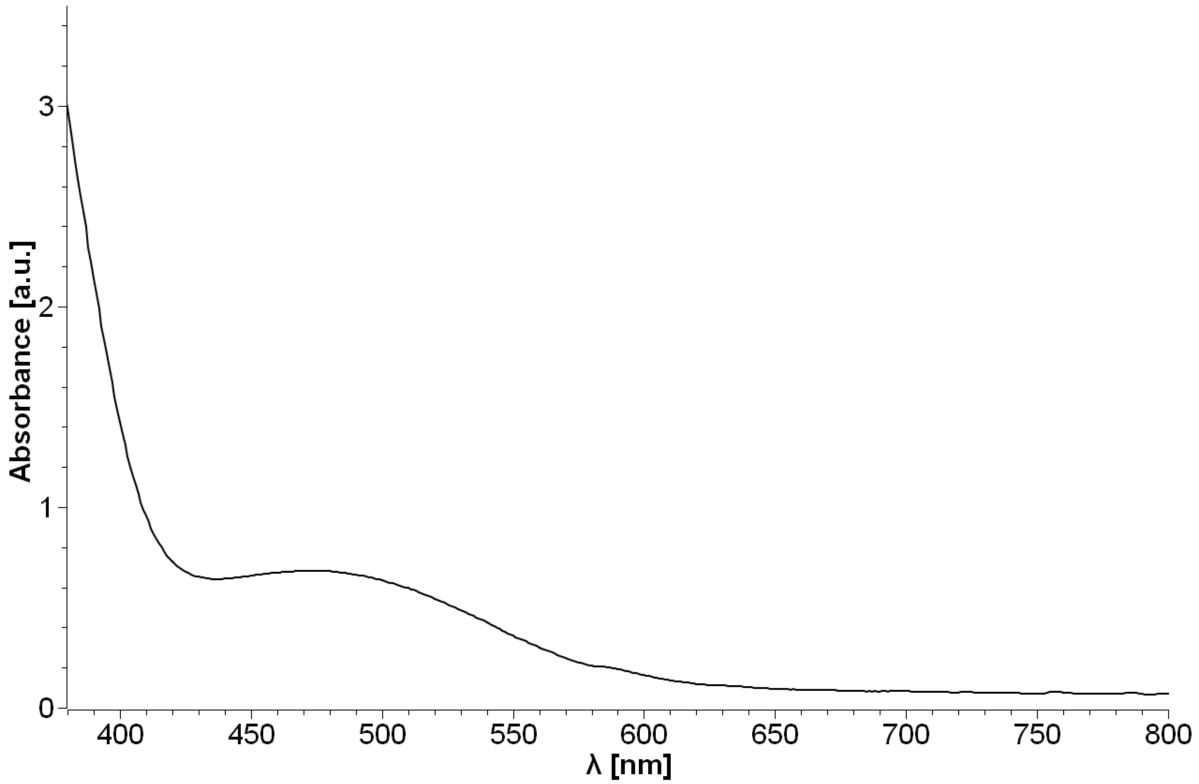

Figure S27: UV/Vis of 1-Ba.

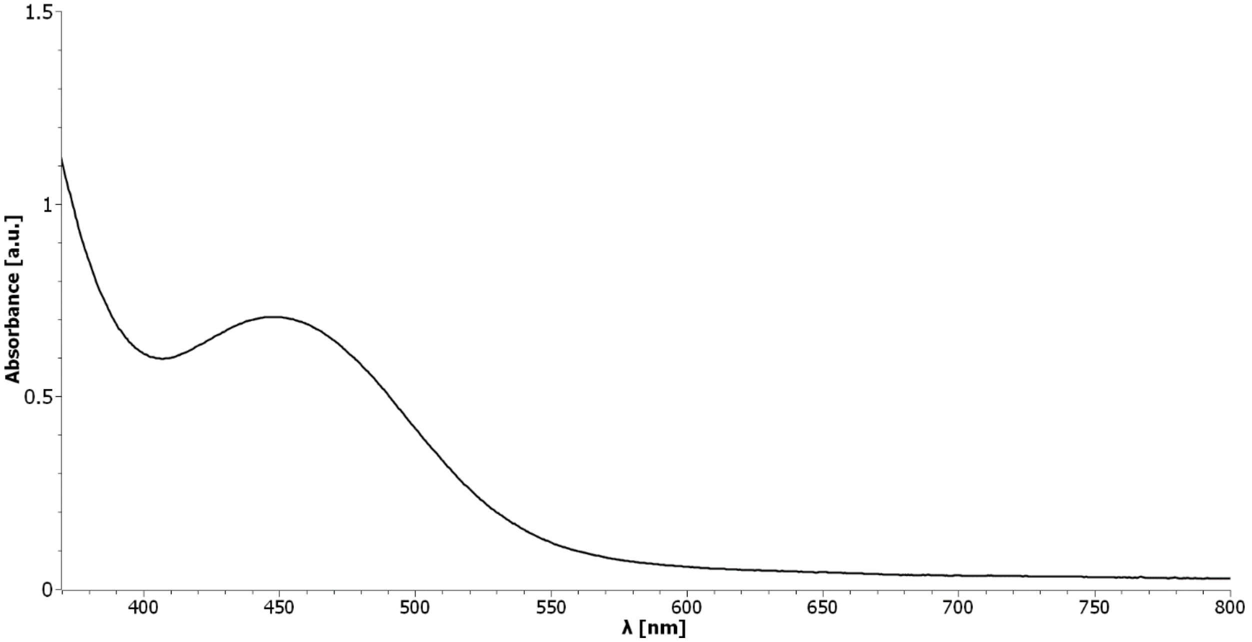

Figure S28: UV/Vis of 1-Eu.

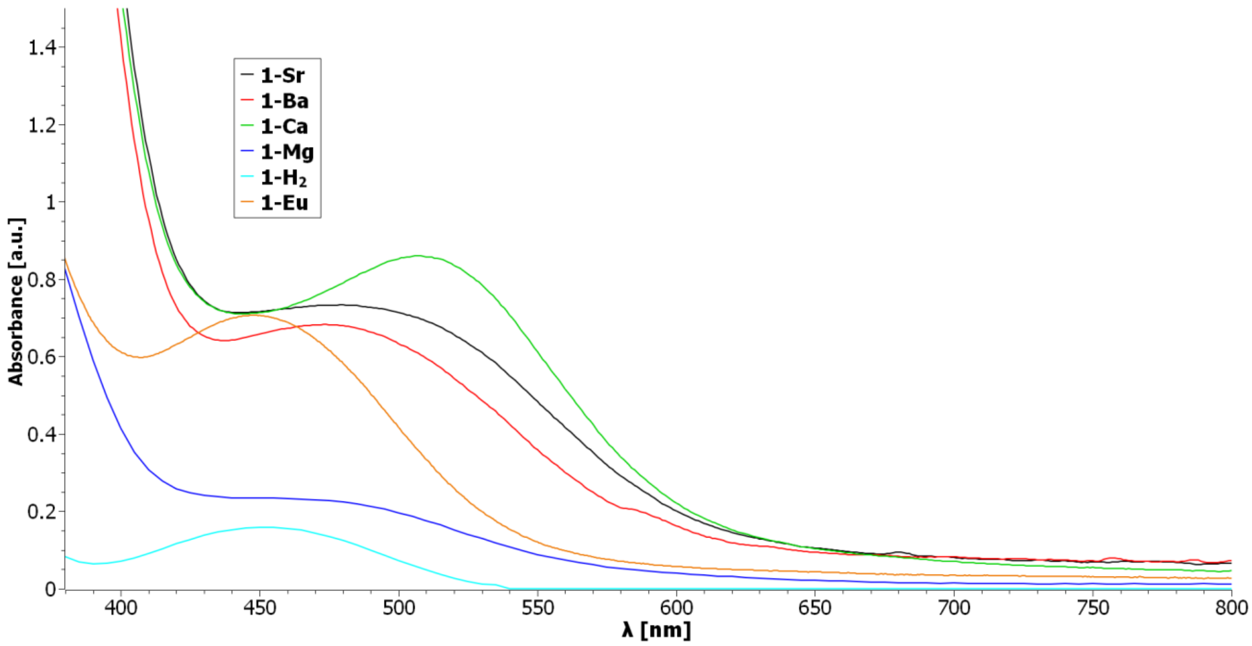

Figure S29: UV/Vis of 1-H_2, 1-Mg, 1-Ca, 1-Sr, 1-Ba, 1-Eu.

Table S7: Extinction coefficients and absorption maxima measured in pyridine.[a]

Metal	ε [L $mole^{-1}$ cm^{-1}]	λ [nm]	Concentration [mmol mL^{-1}]
H	198	450	8.05 *10^{-4}
Mg	275	455	8.55 *10^{-4}
Ca	602	506	1.43 *10^{-3}
Sr	484	479	1.53 *10^{-3}
Ba	651	474	1.07 *10^{-3}
Eu	679	447	1.04*10^{-3}

[a]Due to the very high air and water sensitivity the dilution of these highly colored solutions is limited by trace amounts of water (< 10 ppm) in the pyridine.

1.6 Cyclic Voltammetry

General Information

The cyclic voltammograms were recorded under rigorous exclusion of moisture and oxygen inside an argon filled glovebox with a PalmSens 4 (PalmSens) by working in freshly distilled and degassed THF (Sigma Aldrich, ACS reagent grade, distillation over sodium mirror, additional drying over activated MS 3Å; water content: below 8 ppm H_2O) with 0.1 M NBu_4PF_6 (dried, > 99.0%, electrochemical grade, Fluka) as electrolyte. The electrochemical cell was treated with *t*-BuLi (1.7M in pentane) and was washed with *n*-pentane and THF prior to its usage. Concentrations of the compounds were ~ $1{\cdot}10^{-4}$ M. A three-electrode setup was used with a glassy carbon working electrode, a coiled platinum wire as counter electrode, and a coiled silver wire as a pseudo-reference electrode. The ferrocene/ferrocenium couple was used as internal reference. The ferrocene was sublimated prior to its use. All determined values are depicted in table S8.

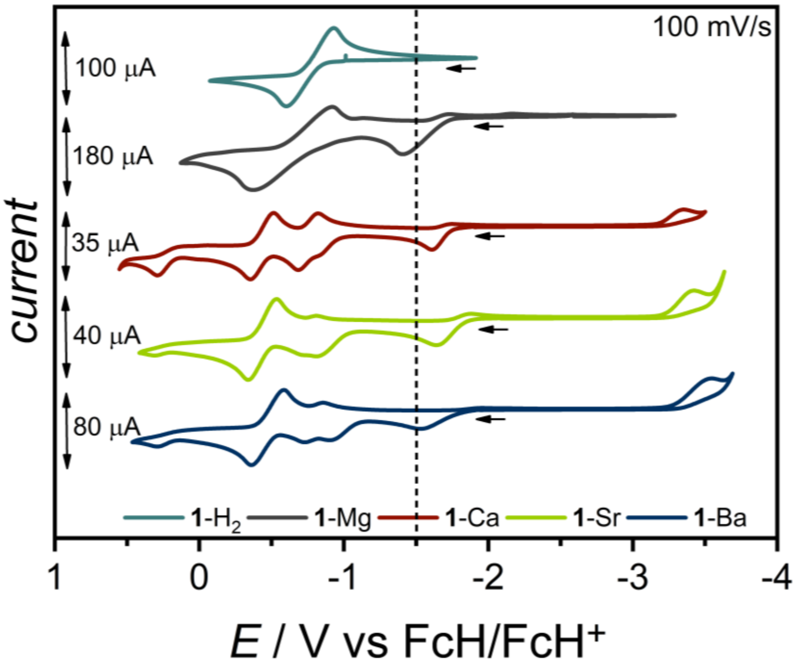

Figure S30: Cyclic voltammograms of the ligand **1**-H_2 and the complexes **1**-(Mg, Ca, Sr, Ba) in THF at 100 mV/s, NBu_4PF_6 as supporting electrolyte.

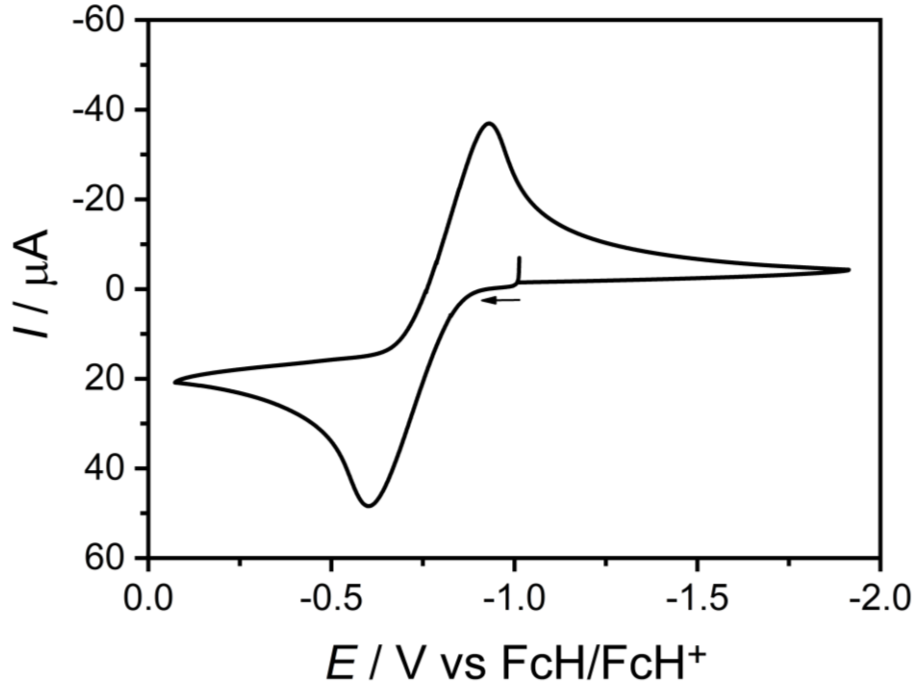

Figure S31: Cyclic voltammogram of the first oxidation of the ligand **1**-H_2 in THF at 100 mV/s, NBu_4PF_6 as supporting electrolyte.

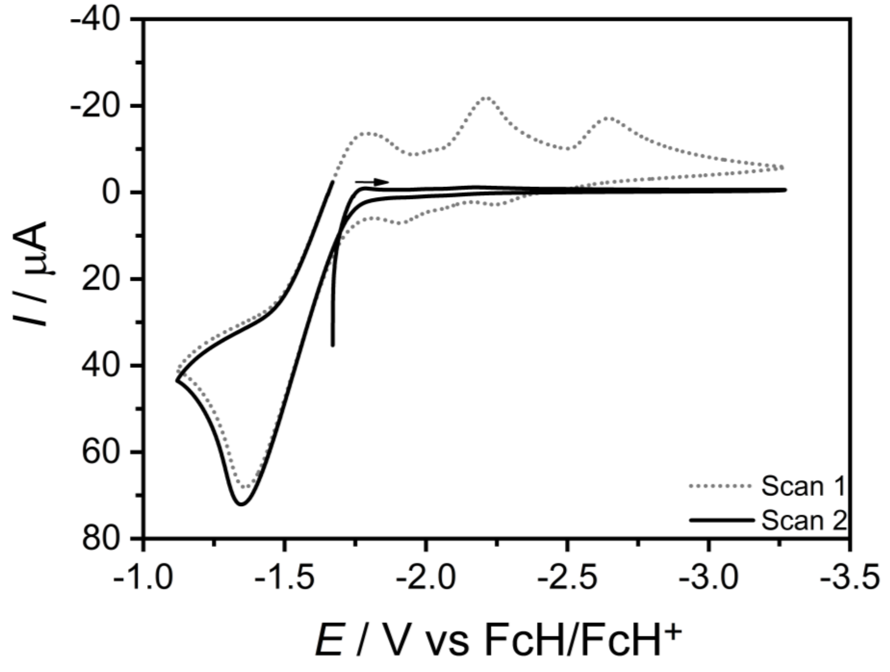

Figure S32: Cyclic voltammogram (scan 1 and 2) of the first oxidation (including rereduction processes) of complex **1**-Mg in THF at 100 mV/s, NBu_4PF_6 as supporting electrolyte.

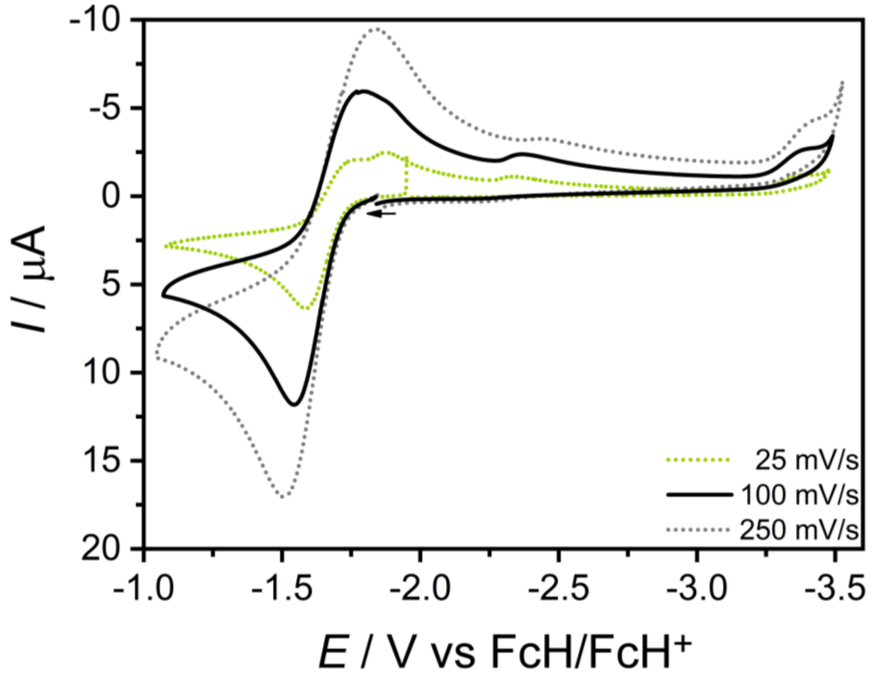

Figure S33: Cyclic voltammograms of the first oxidation (including rereduction processes) of complex **1**-Ca in THF at different scan rates as indicated in the legend, NBu_4PF_6 as supporting electrolyte.

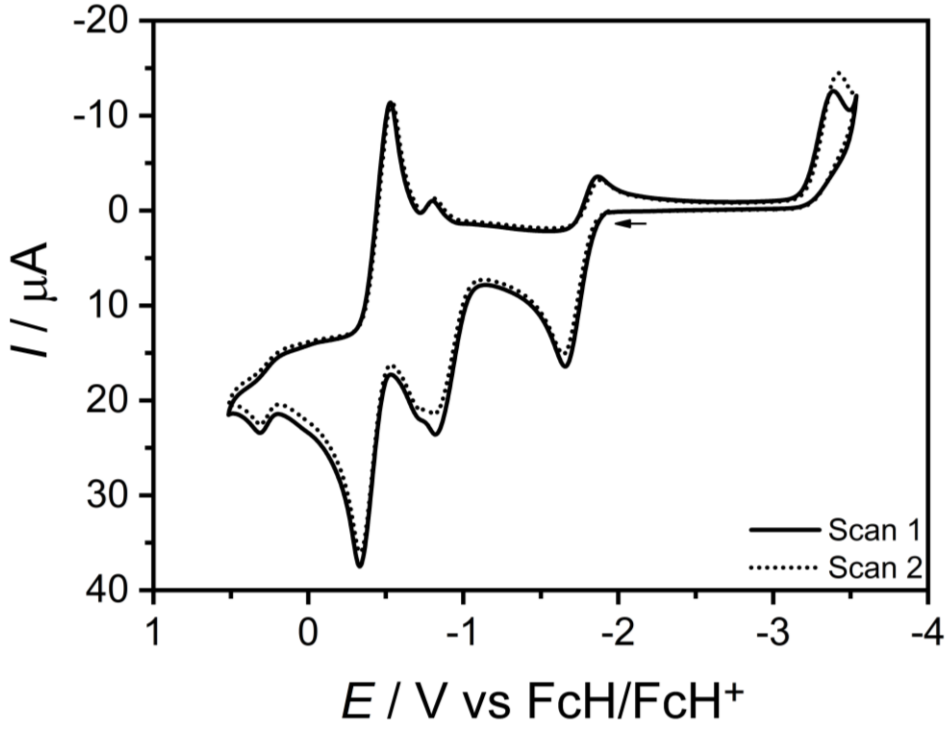

Figure S34: Cyclic voltammogram of complex **1**-Sr in THF at 100 mV/s, NBu_4PF_6 as supporting electrolyte.

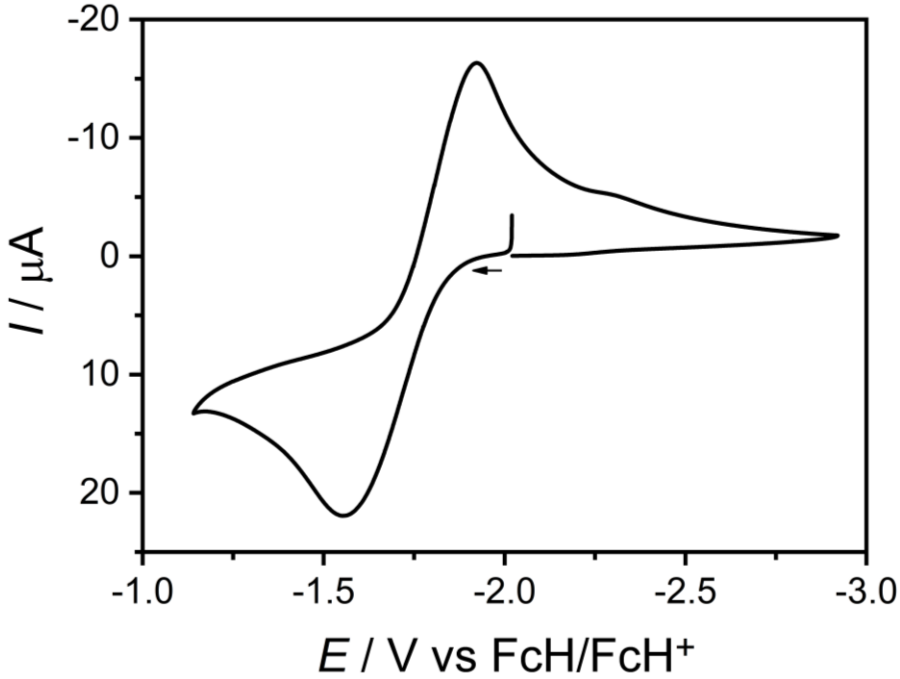

Figure S35: Cyclic voltammogram of the first oxidation of complex **1**-Ba in THF at 100 mV/s, NBu_4PF_6 as supporting electrolyte.

Table S8: Half-wave potentials and cathodic/anodic potentials of the compounds measured in THF (100 mV/s) with NBu_4PF_6 as electrolyte. The FcH/FcH$^+$ couple was used as an internal standard.

Metal	$E_{1/2}$(Ox1)	$E_{1/2}$(Ox2)	$E_{1/2}$(Ox3)	$E_{1/2}$(Ox4)	E_P(Ox5)	E_P(Rered)
**1**-H_2	–0.77	–	–	–	–	–
**1**-Mg	–1.57[a]	–0.97[c]	–0.65	–	–	–
**1**-Ba	–1.75	–0.89[b]	–0.77	–0.45	0.33[b]	–3.55[b]
**1**-Sr	–1.75	–0.82[b]	–0.75	–0.43	0.31[b]	–3.39[b]
**1**-Ca	–1.67[a]	-	–0.75[d]	–0.43	0.29[b]	–3.35[b]

[a] Half-wave potentials for irreversible processes
[b] Peak potentials for irreversible processes.
[c] Only visible at slow scan rates (25 mV/s)
[d] Probably contains two oxidation processes
[e] Also contains the irreversible reduction of pyridine

2. Computational Details

The geometry optimization followed by the harmonic vibrational frequencies calculations of **1**-Ae (Ae = Mg, Ca, Sr, Ba) complexes were performed at the BP86-D3(BJ)/def2-SVP level.[S10] Effective core potential was used for the 28 and 46 core electrons of Sr and Ba atoms, respectively, to take care of the relativistic effects. Superfine integration grid was used for all computations. All these calculations were carried out with Gaussian 16 program package.[S11] The natural bond orbital (NBO) analysis[S12] was done with the NBO 6.0 program.[S13] Quantum theory of atoms in molecules (QTAIM) analysis[S14] was performed with wave functions generated at the BP86-D3(BJ)/def2-SVP/x2c-SVPall//BP86-D3(BJ)/def2-SVP level, where an all-electron x2c-SVPall basis set was used for Sr and Ba atoms to avoid the use of ECP in the program, by using AIMALL program.[S15]

The bonding situations were analysed by means of an energy decomposition analysis (EDA)[S16] together with the natural orbitals for chemical valence (NOCV)[S17] method by using the ADF 2018.105 program package.[S18] The EDA-NOCV calculations were carried out at the BP86-D3(BJ)/TZ2P+-ZORA level[S19] using the BP86-D3(BJ)/def2-SVP optimized geometries. TZ2P+ is a triple-ζ quality basis set augmented by two sets of polarization functions. In this analysis, the intrinsic interaction energy ($\Delta E_{\rm int}$) between two fragments is divided into four energy components as follows:

$$\Delta E_{\rm int} = \Delta E_{\rm elstat} + \Delta E_{\rm Pauli} + \Delta E_{\rm orb} + \Delta E_{\rm disp} \qquad (1).$$

The electrostatic $\Delta E_{\rm elstat}$ term is originated from the quasiclassical electrostatic interaction between the unperturbed charge distributions of the prepared fragments, whereas the Pauli repulsion $\Delta E_{\rm Pauli}$ corresponds to the energy change associated with the transformation from the superposition of the unperturbed electron densities of the isolated fragments to the wavefunction, which properly obeys the Pauli principle through explicit antisymmetrization and renormalization of the production wavefunction. Since D3(BJ) is coupled with the functional, it gives additional dispersion contribution between two interacting fragments. The orbital term $\Delta E_{\rm orb}$ is originated from the mixing of orbitals, charge transfer and polarization between the isolated fragments, which can be further decomposed into contributions from each irreducible representation of the point group of the interacting system as follows:

$$\Delta E_{orb} = \sum_{r} \Delta E_{r} \quad (2)$$

The combination of the EDA with NOCV enables the partition of the total orbital interactions into pairwise contributions of the orbital interactions which is very vital to get a complete picture of the bonding. The charge deformation $\Delta\rho_k(r)$, resulting from the mixing of the orbital pairs $\psi_k(r)$ and $\psi_{-k}(r)$ of the interacting fragments presents the amount and the shape of the charge flow due to the orbital interactions (Equation 3), and the associated energy term ΔE_{orb} provides with the size of stabilizing orbital energy originated from such interaction (Equation 4).

$$\Delta\rho_{orb}(r) = \sum_{k} \Delta\rho_{k}(r) = \sum_{k=1}^{N/2} \nu_{k} [-\psi_{-k}^{2}(r) + \psi_{k}^{2}(r)] \quad (3)$$

$$\Delta E_{orb} = \sum_{k} \Delta E_{k}^{orb} = \sum_{k=1}^{N/2} \nu_{k} [-F_{-k,-k}^{TS} + F_{k,k}^{TS}] \quad (4)$$

More details about the EDA-NOCV method and its application are given in recent reviews articles.[S20]

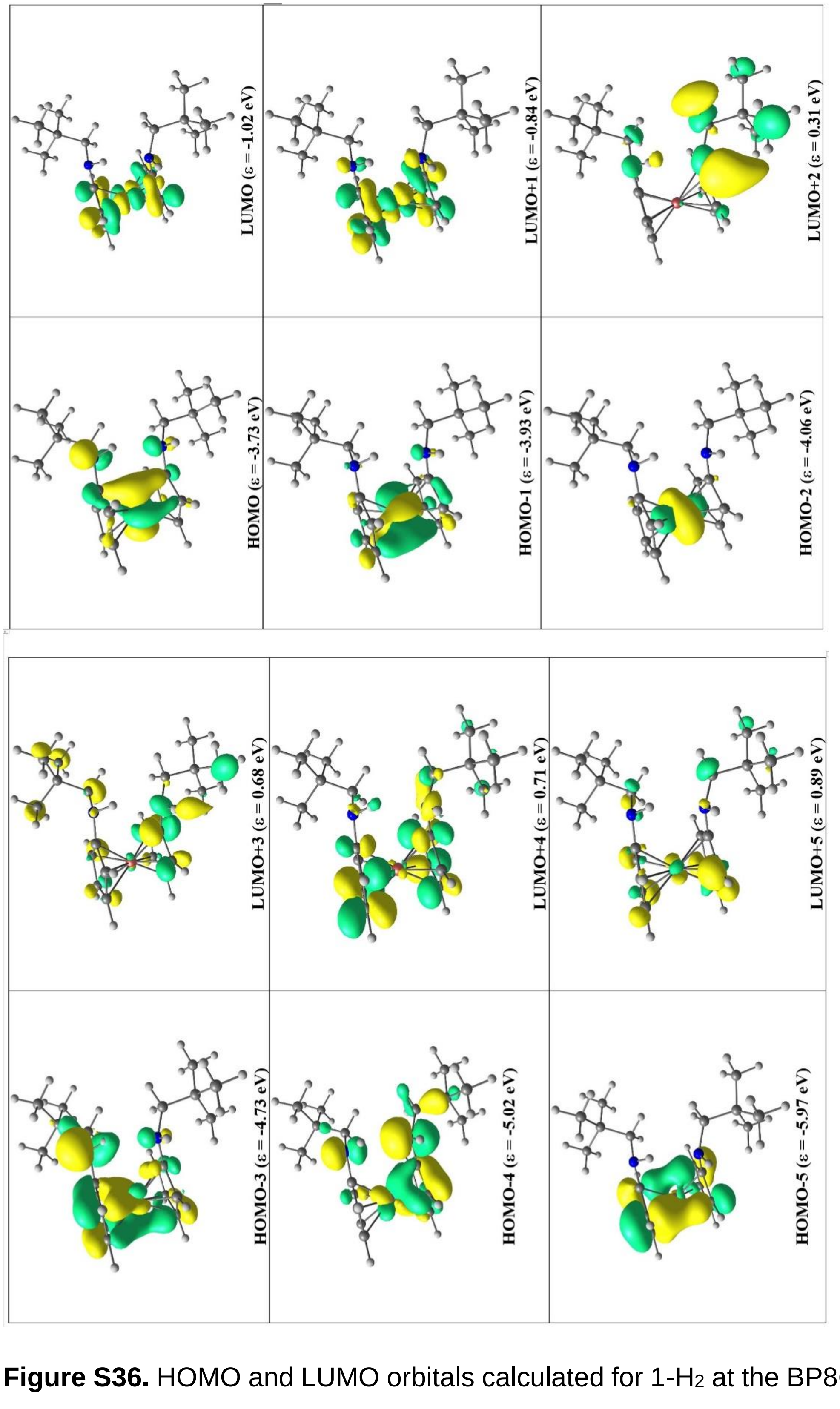

Figure S36. HOMO and LUMO orbitals calculated for 1-H_2 at the BP86-D3(BJ)/def2-SVP level. The MO isovalue is 0.04 au.

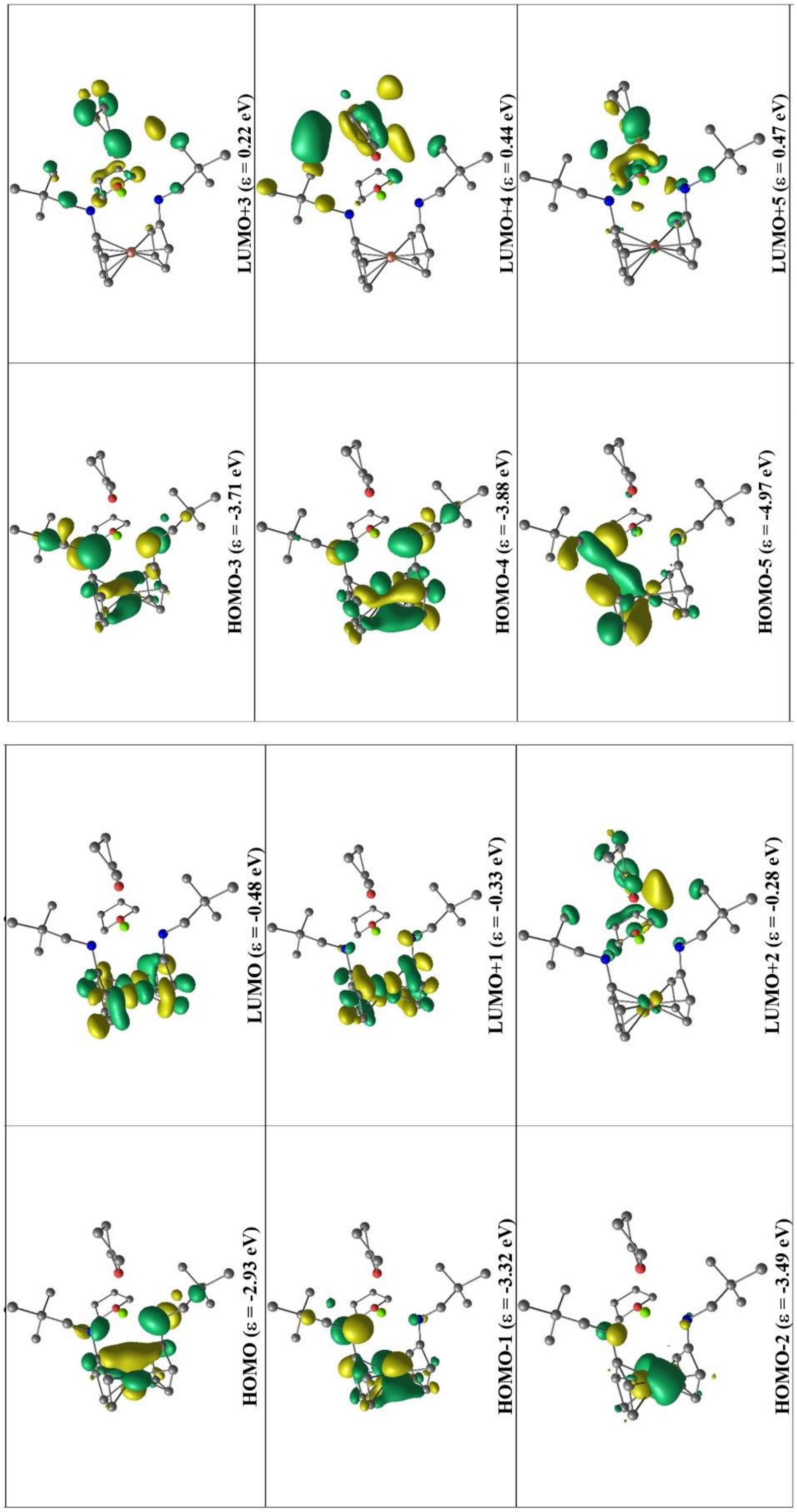

Figure S37. HOMO and LUMO orbitals calculated for 1-Mg at the BP86-D3(BJ)/def2-SVP level. The MO isovalue is 0.04 au.

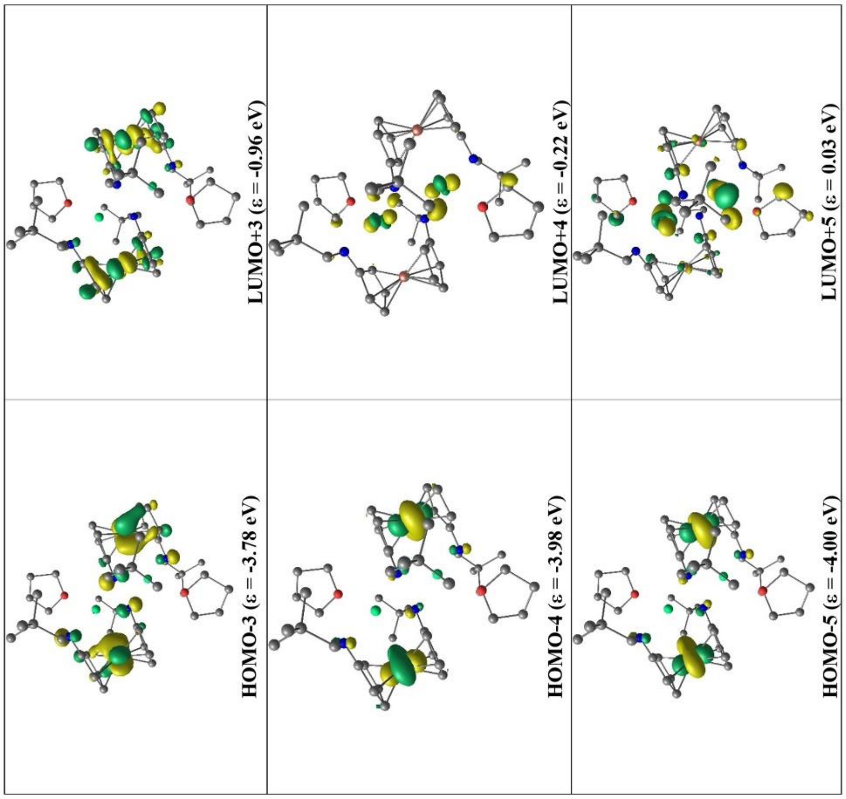

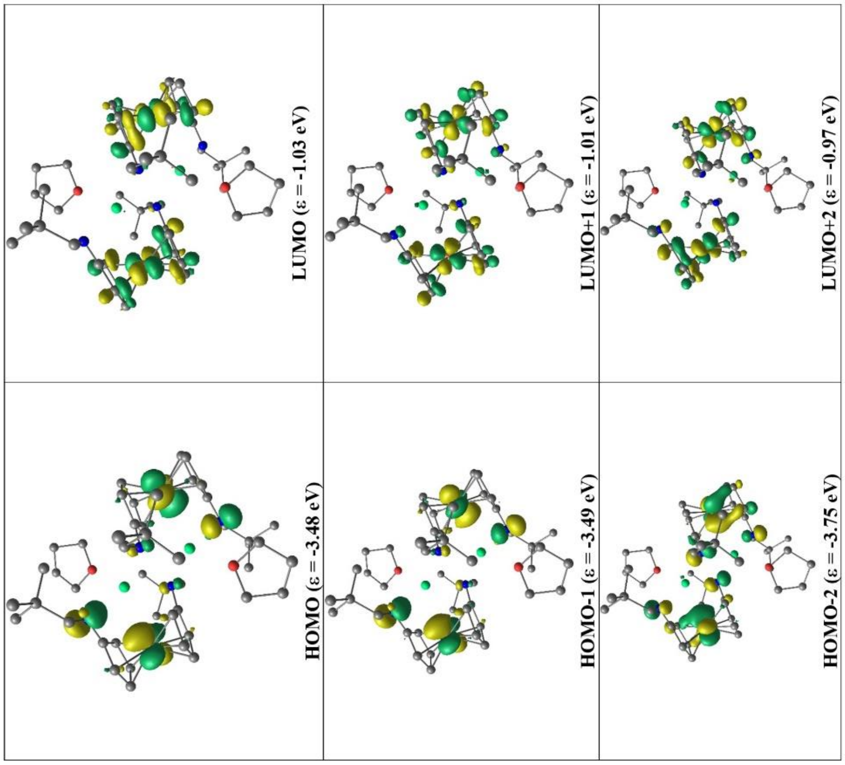

Figure S38. HOMO and LUMO orbitals calculated for 1-Ca at the BP86-D3(BJ)/def2-SVP level. The MO isovalue is 0.04 au.

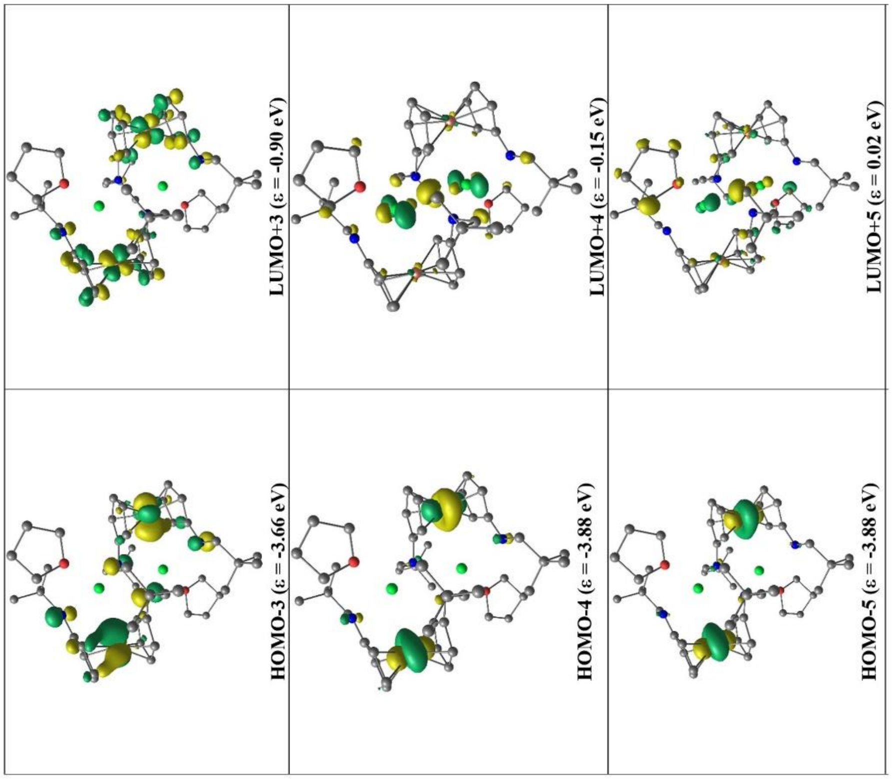

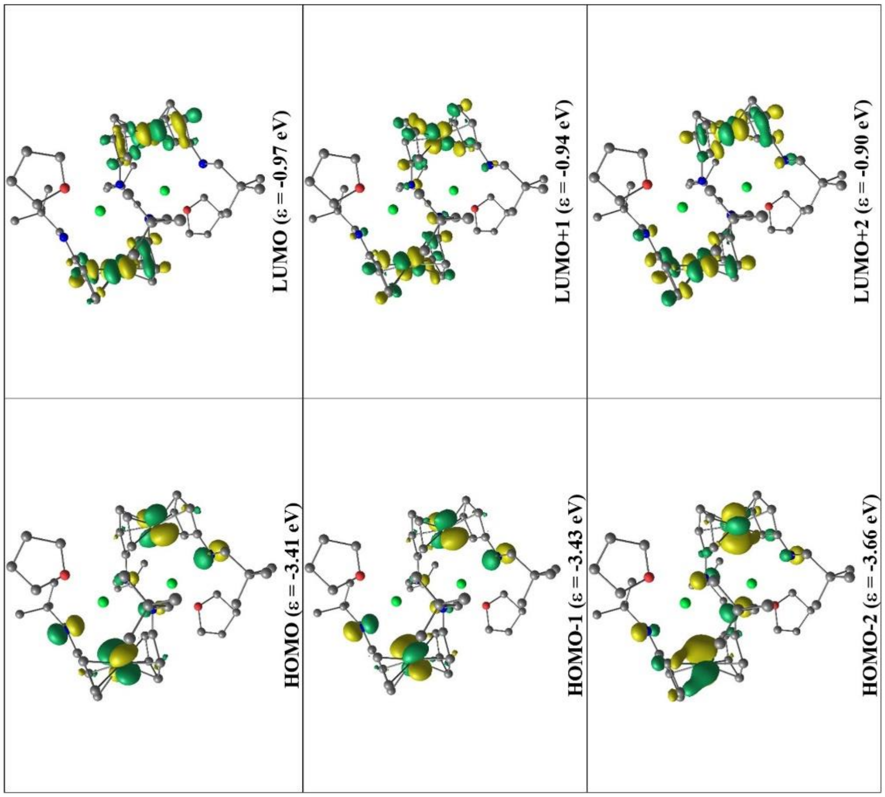

Figure S39. HOMO and LUMO orbitals calculated for 1-Sr at the BP86-D3(BJ)/def2-SVP level. The MO isovalue is 0.04 au.

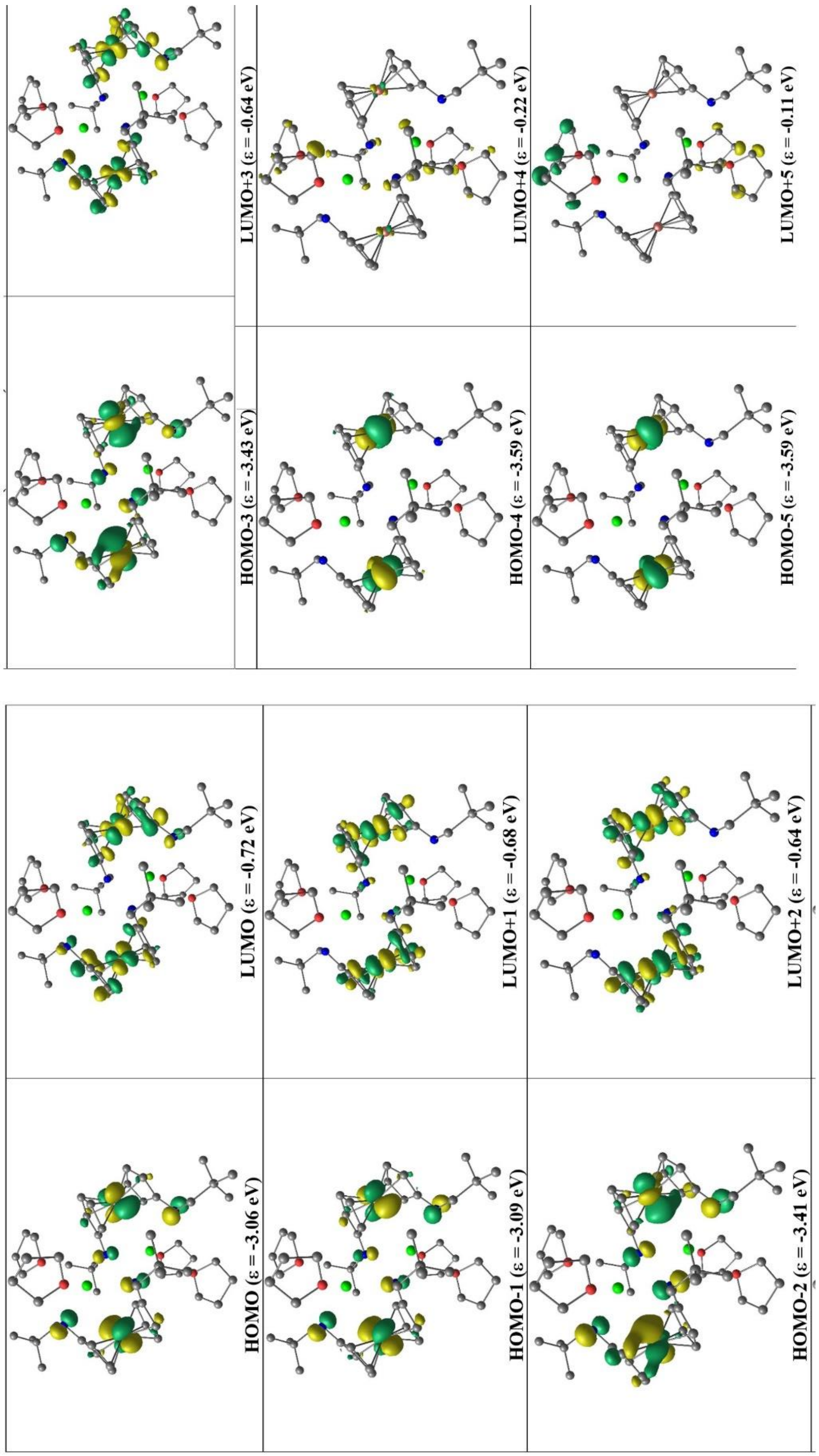

Figure S40. HOMO and LUMO orbitals calculated for 1-Ba at the BP86-D3(BJ)/def2-SVP level. The MO isovalue is 0.04 au.

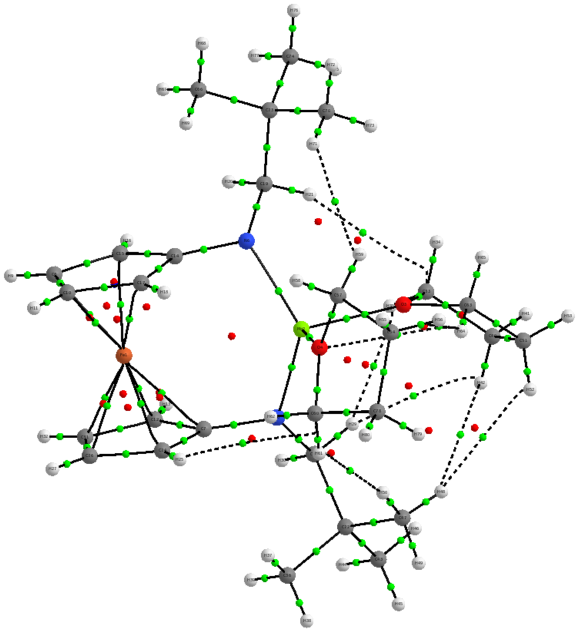

1-Mg

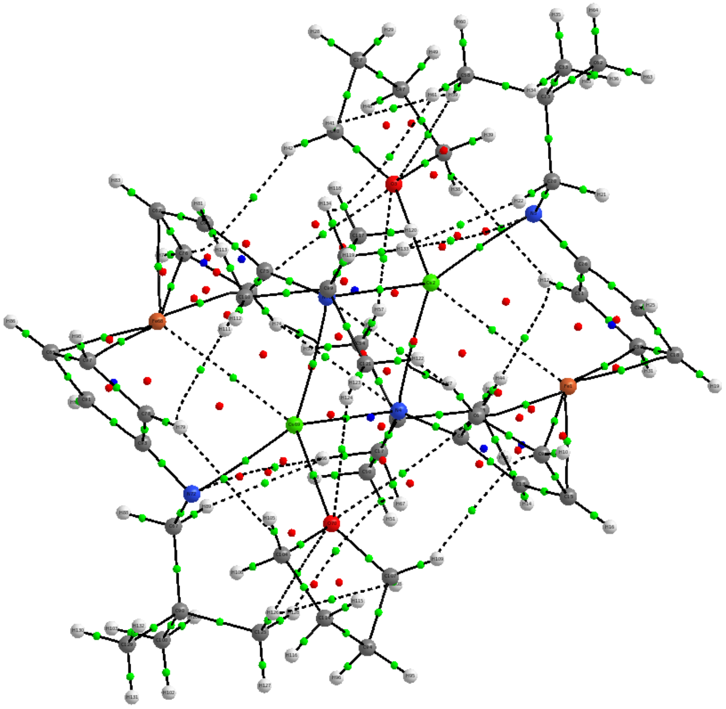

1-Ca

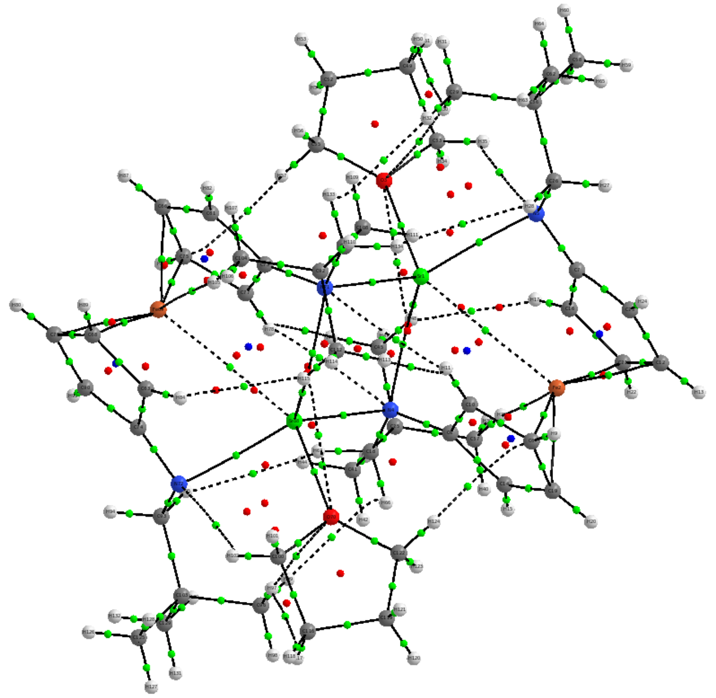

1-Sr

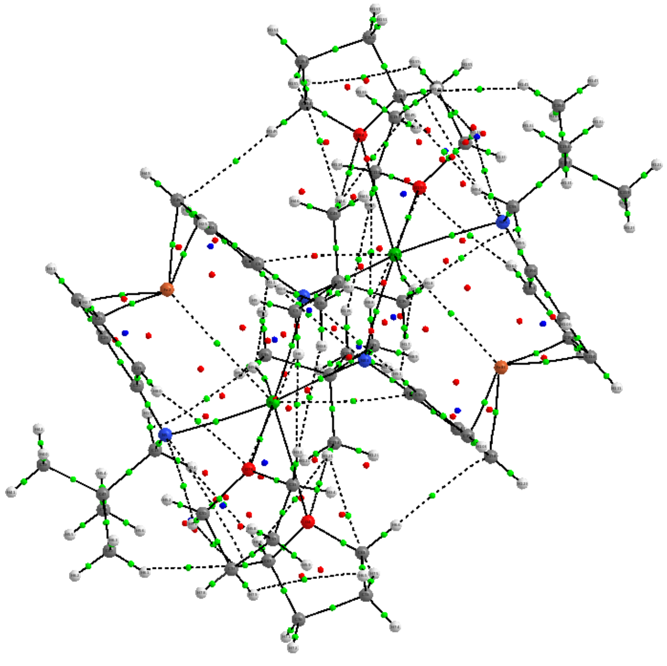

1-Ba

Figure S41. The molecular graphs of **1-**Ae (Ae = Mg, Ca, Sr and Ba) complexes at the BP86-D3(BJ)/def2-SVP/x2c-SVPall//BP86-D3(BJ)/def2-SVP level. Small green, red and blue circles represent bond critical point, ring critical point and cage critical points, respectively.

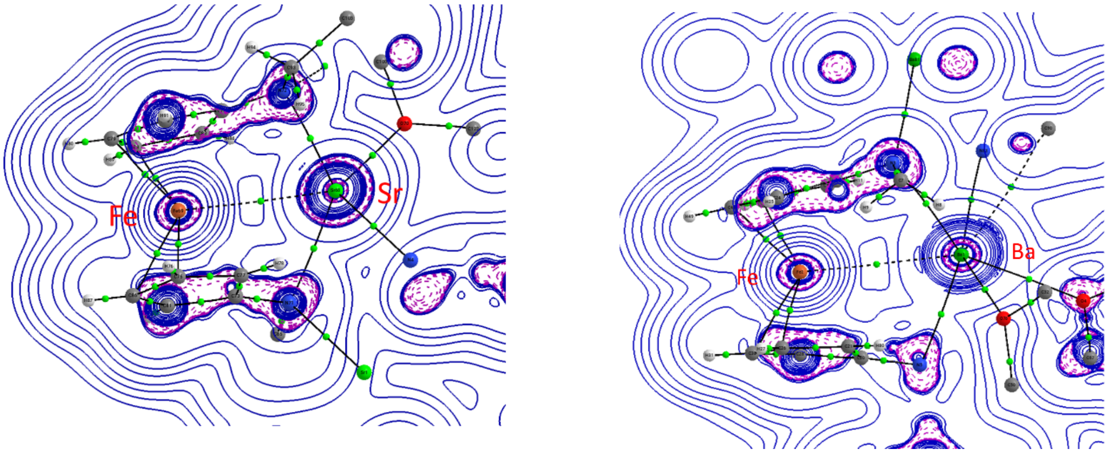

1-Sr

1-Ba

Figure S42. The contour plot of the Laplacian of electron density, $\nabla^2\rho(r)$ of **1**-Sr and **1**-Ba in the Fe-Ae-N plane. The blue solid lines indicate regions of charge depletion ($\nabla^2\rho(r) > 0$) and red dotted lines indicate regions of charge accumulation ($\nabla^2\rho(r) < 0$). Small green circles represent bond critical points.

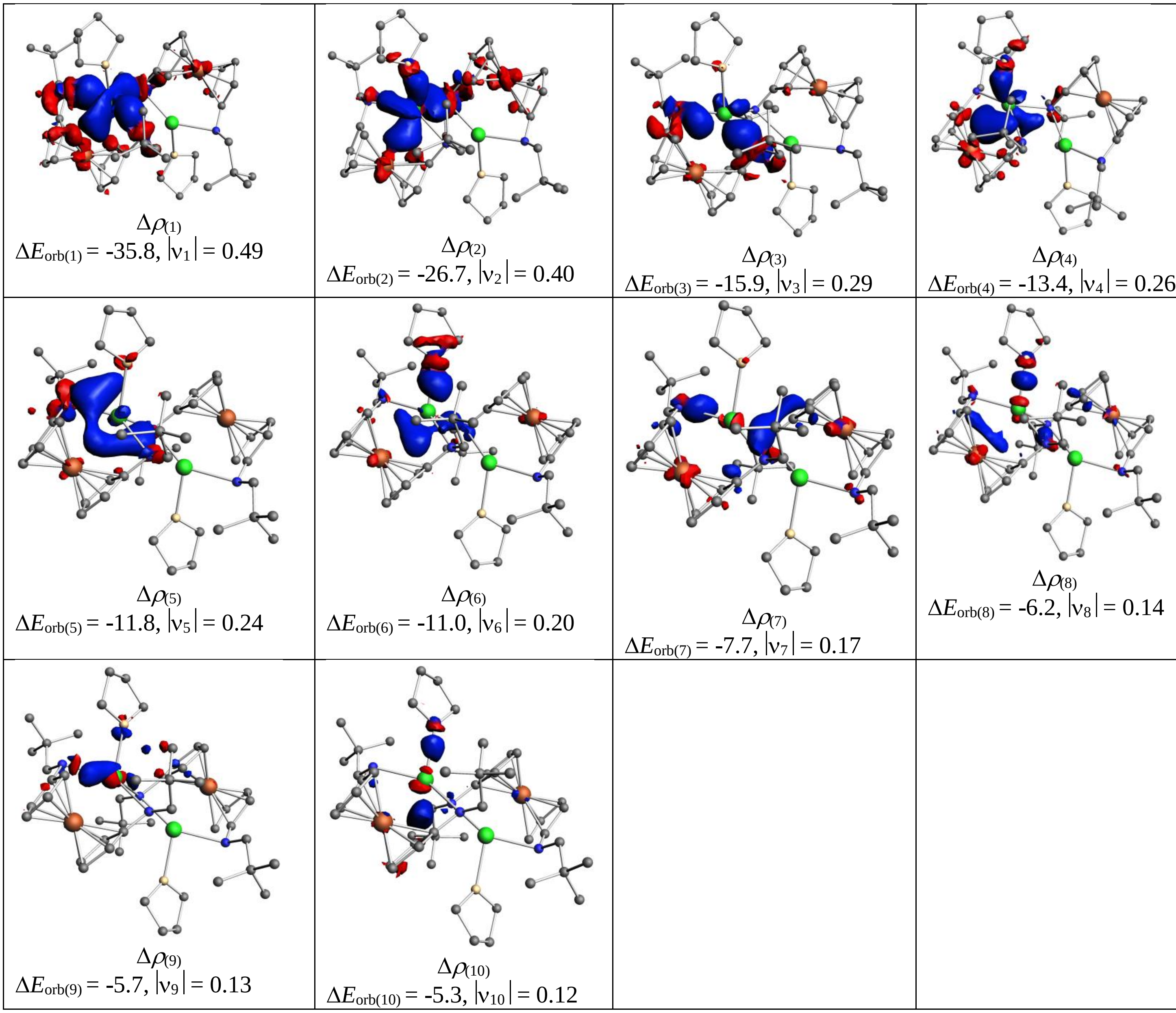

Figure S43. Shape of the deformation densities $\Delta\rho_{(1)\text{-}(10)}$, which are associated with the orbital interactions $\Delta E_{orb(1)\text{-}(10)}$ in **1-**Ca complex and eigenvalues $|\nu_n|$ of the charge flow. The isosurface value is 0.0006. The color code of the charge flow is red → blue.

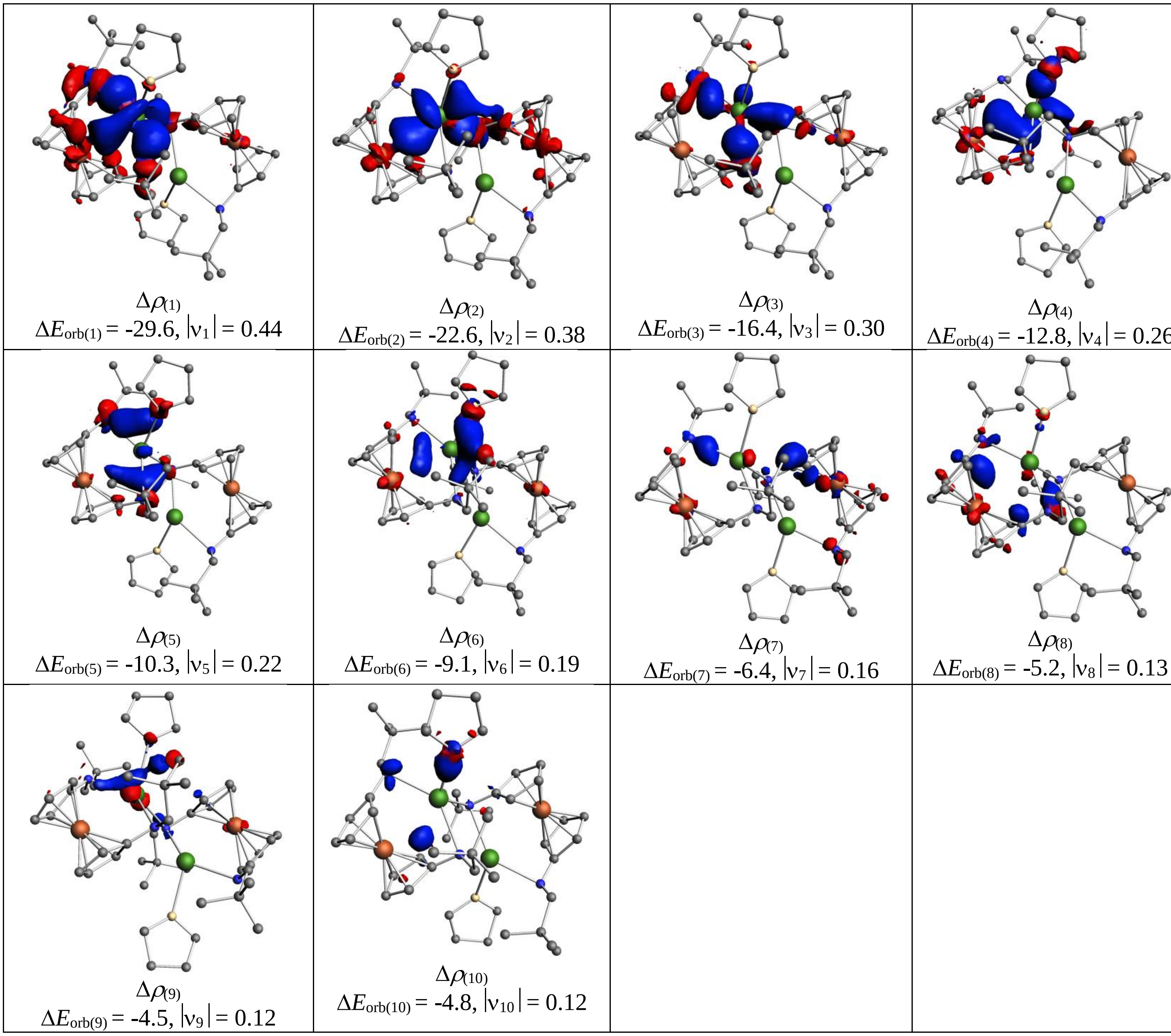

Figure S44. Shape of the deformation densities $\Delta\rho_{(1)\text{-}(10)}$, which are associated with the orbital interactions $\Delta E_{orb(1)\text{-}(10)}$ in **1-**Sr complex and eigenvalues $|\nu_n|$ of the charge flow. The isosurface value is 0.0006. The color code of the charge flow is red → blue.

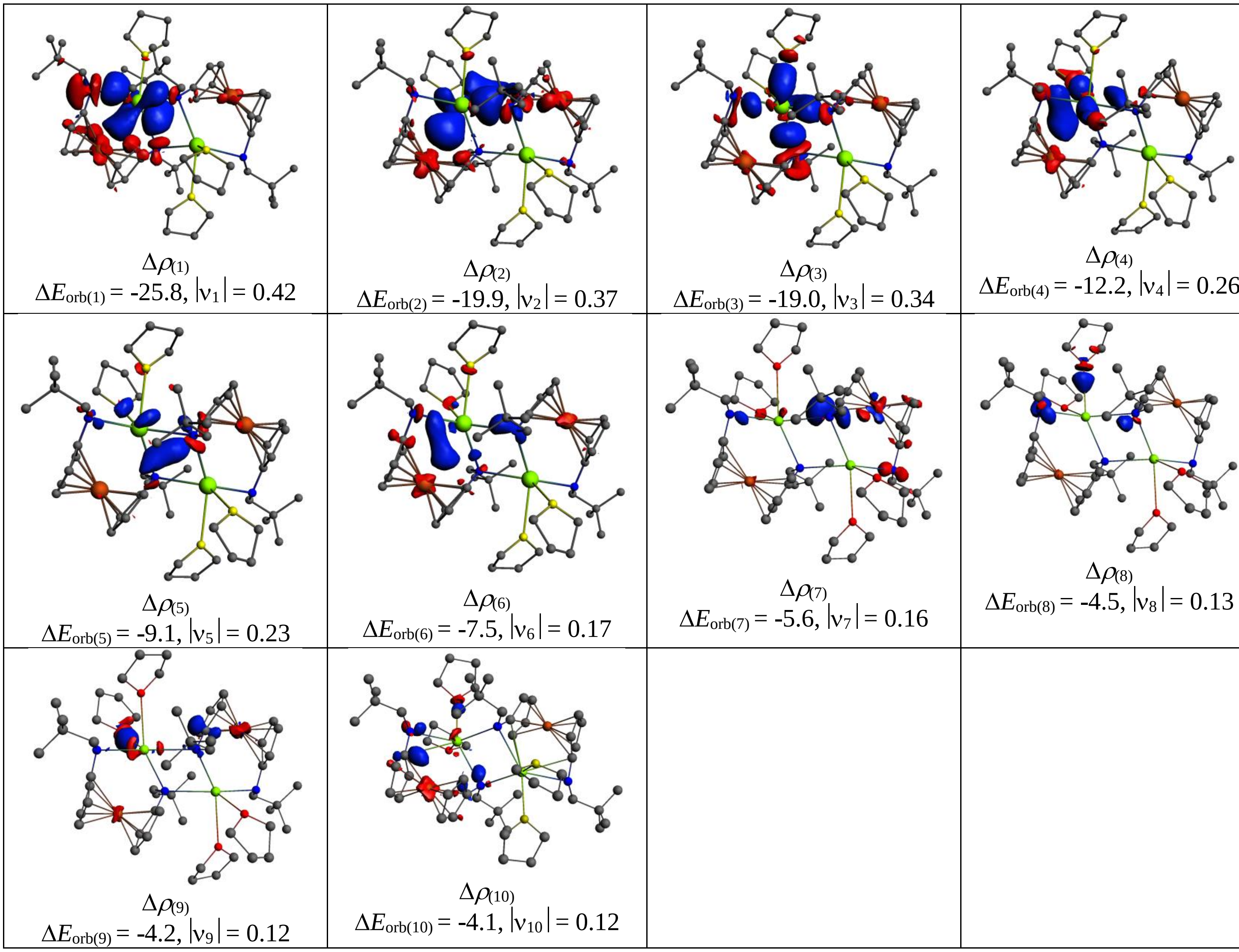

Figure S45. Shape of the deformation densities $\Delta\rho_{(1)\text{-}(10)}$, which are associated with the orbital interactions $\Delta E_{orb(1)\text{-}(10)}$ in **1-**Ba complex and eigenvalues $|\nu_n|$ of the charge flow. The isosurface value is 0.0006. The color code of the charge flow is red → blue.

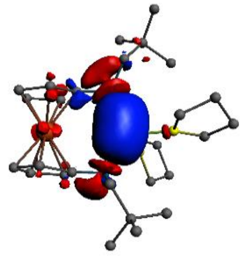<br>$\Delta\rho_{(1)}$<br>$\Delta E_{\mathrm{orb}(1)} = -33.6$, $\lvert\nu_1\rvert =$ 0.42	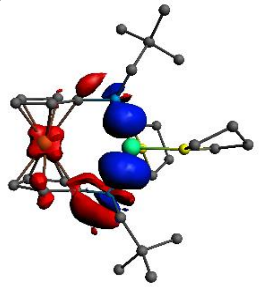<br>$\Delta\rho_{(2)}$<br>$\Delta E_{\mathrm{orb}(2)} = -32.2$, $\lvert\nu_2\rvert =$ 0.37	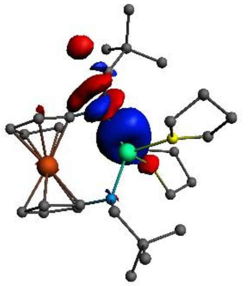<br>$\Delta\rho_{(3)}$<br>$\Delta E_{\mathrm{orb}(3)} = -22.8$, $\lvert\nu_3\rvert =$ 0.33	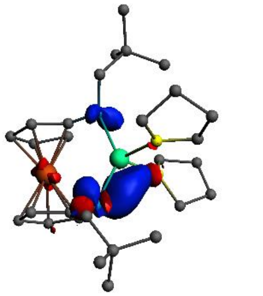<br>$\Delta\rho_{(4)}$<br>$\Delta E_{\mathrm{orb}(4)} = -16.4$, $\lvert\nu_4\rvert =$ 0.27
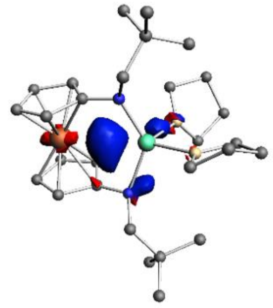<br>$\Delta\rho_{(5)}$<br>$\Delta E_{\mathrm{orb}(5)} = -13.8$, $\lvert\nu_5\rvert =$ 0.23	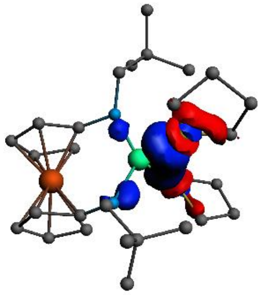<br>$\Delta\rho_{(6)}$<br>$\Delta E_{\mathrm{orb}(6)} = -17.1$, $\lvert\nu_6\rvert =$ 0.22	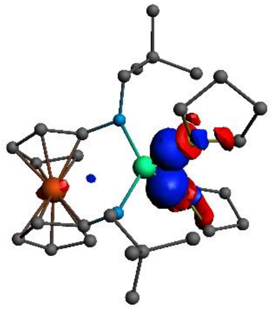<br>$\Delta\rho_{(7)}$<br>$\Delta E_{\mathrm{orb}(7)} = -13.9$, $\lvert\nu_7\rvert =$ 0.20	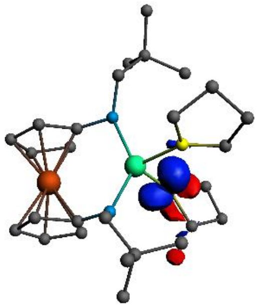<br>$\Delta\rho_{(8)}$<br>$\Delta E_{\mathrm{orb}(8)} = -7.5$, $\lvert\nu_8\rvert =$ 0.15
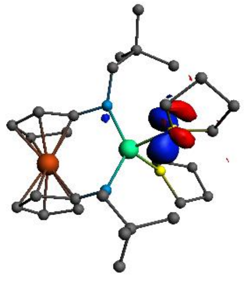<br>$\Delta\rho_{(9)}$<br>$\Delta E_{\mathrm{orb}(9)} = -7.0$, $\lvert\nu_9\rvert =$ 0.14	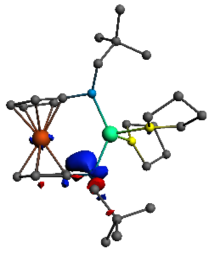<br>$\Delta\rho_{(10)}$<br>$\Delta E_{\mathrm{orb}(10)} = -4.9$, $\lvert\nu_{10}\rvert =$ 0.11		

Figure S46. Shape of the deformation densities $\Delta\rho_{(1)\text{-}(10)}$, which are associated with the orbital interactions $\Delta E_{\mathrm{orb}(1)\text{-}(10)}$ in **1-**Mg complex and eigenvalues $\lvert\nu_n\rvert$ of the charge flow. The isosurface value is 0.001. The color code of the charge flow is red → blue.

$\Delta\rho$	$[Cp^*_2Fe(THF)_2]^{2-}$	$[Mg]^{2+}$
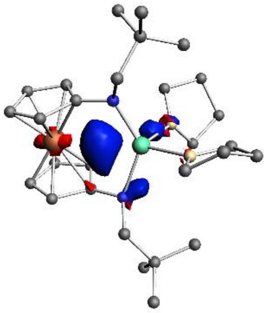 $\Delta E_{orb(5)}$ = -13.8, $\lvert\nu_5\rvert$ = 0.23	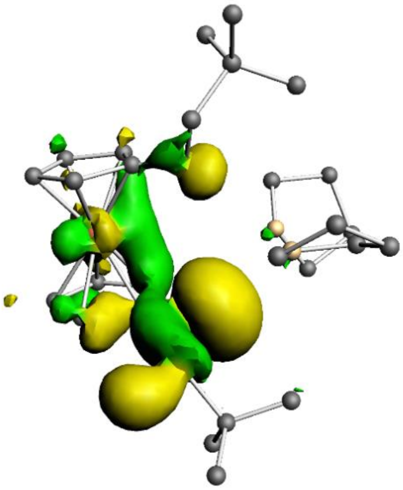 HOMO-6	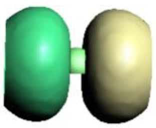 LUMO+1

Figure S47. Shape of the deformation densities $\Delta\rho_{(5)}$ and the shape of the associated orbitals of the fragments in **1**-Mg complex and eigenvalues $\lvert\nu_n\rvert$ of the charge flow. The MO energy eigen values are in eV.

Table S9. Some selected experimental and computed geometrical parameters of **1**-Ae complex. Bond distances are in Å and bond angles are in degree.

Complex	Parameter	Experimental	Computed
	Mg-Fe	3.426	3.259

**1**-Mg	Mg-N1	1.987	1.997
	Mg-N2	1.975	1.977
	Mg-O1	2.041	2.083
	Mg-O2	2.049	2.061
	<N1MgN2	121.7	130.8
	<FeMgN1	65.8	70.1
	<FeMgN2	65.2	69.1
**1**-Ca	Ca-Fe	3.113	3.061
	Ca-N1	2.371	2.370
	Ca-N2	2.502	2.475
	Ca-O1	2.359	2.372
	Ca-N2′	2.407	2.389
	<N1CaN2	140.2	142.8
	<FeCaN1	70.8	72.4
	<FeCaN2	69.4	70.6
**1**-Sr	Sr-Fe	3.320	3.198
	Sr-N1	2.516	2.516
	Sr-N2	2.603	2.610
	Sr-O1	2.496	2.524
	Sr-N2′	2.560	2.556
	<N1SrN2	132.2	137.2
	<FeSrN1	67.1	70.0
	<FeSrN2	68.1	67.7
**1**-Ba	Ba-Fe	3.454	3.442
	Ba-N1	2.676	2.693
	Ba-N2	2.751	2.776
	Ba-O1	2.830	2.765
	Ba-O2	2.814	2.809
	Ba-N2′	2.765	2.748
	<N1BaN2	127.5	126.9
	<FeBaN1	65.4	65.8
	<FeBaN2	62.6	62.1

Table S10. The results of EDA for **1-**Mg complex taking different partitioning schemes at the BP86-D3(BJ)/TZ2P+//BP86-D3(BJ)/def2-SVP level.

Energies	Mg^{2+} (S, $3s^0$) + $[Cp*_2Fe(THF)_2]^{2-}$ (S)	Mg^+ (D, $3s^1$) + $[Cp*_2Fe(THF)_2]^-$ (D)	Mg (S, $3s^2$) + $[Cp*_2Fe(THF)_2]$ (S)	Mg (T, $3s^13p^1$) + $[Cp*_2Fe(THF)_2]$ (T)
ΔE_{int}	-687.9	-285.1	-154.0	-214.4
ΔE_{Pauli}	91.8	270.2	504.3	562.3
ΔE_{disp}	-16.2	-16.2	-16.2	-16.2
ΔE_{elstat}	-525.1	-300.3	-285.6	-349.3
ΔE_{orb}	-238.3	-238.9	-356.5	-411.3

Table S11. The results of EDA for **1-**Ca complex taking different partitioning schemes at the BP86-D3(BJ)/TZ2P+//BP86-D3(BJ)/def2-SVP level.

Energies	$[Ca]^+$ (D, $4s^04p^03d^1$) + $[Ca(Cp*_2Fe(THF))_2]^-$ (D)	$[Ca]^+$ (D, $4s^1$) + $[Ca(Cp*_2Fe(THF))_2]^-$ (D)	$[Ca]^{2+}$ (S, $4s^0$) + $[Ca(Cp*_2Fe(THF))_2]^{2-}$ (S)	[Ca] (S, $4s^2$) + $[Ca(Cp*_2Fe(THF))_2]$ (S)	[Ca] (S, $4s^04p^03d^2$) + $[Ca(Cp*_2Fe(THF))_2]$ (S)	[Ca] (T, $4s^14p^03d^1$) + $[Ca(Cp*_2Fe(THF))_2]$ (T)	[Ca] (T, $4s4p^03d^2$) [illegible] $[Ca(Cp*_2Fe($[illegible] $))_2]$ (T)
ΔE_{int}	-302.5	-275.0	-576.0	-189.2	-327.7	-237.9	-320.0
ΔE_{Pauli}	145.3	294.9	106.4	555.5	299.6	391.5	235.3
ΔE_{disp}	-26.4	-26.4	-26.4	-26.4	-26.4	-26.4	-26.4
ΔE_{elstat}	-195.6	-285.6	-432.9	-338.0	-228.0	-230.9	-139.7
ΔE_{orb}	-225.9	-257.9	-223.3	-380.3	-372.9	-372.1	-389.3

Table S12. The results of EDA for **1-**Sr complex taking different partitioning schemes at the BP86-D3(BJ)/TZ2P+//BP86-D3(BJ)/def2-SVP level.

Energies	$[Sr]^+$ (D, $5s^05p^04d^1$) + $[Sr(Cp*_2Fe(THF))_2]^-$ (D)	$[Sr]^+$ (D, $5s^1$) + $[Sr(Cp*_2Fe(THF))_2]^-$ (D)	$[Sr]^{2+}$ (S, $5s^0$) + $[Sr(Cp*_2Fe(THF))_2]^{2-}$ (S)
ΔE_{int}	-295.0	-256.6	-537.3
ΔE_{Pauli}	131.1	291.1	113.5
ΔE_{disp}	-28.7	-28.7	-28.7

ΔE_{elstat}	-183.9	-279.6	-420.4
ΔE_{orb}	-213.5	-239.4	-201.7

Table S13. The results of EDA for 1-**Ba** complex taking different partitioning schemes at the BP86-D3(BJ)/TZ2P+//BP86-D3(BJ)/def2-SVP level.

Energies	$[Ba]^+$ (D, $6s^06p^05d^1$) + $[Ba(Cp*_2Fe(THF)_2)_2]^-$ (D)	$[Ba]^+$ (D, $6s^1$) + $[Ba(Cp*_2Fe(THF)_2)_2]^-$ (D)	$[Ba]^{2+}$ (S, $6s^0$) + $[Ba(Cp*_2Fe(THF)_2)_2]^{2-}$ (S)
ΔE_{int}	-283.8	-272.4	-524.0
ΔE_{Pauli}	172.0	298.9	119.7
ΔE_{disp}	-31.7	-31.7	-31.7
ΔE_{elstat}	-220.7	-292.1	-418.2
ΔE_{orb}	-203.5	-247.6	-193.9

Table S14. The results of EDA-NOCV for **1-**Ae (Ae = Mg, Ca, Sr, and Ba) complexes taking Ae^{2+} as one fragment and the rest as another at the BP86-D3(BJ)/TZ2P+//BP86-D3(BJ)/def2-SVP level.

Energies	$[Mg]^{2+}$ (S, $3s^0$) + $[Cp^*_2Fe(THF)_2]^{2-}$ (S)	$[Ca]^{2+}$ (S, $4s^0$) + $[Ca(Cp^*_2Fe(THF))_2]^{2-}$ (S)	$[Sr]^{2+}$ (S, $5s^0$) + $[Sr(Cp^*_2Fe(THF))_2]^{2-}$ (S)	$[Ba]^{2+}$ (S, $6s^0$) + $[Ba(Cp^*_2Fe(THF)_2)_2]^{2-}$ (S)
ΔE_{int}	-687.9	-576.0	-537.3	-524.0
ΔE_{Pauli}	91.8	106.4	113.5	119.7
ΔE_{disp}[a]	-16.2 (2.1%)	-26.4 (3.9%)	-28.7 (4.4%)	-31.7 (4.9%)
ΔE_{elstat}[a]	-525.1 (67.4%)	-432.9 (63.4%)	-420.4 (64.6%)	-418.2 (65.0%)
ΔE_{orb}[a]	-238.3 (30.6%)	-223.3 (32.7%)	-201.7 (31.0%)	-193.9 (30.1%)
$\Delta E_{orb(1)}$[b]	-33.6 (14.1%)	-35.8 (16.0%)	-29.6 (14.7%)	-25.8 (13.3%)
$\Delta E_{orb(2)}$[b]	-32.2 (13.5%)	-26.7 (12.0%)	-22.6 (11.2%)	-19.9 (10.3%)
$\Delta E_{orb(3)}$[b]	-22.8 (9.6%)	-15.9 (7.1%)	-16.4 (8.1%)	-19.0 (9.8%)
$\Delta E_{orb(4)}$[b]	-16.4 (6.9%)	-13.4 (6.0%)	-12.8 (6.3%)	-12.2 (6.3%)
$\Delta E_{orb(5)}$[b]	-13.8 (5.8%)	-11.8 (5.3%)	-10.3 (5.1%)	-9.1 (4.7%))
$\Delta E_{orb(6)}$[b]	-17.1 (7.2%)	-11.0 (4.9%)	-9.1 (4.5%)	-7.5 (3.9%))
$\Delta E_{orb(7)}$[b]	-13.9 (5.8%)	-7.7 (3.4%)	-6.4 (3.2%)	-5.6 (2.9%)
$\Delta E_{orb(8)}$[b]	-7.5 (3.1%)	-6.2 (2.8%)	-5.2 (2.6%)	-4.5 (2.3%)
$\Delta E_{orb(9)}$[b]	-7.0 (2.9%)	-5.7 (2.6%)	-4.5 (2.2%)	-4.2 (2.2%)
$\Delta E_{orb(10)}$[b]	-4.9 (2.1%)	-5.3 (2.4%)	-4.8 (2.4%)	-4.1 (2.1%)
$\Delta E_{orb(rest)}$[b]	-69.1 (29.0%)	-83.8 (37.5%)	-80.0 (39.7%)	-82.0 (42.3%

[a]The values in parentheses give the percentage contribution to the total attractive interactions $\Delta E_{elstat} + \Delta E_{orb} + \Delta E_{disp}$

[b]The values in parentheses give the percentage contribution to the total orbital interactions ΔE_{orb}.

Table S15. The Cartesian coordinates of **1**-Ae at the BP86-D3(BJ)/def2-SVP level.

1-Mg

E = -2817.701781 au

0 1

Fe -1.10164500 -2.60237500 -0.17602000

Mg 0.07422300 0.42996200 0.02868100

O 0.73496400 2.31300600 -0.56882300

O 0.43046200 0.69975600 2.04107100

N 1.44847900 -0.81597000 -0.65602100

N -1.90069000 0.48328000 -0.26592400

C 0.87430400 -2.09943800 -0.64682000

C -3.07851700 -3.03759600 -0.17283300

H -3.52833300 -3.95551900 -0.57416800

C -2.59421700 -2.84850400 1.17023400

H -2.61571600 -3.59090700 1.97915100

C 0.20054600 -2.75356900 -1.75449000

H 0.05852200 -2.30709600 -2.74769300

C -2.29205700 -0.81993400 -0.00383200

C -2.85272400 -1.81448300 -0.90440800

H -3.08439500 -1.65158600 -1.96435900

C -2.08523600 -1.50368500 1.26308200

H -1.67909400 -1.02920500 2.16583900

C -2.62609100 1.22458800 -1.27294900

H -3.35287000 0.57534700 -1.81698500

H -1.94669300 1.63621000 -2.06397300

C 3.97324900 -0.54102200 -0.83251800

C -3.43323100 2.43308000 -0.69738400

C 0.78492000 -3.02031000 0.48001600

H 1.19324700 -2.83083900 1.48065900

C 0.11037500 -4.21771000 0.05577000

H -0.10481600 -5.09758700 0.67735100

C 2.57955500 -0.62824000 -1.53863000

H 2.48361200 0.31532900 -2.13784300

H 2.63802800 -1.45376100 -2.29058600

C -0.25048500 -4.05317900 -1.33053600

H -0.79362900 -4.78414700 -1.94443500

C 0.83586600 2.62123400 -1.98455400

H -0.12193800 3.07613600 -2.32282600

H 0.99333500 1.66392900 -2.52080600

C 4.20782200 -1.81204800 0.00054600

H 3.40515000 -1.92971700 0.75602500

H 5.18763900 -1.78046800 0.52389600

H 4.18587500 -2.71888000 -0.63956000

C 2.00270300 3.59950200 -2.07098700

H 1.97237000 4.21994900 -2.98801000

H 2.96430500 3.04453700 -2.05718700

C 5.06208200 -0.40401300 -1.91107800

H 5.08102600 -1.29499300 -2.57428700

H 6.07232500 -0.29561100 -1.46143700

H 4.88122000 0.48726500 -2.55162900

C 3.99866200 0.69435800 0.08543100

H 3.91468200 1.63077100 -0.50787500

H 4.93775300 0.75143000 0.67604500

H 3.14138500 0.66203600 0.78982900

C 1.82136400 4.41667900 -0.77808500

H 2.74951000 4.92155600 -0.44542100

H 1.04328500 5.19499500 -0.92270600

C 0.70675100 1.81444200 4.10273200

H 0.23478900 1.77793400 5.10420600

H 1.11783500 2.83444500 3.96813200

C -0.31618500 1.48092100 2.99719500

H -1.16144500 0.86544600 3.37863500

H -0.73943600 2.35096300 2.46093000

C 1.28459300 -0.17378200 2.81645600

H 2.03402900 -0.58717600 2.11553600

H 0.66451400 -1.00798600 3.21578800

C 1.34179300 3.36215700 0.22986300

H 2.17502600 2.90851200 0.80550000

H 0.58112800 3.75177500 0.93768300

C -4.43524700 1.91062900 0.34671900

H -5.15684200 1.20299400 -0.11369800

H -5.01307100 2.74045800 0.80733800

H -3.89626600 1.36272700 1.14645800

C -2.46584100 3.42712900 -0.02952000

H -1.88158200 2.90529500 0.75620200

H -3.00993400 4.27507900 0.43819800

H -1.74875800 3.84772500 -0.76757200

C -4.17381100 3.12625100 -1.85412000

H -3.46182600 3.47927400 -2.63223300

H -4.74915900 4.00795700 -1.49950500

H -4.88886600 2.43147600 -2.34434100

C 1.82234700 0.73949000 3.92654300

H 2.77277700 1.21042300 3.60545100

H 2.02576200 0.17478200 4.85722000

1-Ca

E = -6126.037138 au

0 1

Fe -1.02048300 3.52248200 -0.21518100

Ca -0.29128100 0.86423600 -1.54546000

O 0.87092900 0.27710300 -3.52803500

N 1.05278900 1.15086100 0.51348900

N -1.85088000 1.96450500 -2.95055500

C -1.32419100 1.83237800 0.89897400

H -1.79775700 0.87627300 0.63672000

C 0.11725900 2.09350900 0.92346000

C -2.01324100 2.98597800 1.43389300

H -3.09754800 3.07451700 1.58129700

C -0.29637200 3.72346300 -2.14730500

H 0.61513300 3.35491100 -2.63988300

C 0.27723800 3.44863500 1.41573400

H 1.22872800 3.97955000 1.52023800

C -1.01836500 3.97665700 1.75992900

H -1.21219300 4.96990900 2.18557200

C 2.45425000 1.40973200 0.82974900

C -1.79063600 5.16182500 -1.09566000

H -2.21420700 6.02164000 -0.55965900

C -3.23477400 1.58491600 -3.14531700

H -3.89699300 2.48196800 -3.22874100

H -3.64683300 0.99746300 -2.27770400

C -3.44698500 0.73558800 -4.42970500

C -2.52058800 4.00981900 -1.56103700

H -3.59097800 3.82975200 -1.40550800

C -1.62667300 3.14758000 -2.32878100

C 1.02526000 -0.87884100 -5.57453000

H 1.60487800 -1.62745800 -6.15000500

H -0.04665500 -0.99381900 -5.83532300

C -0.40516900 4.98282800 -1.44771200

H 0.41525900 5.68696200 -1.25642600

C 3.39984000 2.00353800 -0.25812300

C -4.93331700 0.34372800 -4.51637800

H -5.24155400 -0.24959800 -3.62866300

H -5.13508500 -0.27022000 -5.41963400

H -5.58447900 1.24250700 -4.56624400

C 0.99986700 1.30136500 -4.56113600

H 1.71920700 2.06948000 -4.20428100

H -0.00380300 1.76727700 -4.66622700

C 1.21681700 -1.02703300 -4.06720400

H 0.56278700 -1.77963200 -3.58612500

H 2.27027100 -1.26193500 -3.79693600

C 2.93578300 3.41053800 -0.66847100

H 1.86140600 3.40930500 -0.93332000

H 3.51876400 3.79240100 -1.53354700

H 3.06541000 4.13222900 0.16502000

C 1.48931100 0.56860900 -5.81741000

H 2.59643600 0.61105800 -5.88874900

H 1.07218400 1.01126100 -6.74331900

C 4.80964700 2.07649400 0.36200100

H 4.81439100 2.71425200 1.27143600

H 5.54070400 2.50540500 -0.35518500

H 5.17364700 1.06850400 0.65568800

C 3.44816300 1.08588400 -1.49118600

H 3.78358700 0.06259700 -1.21983400

H 4.15497200 1.48073600 -2.25132400

H 2.46154900 0.98798500 -1.98679600

C -2.58108400 -0.53573000 -4.37170500

H -1.52035400 -0.26930400 -4.19462200

H -2.64493900 -1.11030000 -5.32024900

H -2.90513600 -1.20659000 -3.55120200

C -3.05090000 1.57416000 -5.65917900

H -3.67908000 2.48669900 -5.73935600

H -3.16791700 0.99313600 -6.59878800

H -1.99553600 1.90384100 -5.57422800

H 2.92702000 0.44229700 1.13286500

H 2.53471400 2.06727500 1.72798100

Fe 1.02048300 -3.52248200 0.21518100

Ca 0.29128100 -0.86423600 1.54546000

O -0.87092900 -0.27710300 3.52803500

N -1.05278900 -1.15086100 -0.51348900

N 1.85088000 -1.96450500 2.95055500

C 1.32419100 -1.83237800 -0.89897400

H 1.79775700 -0.87627300 -0.63672000

C -0.11725900 -2.09350900 -0.92346000

C 2.01324100 -2.98597800 -1.43389300

H 3.09754800 -3.07451700 -1.58129700

C 0.29637200 -3.72346300 2.14730500

H -0.61513300 -3.35491100 2.63988300

C -0.27723800 -3.44863500 -1.41573400

H -1.22872800 -3.97955000 -1.52023800

C 1.01836500 -3.97665700 -1.75992900

H 1.21219300 -4.96990900 -2.18557200

C -2.45425000 -1.40973200 -0.82974900

C 1.79063600 -5.16182500 1.09566000

H 2.21420700 -6.02164000 0.55965900

C 3.23477400 -1.58491600 3.14531700

H 3.89699300 -2.48196800 3.22874100

H 3.64683300 -0.99746300 2.27770400

C 3.44698500 -0.73558800 4.42970500

C 2.52058800 -4.00981900 1.56103700

H 3.59097800 -3.82975200 1.40550800

C 1.62667300 -3.14758000 2.32878100

C -1.02526000 0.87884100 5.57453000

H -1.60487800 1.62745800 6.15000500

H 0.04665500 0.99381900 5.83532300

C 0.40516900 -4.98282800 1.44771200

H -0.41525900 -5.68696200 1.25642600

C -3.39984000 -2.00353800 0.25812300

C 4.93331700 -0.34372800 4.51637800

H 5.24155400 0.24959800 3.62866300

H 5.13508500 0.27022000 5.41963400

H 5.58447900 -1.24250700 4.56624400

C -0.99986700 -1.30136500 4.56113600

H -1.71920700 -2.06948000 4.20428100

H 0.00380300 -1.76727700 4.66622700

C -1.21681700 1.02703300 4.06720400

H -0.56278700 1.77963200 3.58612500

H -2.27027100 1.26193500 3.79693600

C -2.93578300 -3.41053800 0.66847100

H -1.86140600 -3.40930500 0.93332000

H -3.51876400 -3.79240100 1.53354700

H -3.06541000 -4.13222900 -0.16502000

C -1.48931100 -0.56860900 5.81741000

H -2.59643600 -0.61105800 5.88874900

H -1.07218400 -1.01126100 6.74331900

C -4.80964700 -2.07649400 -0.36200100

H -4.81439100 -2.71425200 -1.27143600

H -5.54070400 -2.50540500 0.35518500

H -5.17364700 -1.06850400 -0.65568800

C -3.44816300 -1.08588400 1.49118600

H -3.78358700 -0.06259700 1.21983400

H -4.15497200 -1.48073600 2.25132400

H -2.46154900 -0.98798500 1.98679600

C 2.58108400 0.53573000 4.37170500

H 1.52035400 0.26930400 4.19462200

H 2.64493900 1.11030000 5.32024900

H 2.90513600 1.20659000 3.55120200

C 3.05090000 -1.57416000 5.65917900

H 3.67908000 -2.48669900 5.73935600

H 3.16791700 -0.99313600 6.59878800

H 1.99553600 -1.90384100 5.57422800

H -2.92702000 -0.44229700 -1.13286500

H -2.53471400 -2.06727500 -1.72798100

1-Sr

E = -4832.376309 au

0 1

Sr 0.06287700 -1.83088900 -0.50384300

Fe 0.74653000 -2.58319100 2.52776500

O -1.30626200 -3.03973200 -2.24644000

N -1.22512900 -0.33555700 1.20422700

N 1.50264100 -3.89334200 -0.43433100

C -0.32462200 -0.71931200 2.18496400

C 1.28885800 -4.06898000 0.88820100

C 1.77614200 -1.02402200 3.24079600

H 2.85798000 -1.01727800 3.42893600

C 1.13058000 -0.62621800 2.00715100

H 1.65263800 -0.24004100 1.11845600

C 1.47528200 -4.31499300 3.23449000

H 1.91064000 -4.43631300 4.23567700

C -0.53208600 -1.26235900 3.51421200

H -1.50520000 -1.50285700 3.95560600

C -0.04103700 -4.21041600 1.47473100

H -0.96137500 -4.35036400 0.88787700

C -2.62477200 -0.19658300 1.58567200

C 0.74247600 -1.40263400 4.17054300

H 0.89758600 -1.75583700 5.19868500

C 0.07946300 -4.43806600 2.89590500

H -0.74080300 -4.69474300 3.57925600

C 2.19799500 -3.99418000 2.02873500

H 3.27680300 -3.80749700 1.96461500

C -3.64156400 -1.31300800 1.20244400

C 2.87460000 -3.70268200 -0.85112900

H 3.57418900 -4.39249300 -0.31384900

H 3.25430900 -2.66758100 -0.60609200

C 2.11304400 -3.02801000 -3.18017800

H 1.05875700 -3.25054500 -2.92464700

H 2.23536400 -3.18613200 -4.27269000

H 2.30210300 -1.95351000 -2.98365100

C -1.30910400 -4.49711100 -2.25372000

H -2.22804800 -4.85442900 -1.73466400

H -0.40682800 -4.81798300 -1.68859600

C 3.06979400 -3.92961100 -2.37520500

C -3.27867700 -2.63481500 1.89804500

H -2.21998100 -2.90323600 1.71450200

H -3.92287600 -3.46860400 1.54640000

H -3.40548500 -2.55570500 2.99805100

C -5.03073600 -0.83630700 1.67221500

H -5.03983700 -0.64676700 2.76660100

H -5.81027100 -1.59623400 1.45423000

H -5.32549800 0.10689700 1.16414600

C -3.67886800 -1.51068600 -0.32347100

H -3.85845400 -0.54983200 -0.85089100

H -4.49167600 -2.21121400 -0.61078400

H -2.74121200 -1.94690500 -0.72225700

C -1.30951400 -4.87247200 -3.73441600

H -0.27427500 -4.84479100 -4.13393400

H -1.72017700 -5.88536800 -3.91603500

C -2.16350500 -3.74794700 -4.34997300

H -1.99021600 -3.60759800 -5.43516500

H -3.24346100 -3.96108500 -4.20330600

C -1.74502900 -2.51896800 -3.53296400

H -0.89526400 -1.96674000 -3.99064900

H -2.56579600 -1.79462400 -3.35844400

C 2.77686300 -5.40528700 -2.70560600

H 3.48248900 -6.07967400 -2.17573500

H 2.86903000 -5.60134800 -3.79532200

H 1.75141100 -5.67405300 -2.38029200

C 4.52498800 -3.58256500 -2.73914900

H 4.74741900 -2.51567300 -2.52246000

H 4.72200500 -3.75656700 -3.81814300

H 5.24205600 -4.20259600 -2.15999700

H -2.72078100 -0.03466400 2.68690700

H -3.02478000 0.73596900 1.11352900

Sr -0.06287700 1.83088900 0.50384300

Fe -0.74653000 2.58319100 -2.52776500

O 1.30626200 3.03973200 2.24644000

N 1.22512900 0.33555700 -1.20422700

N -1.50264100 3.89334200 0.43433100

C 0.32462200 0.71931200 -2.18496400

C -1.28885800 4.06898000 -0.88820100

C -1.77614200 1.02402200 -3.24079600

H -2.85798000 1.01727800 -3.42893600

C -1.13058000 0.62621800 -2.00715100

H -1.65263800 0.24004100 -1.11845600

C -1.47528200 4.31499300 -3.23449000

H -1.91064000 4.43631300 -4.23567700

C 0.53208600 1.26235900 -3.51421200

H 1.50520000 1.50285700 -3.95560600

C 0.04103700 4.21041600 -1.47473100

H 0.96137500 4.35036400 -0.88787700

C 2.62477200 0.19658300 -1.58567200

C -0.74247600 1.40263400 -4.17054300

H -0.89758600 1.75583700 -5.19868500

C -0.07946300 4.43806600 -2.89590500

H 0.74080300 4.69474300 -3.57925600

C -2.19799500 3.99418000 -2.02873500

H -3.27680300 3.80749700 -1.96461500

C 3.64156400 1.31300800 -1.20244400

C -2.87460000 3.70268200 0.85112900

H -3.57418900 4.39249300 0.31384900

H -3.25430900 2.66758100 0.60609200

C -2.11304400 3.02801000 3.18017800

H -1.05875700 3.25054500 2.92464700

H -2.23536400 3.18613200 4.27269000

H -2.30210300 1.95351000 2.98365100

C 1.30910400 4.49711100 2.25372000

H 2.22804800 4.85442900 1.73466400

H 0.40682800 4.81798300 1.68859600

C -3.06979400 3.92961100 2.37520500

C 3.27867700 2.63481500 -1.89804500

H 2.21998100 2.90323600 -1.71450200

H 3.92287600 3.46860400 -1.54640000

H 3.40548500 2.55570500 -2.99805100

C 5.03073600 0.83630700 -1.67221500

H 5.03983700 0.64676700 -2.76660100

H 5.81027100 1.59623400 -1.45423000

H 5.32549800 -0.10689700 -1.16414600

C 3.67886800 1.51068600 0.32347100

H 3.85845400 0.54983200 0.85089100

H 4.49167600 2.21121400 0.61078400

H 2.74121200 1.94690500 0.72225700

C 1.30951400 4.87247200 3.73441600

H 0.27427500 4.84479100 4.13393400

H 1.72017700 5.88536800 3.91603500

C 2.16350500 3.74794700 4.34997300

H 1.99021600 3.60759800 5.43516500

H 3.24346100 3.96108500 4.20330600

C 1.74502900 2.51896800 3.53296400

H 0.89526400 1.96674000 3.99064900

H 2.56579600 1.79462400 3.35844400

C -2.77686300 5.40528700 2.70560600

H -3.48248900 6.07967400 2.17573500

H -2.86903000 5.60134800 3.79532200

H -1.75141100 5.67405300 2.38029200

C -4.52498800 3.58256500 2.73914900

H -4.74741900 2.51567300 2.52246000

H -4.72200500 3.75656700 3.81814300

H -5.24205600 4.20259600 2.15999700

H 2.72078100 0.03466400 -2.68690700

H 3.02478000 -0.73596900 -1.11352900

1-Ba

E = -5286.561130 au

0 1

Ba 0.33187500 -2.03368700 -0.38955300

Fe 3.12916800 -0.84145200 -2.00242100

N 0.11900200 0.37433900 -1.75424600

O -1.17440500 -4.09583400 0.78095500

N 2.35636800 -3.80386500 -0.54049000

C 1.48176700 0.57240300 -1.79223700

C -0.53943400 0.13056300 -3.02771300

H -1.11062500 -0.83203700 -2.98345400

H 0.19963600 -0.02311900 -3.84894800

C 2.33655900 0.47346200 -0.60230700

H 1.99434300 0.26052000 0.42123700

C -1.55972300 1.20986600 -3.49653200

C 3.10706600 -4.26313600 0.60992300

H 2.42957400 -4.30978200 1.49896400

H 3.92648500 -3.56332400 0.91118000

C -2.75907800 1.27726200 -2.53252200

H -3.24296600 0.28355000 -2.42474400

H -3.52661000 1.99084000 -2.89734200

H -2.48414800 1.61286400 -1.51307200

C 3.03185700 -3.08807500 -1.47115300

C 2.47406000 -2.67816800 -2.75865500

C 3.70496900 0.77505500 -0.97575400

H 4.56215000 0.83054800 -0.29154000

C 2.37475000 0.86948100 -2.89915400

H 2.07437800 0.97344800 -3.94836900

C 3.50644000 -2.04316200 -3.54714600

H 3.42020700 -1.74796300 -4.60159500

C 4.33852700 -2.43351000 -1.41117700

H 5.02030500 -2.46466600 -0.55322800

C 4.67138800 -1.90757100 -2.70952300

H 5.63664500 -1.47459300 -3.00583000

C -2.68769000 -3.16189100 -2.23092800

H -3.02465300 -2.34625400 -2.90490200

H -2.97452800 -2.88902400 -1.19072200

C -2.07169300 0.79882400 -4.89029700

H -1.24032600 0.74939300 -5.62498800

H -2.82398600 1.51986000 -5.27395700

H -2.55113000 -0.20351000 -4.85784400

C 4.75319900 -5.70018400 -0.70139600

H 4.25802900 -5.44032300 -1.65866700

H 5.21058900 -6.70703500 -0.81059700

H 5.57197900 -4.96909800 -0.53949000

C -0.87159400 2.58172200 -3.57716000

H -0.38810400 2.83967200 -2.61300200

H -1.59546400 3.38555100 -3.82689000

H -0.07303700 2.58341000 -4.34842800

C 3.74630800 -5.68414700 0.46351000

C 3.71780900 1.03027200 -2.39880500

H 4.59758700 1.30211600 -2.99800400

C -0.83595300 -4.62583600 -2.54532300

H 0.05716100 -4.83766000 -1.91721100

H -0.54252300 -4.73259200 -3.61476400

C 4.46898000 -6.01525900 1.78204100

H 5.28026200 -5.28459200 1.98911400

H 4.92618000 -7.02714400 1.75004600

H 3.76521800 -5.99025700 2.64261000

C -3.21636700 -4.54523400 -2.63081400

H -3.37129100 -4.59996000 -3.72911400

H -4.17994700 -4.78623800 -2.13953500

C 2.64818000 -6.72967900 0.19432800

H 1.97671200 -6.84333900 1.07214100

H 3.08795200 -7.72749600 -0.01737000

H 2.03409800 -6.42097100 -0.67648300

C -2.48156100 -4.09341800 1.38342200

H -3.14168000 -4.80643000 0.83031300

H -2.89293900 -3.07049100 1.30279600

C -0.52186200 -5.29194500 1.24604800

H 0.56657400 -5.13777500 1.13425300

H -0.81395500 -6.14680700 0.59087200

C -1.01121900 -5.50626800 2.69779800

H -0.22371400 -5.24333800 3.43023000

H -1.28255200 -6.56706300 2.86906600

C -2.23583400 -4.55715600 2.82370400

H -3.12551500 -5.05043800 3.26258300

H -1.99332600 -3.67490000 3.44806300

O -1.24347700 -3.25263900 -2.30682800

C -2.06310400 -5.47162100 -2.20520400

H -2.06689300 -6.44980800 -2.72554600

H -2.10104000 -5.65729800 -1.11268500

H 1.49743100 -2.99764300 -3.15052100

Ba -0.33187500 2.03368700 0.38955300

Fe -3.12916800 0.84145200 2.00242100

N -0.11900200 -0.37433900 1.75424600

O 1.17440500 4.09583400 -0.78095500

N -2.35636800 3.80386500 0.54049000

C -1.48176700 -0.57240300 1.79223700

C 0.53943400 -0.13056300 3.02771300

H 1.11062500 0.83203700 2.98345400

H -0.19963600 0.02311900 3.84894800

C -2.33655900 -0.47346200 0.60230700

H -1.99434300 -0.26052000 -0.42123700

C 1.55972300 -1.20986600 3.49653200

C -3.10706600 4.26313600 -0.60992300

H -2.42957400 4.30978200 -1.49896400

H -3.92648500 3.56332400 -0.91118000

C 2.75907800 -1.27726200 2.53252200

H 3.24296600 -0.28355000 2.42474400

H 3.52661000 -1.99084000 2.89734200

H 2.48414800 -1.61286400 1.51307200

C -3.03185700 3.08807500 1.47115300

C -2.47406000 2.67816800 2.75865500

C -3.70496900 -0.77505500 0.97575400

H -4.56215000 -0.83054800 0.29154000

C -2.37475000 -0.86948100 2.89915400

H -2.07437800 -0.97344800 3.94836900

C -3.50644000 2.04316200 3.54714600

H -3.42020700 1.74796300 4.60159500

C -4.33852700 2.43351000 1.41117700

H -5.02030500 2.46466600 0.55322800

C -4.67138800 1.90757100 2.70952300

H -5.63664500 1.47459300 3.00583000

C 2.68769000 3.16189100 2.23092800

H 3.02465300 2.34625400 2.90490200

H 2.97452800 2.88902400 1.19072200

C 2.07169300 -0.79882400 4.89029700

H 1.24032600 -0.74939300 5.62498800

H 2.82398600 -1.51986000 5.27395700

H 2.55113000 0.20351000 4.85784400

C -4.75319900 5.70018400 0.70139600

H -4.25802900 5.44032300 1.65866700

H -5.21058900 6.70703500 0.81059700

H -5.57197900 4.96909800 0.53949000

C 0.87159400 -2.58172200 3.57716000

H 0.38810400 -2.83967200 2.61300200

H 1.59546400 -3.38555100 3.82689000

H 0.07303700 -2.58341000 4.34842800

C -3.74630800 5.68414700 -0.46351000

C -3.71780900 -1.03027200 2.39880500

H -4.59758700 -1.30211600 2.99800400

C 0.83595300 4.62583600 2.54532300

H -0.05716100 4.83766000 1.91721100

H 0.54252300 4.73259200 3.61476400

C -4.46898000 6.01525900 -1.78204100

H -5.28026200 5.28459200 -1.98911400

H -4.92618000 7.02714400 -1.75004600

H -3.76521800 5.99025700 -2.64261000

C 3.21636700 4.54523400 2.63081400

H 3.37129100 4.59996000 3.72911400

H 4.17994700 4.78623800 2.13953500

C -2.64818000 6.72967900 -0.19432800

H -1.97671200 6.84333900 -1.07214100

H -3.08795200 7.72749600 0.01737000

H -2.03409800 6.42097100 0.67648300

C 2.48156100 4.09341800 -1.38342200

H 3.14168000 4.80643000 -0.83031300

H 2.89293900 3.07049100 -1.30279600

C 0.52186200 5.29194500 -1.24604800

H -0.56657400 5.13777500 -1.13425300

H 0.81395500 6.14680700 -0.59087200

C 1.01121900 5.50626800 -2.69779800

H 0.22371400 5.24333800 -3.43023000

H 1.28255200 6.56706300 -2.86906600

C 2.23583400 4.55715600 -2.82370400

H 3.12551500 5.05043800 -3.26258300

H 1.99332600 3.67490000 -3.44806300

O 1.24347700 3.25263900 2.30682800

C 2.06310400 5.47162100 2.20520400

H 2.06689300 6.44980800 2.72554600

H 2.10104000 5.65729800 1.11268500

H -1.49743100 2.99764300 3.15052100

3. References

[S1] J. Oetzel, N. Weyer, C. Bruhn, M. Leibold, B. Gerke, R. Pöttgen, M. Maier, R. F. Winter, M. C. Holthausen, U. Siemling, *Chem Eur. J.* **2017**, *23*, 1187-1199.

[S2] A. R. Petrov, K. Jess, M. Freytag, P. G. Jones, M. Tamm, *Organometallics* **2013**, *32*, 5946-5954.

[S3] M. Westerhausen, *Inorg. Chem.* **1991**, *30*, 96-101.

[S4] (a) Agilent, **2014**, CrysAlisPro Software system, version 1.171.37.35, Agilent Technologies Ltd, Yarnton, Oxfordshire, England; (b) Rigaku Oxford Diffraction, **2019**, CrysAlisPro Software system, version 1.171.40.53, Rigaku Corporation, Oxford, UK.

[S5] O. V. Dolomanov, L. J. Bourhis, R. J. Gildea, J. A. K. Howard, H. Puschmann, *J. Appl. Cryst.* **2009**, *42*, 339-341.

[S6] G. M. Sheldrick, *Acta Cryst. A*, **2015**, *71*, 3–8.

[S7] G. M. Sheldrick, *Acta Cryst. C*, **2015**, *71*, 3–8.

[S8] A. Thorn, B. Dittrich, G. M. Sheldrick, *Acta Cryst. A*, **2012**, *68*, 448–451.

[S9] M. Sato, H. Shigeta, M. Sekino, S. Akabori, *J. Organomet. Chem.* **1993**, *458*, 199-204

[S10] a) A. D. Becke, *Phys. Rev. A* **1988**, *38*, 3098–3100; b) J. P. Perdew, *Phys. Rev. B* **1986**, *33,* 8822–8824; c) S. Grimme, S. Ehrlich, L. Goerigk, *J. Comput. Chem.* **2011**, *32,* 1456–1465; d) S. Grimme, J. Antony, S. Ehrlich, H. Krieg, *J. Chem. Phys.* **2010**, *132,* 154104; e) F. Weigend, R. Ahlrichs, *Phys. Chem. Chem. Phys.* **2005**, *7*, 3297–3305.

[S11] Gaussian 16, Revision A.03, M. J. Frisch, G. W. Trucks, H. B. Schlegel, G. E. Scuseria, M. A. Robb, J. R. Cheeseman, G. Scalmani, V. Barone, G. A. Petersson, H. Nakatsuji, X. Li, M. Caricato, A. V. Marenich, J. Bloino, B. G. Janesko, R. Gomperts, B. Mennucci, H. P. Hratchian, J. V. Ortiz, A. F. Izmaylov, J. L. Sonnenberg, D. Williams-Young, F. Ding, F. Lipparini, F. Egidi, J. Goings, B. Peng, A. Petrone, T. Henderson, D. Ranasinghe, V. G. Zakrzewski, J. Gao, N. Rega, G.

Zheng, W. Liang, M. Hada, M. Ehara, K. Toyota, R. Fukuda, J. Hasegawa, M. Ishida, T. Nakajima, Y. Honda, O. Kitao, H. Nakai, T. Vreven, K. Throssell, J. A. Montgomery, Jr., J. E. Peralta, F. Ogliaro, M. J. Bearpark, J. J. Heyd, E. N. Brothers, K. N. Kudin, V. N. Staroverov, T. A. Keith, R. Kobayashi, J. Normand, K. Raghavachari, A. P. Rendell, J. C. Burant, S. S. Iyengar, J. Tomasi, M. Cossi, J. M. Millam, M. Klene, C. Adamo, R. Cammi, J. W. Ochterski, R. L. Martin, K. Morokuma, O. Farkas, J. B. Foresman, and D. J. Fox, Gaussian, Inc., Wallingford CT, **2016**.

[S12] a) F. Weinhold, C. R. Landis, *Discovering Chemistry With Natural Bond Orbitals*. Wiley, New Jersey, **2012**; c) F. Weinhold, C. R. Landis, *Valency and Bonding: A Natural Bond Orbital Donor-Acceptor Perspective.* Cambridge University Press, Cambridge, **2005**.

[S13] E. D. Glendening, C. R. Landis, C.F. Weinhold, F. *J. Comput. Chem.* **2013,** *34*, 1429.

[S14] R. F. W. Bader, *Atoms in Molecules: A Quantum Theory*. Clarendon Press: Oxford, UK, 1990.

[S15] AIMAll (Version 17.11.14), T. A. Keith, TK Gristmill Software, Overland Park KS, USA, (aim.tkgristmill.com). (2017).

[S16] T. Ziegler, A. Rauk, *Theor. Chim. Acta* **1977**, *46*, 1-10.

[S17] a) M. Mitoraj, A. Michalak, *Organometallics* **2007**, *26*, 6576; b) M. Mitoraj, A. Michalak, *J. Mol. Model.* **2008**, *14*, 681.

[S18] a) ADF2018, SCM, Theoretical Chemistry, Vrije Universiteit, Amsterdam, The Netherlands, http://www.scm.com; b) G. te Velde, F. M. Bickelhaupt, E. J. Baerends, C. F. Guerra, S. J. A. Van Gisbergen, J. G. Snijders, T. Ziegler, *J. Comput. Chem*. **2001**, *22*, 931.

[S19] E. van Lenthe, E. J. Baerends, *J. Comput. Chem*. **2003**, *24*, 1142.

[S20] a) G. Frenking, F. M. Bickelhaupt, in *The Chemical Bond. Fundamental Aspects of Chemical Bonding*, G. Frenking and S. Shaik (Eds), Wiley-VCH, Weinheim, **2014**, p. 121-158; b) G. Frenking, R. Tonner, S. Klein, N. Takagi, T. Shimizu, A.

Krapp, K. K. Pandey, P. Parameswaran, *Chem. Soc. Rev*. **2014**, *43*, 5106; c) L. Zhao, M. Hermann, N. Holzmann, G. Frenking, *Coord. Chem. Rev*. **2017**, *344*, 163; d) G. Frenking, M. Hermann, D. M. Andrada, N. Holzmann, *Chem. Soc. Rev*. **2016**, *45*, 1129; e) L. Zhao, S. Pan, N. Holzmann, P. Schwerdtfeger, G. Frenking, *Chem. Rev*. **2019**, *119*, 8781.
